# Coordination of endothelial cell positioning and fate specification by the epicardium

**DOI:** 10.1038/s41467-021-24414-z

**Published:** 2021-07-06

**Authors:** Pearl Quijada, Michael A. Trembley, Adwiteeya Misra, Jacquelyn A. Myers, Cameron D. Baker, Marta Pérez-Hernández, Jason R. Myers, Ronald A. Dirkx, Ethan David Cohen, Mario Delmar, John M. Ashton, Eric M. Small

**Affiliations:** 1 Department of Medicine, Aab Cardiovascular Research Institute, University of Rochester School of Medicine and Dentistry, Rochester, NY USA; 2 Department of Biomedical Engineering, University of Rochester, Rochester, NY USA; 3 Genomics Research Center, University of Rochester School of Medicine and Dentistry, Rochester, NY USA; 4 Department of Microbiology and Immunology, University of Rochester School of Medicine and Dentistry, Rochester, NY USA; 5 Leon H. Charney Division of Cardiology, Department of Medicine, New York University School of Medicine, New York, NY USA; 6 Department of Pediatrics, University of Rochester School of Medicine and Dentistry, Rochester, NY USA; 7 Department of Pharmacology and Physiology, University of Rochester, Rochester, NY USA; 8 Present Address: Department of Integrative Biology and Physiology, Molecular Biology Institute, Eli & Edythe Broad Center of Regenerative Medicine and Stem Cell Research, UCLA Cardiovascular Theme, David Geffen School of Medicine, University of California, Los Angeles, CA USA

**Keywords:** Mouse, Functional clustering, Cell lineage, Epithelial-mesenchymal transition, Transcriptomics

## Abstract

The organization of an integrated coronary vasculature requires the specification of immature endothelial cells (ECs) into arterial and venous fates based on their localization within the heart. It remains unclear how spatial information controls EC identity and behavior. Here we use single-cell RNA sequencing at key developmental timepoints to interrogate cellular contributions to coronary vessel patterning and maturation. We perform transcriptional profiling to define a heterogenous population of epicardium-derived cells (EPDCs) that express unique chemokine signatures. We identify a population of Slit2+ EPDCs that emerge following epithelial-to-mesenchymal transition (EMT), which we term vascular guidepost cells. We show that the expression of guidepost-derived chemokines such as Slit2 are induced in epicardial cells undergoing EMT, while mesothelium-derived chemokines are silenced. We demonstrate that epicardium-specific deletion of myocardin-related transcription factors in mouse embryos disrupts the expression of key guidance cues and alters EPDC-EC signaling, leading to the persistence of an immature angiogenic EC identity and inappropriate accumulation of ECs on the epicardial surface. Our study suggests that EC pathfinding and fate specification is controlled by a common mechanism and guided by paracrine signaling from EPDCs linking epicardial EMT to EC localization and fate specification in the developing heart.

## Abstract (web summary)

It remains unclear how spatial information controls endothelial cell identity and behavior in the developing heart. Here the authors perform single cell RNA sequencing at key developmental timepoints in mice to interrogate cellular contributions to coronary vessel patterning and maturation in the epicardium.

## Introduction

Coronary endothelial cells (ECs) organize into an intricate vascular network that provides the heart with oxygen and nutrients and removes metabolic waste. During embryonic development, localized specification of immature ECs into arterial and venous fates occurs within the compact myocardium and sub-epicardium, respectively^1^. EC fate specification and maturation are accomplished through cell-intrinsic transcriptional programs that facilitate appropriate interconnections of the blood-supplying and blood-draining vascular beds^2^. Analyses of intersomitic and retinal vessel development identified a population of endothelial tip cells that are particularly responsive to secreted angiogenic factors such as vascular endothelial growth factor (Vegf), highlighting the functional heterogeneity of ECs and revealing mechanisms governing vascular pathfinding, remodeling, and maturation^3^. Clinical studies aimed at improving perfusion of the ischemic heart and skeletal muscle have attempted to recapitulate these developmental angiogenic programs^4^. However, the mechanisms through which coronary ECs coordinate positional information and fate specification remain elusive, and current therapeutic strategies have failed to generate a robust, functional vascular network.

The epicardium consists of a single layer of mesothelial cells on the surface of the heart that harbors a population of multipotent progenitors. Following epithelial-to-mesenchymal transition (EMT), epicardium-derived cells (EPDCs) migrate into the compact myocardium and differentiate into cardiac fibroblast and vascular mural cell lineages^5–7^. Construction of the coronary plexus requires the integration of epicardium-derived mural cells with arterial and venous ECs derived from the sinus venosus and endocardium^5,8,9^. Genetic or mechanical disruption of the epicardium has also revealed important paracrine contributions to cardiomyocyte growth^10^ and coronary plexus formation^11,12^. Our previous study found that epicardial EMT is required for coronary blood vessel maturation and integrity, at least partially via contributing vascular pericytes to the growing plexus^7^.

In this study, we performed single-cell RNA-sequencing of EPDCs and coronary ECs at critical developmental stages to gain insight into the mechanisms responsible for patterning of the developing coronary vasculature via distinct epicardial cell populations^13–15^. We found that epicardial EMT is not only responsible for the differentiation of EPDCs into vascular mural lineages^7^, but also restricts the expression of chemotactic signals to discrete populations of mural cells that provide detailed positional information, reminiscent of the guidepost neuron^16^. Genetic disruption of epicardial EMT in mice leads to profound alterations in EC developmental trajectory, which includes the accumulation of an immature EC population within the sub-epicardium. Importantly, EC maturation and migration are both directly controlled by angiogenic chemokines, providing a paradigm that coordinates EC localization and arteriovenous (AV) specification. Harnessing the principles that define the spatial architecture of the developing coronary vasculature may provide strategies to stimulate angiogenesis and improve perfusion of ischemic heart tissue, a limiting aspect of regenerative medicine approaches.

## Results

### Single-cell analysis of epicardium-derived cell heterogeneity

**Fig. 1:**
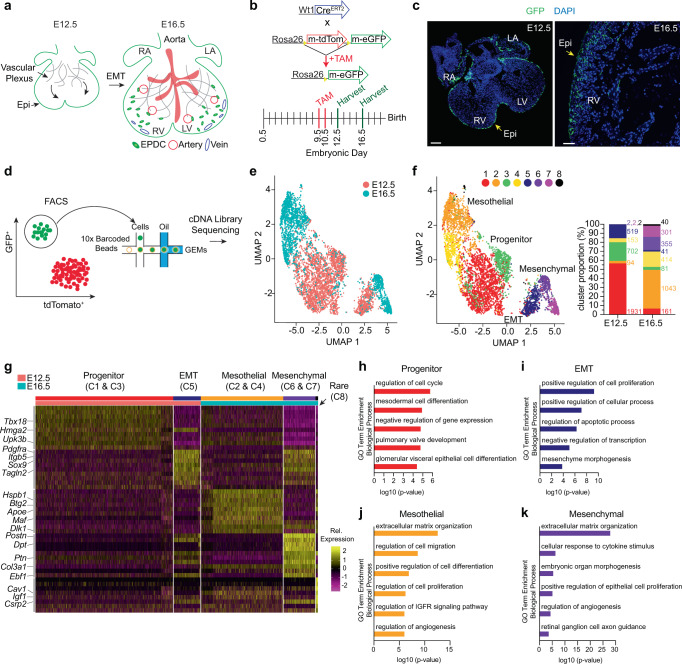
Characterization of epicardial cell heterogeneity in the fetal heart. **a** Schematic of epicardium and vasculature development. **b** Experimental strategy for lineage tracing epicardium-derived cells in Wt1^CreERT2^;R26^mTmG^ embryos. TAM was administered to pregnant dams at embryonic day (E)9.5 and E10.5 and embryos were harvested at E12.5 and E16.5. **c** Representative images of mouse embryos at embryonic stage E12.5 and E16.5 used for collection of GFP^+^ epicardial cells (green). DAPI staining was utilized to visualize nuclei (blue). Scale bar is 100 μm (E12.5) and 50 μm (E16.5). Immunostaining was repeated independently 3 times with similar results. **d** FACS-based enrichment of *Wt1*-lineage-derived epicardial cells prior to single-cell capture using 10× Genomics Chromium Controller. Refer to Supplementary Fig. 1c, d for FACS sequential gating and enrichment of epicardial cells. **e** and **f** UMAP of single-cell transcriptomes of E12.5 (3405 cells) and E16.5 (2436 cells) epicardial cells presented by **e** developmental stage and **f** cell identity, with the relative contribution of each cell cluster within each developmental stage indicated on the right. The number of cells per cluster (C) is listed next to the corresponding color in the graph. **g** Hierarchical clustering of differentially expressed genes by cell identity represented as a heat map. Relative (Rel.) expression represents scaled normalized expression. **h**–**k** GO analysis indicates most enriched biological processes within cells defined as **h** progenitor (C1 and C3), **i** EMT (C5), **j** mesothelial (C2 and C4), and **k** mesenchymal (C6 and C7). GO-term enrichment significance was determined using Fisher exact test. Epi epicardium, EMT epithelial-to-mesenchymal transition, RA right atrium, RV right ventricle, LA left atrium, LV left ventricle, m-tdTom membrane tdTomato, m-eGFP membrane-enhanced green fluorescent protein, TAM tamoxifen, GEMs Gel Bead-in-Emulsion.

Coronary endothelial cell AV specification and integration of the arterial and venous vasculature coincides temporally with epicardial EMT, between embryonic day (E) 12.5 and E16.5^9^ (Fig. 1a). To investigate epicardial contributions to the growing coronary plexus at these timepoints, GFP-positive (GFP^+^) EPDCs were isolated from Wt1^CreERT2/+^;Rosa^mTmG^ mouse embryos by fluorescence-activated cell sorting (FACS) (Fig. 1b, c and Supplementary Fig. 1a–d). GFP^+^ cells displayed epicardial gene enrichment (*Aldh1a2*, *Tbx18*, *Tcf21*, *Wt1*) and did not express high levels of cardiomyocyte genes (*Tnnt2*, *Myh7*) (Supplementary Fig. 1e). Increased expression of the mesenchymal cell marker *Pdgfra* was observed in a number of GFP^+^ cells at E16.5, consistent with the acquisition of a motile phenotype and differentiation into interstitial cell types (Supplementary Fig. 1f–h).

Single-cell RNA-sequencing (scRNA-seq) was performed on EPDCs captured using the 10× Genomics platform (Fig. 1d). We excluded cell doublets based upon unique molecular identifier counts, and mitochondrial and ribosomal gene expression patterns were analyzed and filtered to obtain 3405 (E12.5) and 2436 (E16.5) single EPDCs (Supplementary Fig. 2a, b). To define the cellular heterogeneity within the epicardium, we performed an integration of E12.5 and E16.5 data sets using canonical correlation analysis (CCA) followed by uniform manifold approximation and projection (UMAP) using Seurat to rule out batch effects (Supplementary Fig. 3a–d), and present a merged analysis of E12.5 and E16.5 cells (Fig. 1e). This analysis revealed 8 distinct populations (C1-C8) with a considerable contribution of proliferation state and developmental age to cellular phenotype (Fig. 1e, f and Supplementary Fig. 4a, b). To facilitate the identification of epicardial cell phenotypes, E12.5 and E16.5 data were merged and a CCA was performed with previously published scRNA-seq data obtained from the postnatal day 1 mouse heart^17^ (Supplementary Fig. 4c, d). Within these populations, five broad identities emerged, consistent with marker genes identified by hierarchical clustering and gene ontology (GO) analysis (Fig. 1g–k and Supplementary 5a, b; Supplementary Dataset 1): (1) early developmental stage progenitor (C1, C3); (2) early EMT (C5); (3) late developmental stage mesothelial (C2, C4); (4) late developmental stage EMT/mesenchymal (C6, C7); and (5) a rare population of approximately 40 cells (C8, 1.64% of total) that display an enrichment in IGF pathway genes and express high levels of *Cav1* previously implicated in zebrafish heart regeneration^13^.

EPDCs in C1 and C3 are characterized by robust expression of epicardial gene markers *Tbx18* and *Upk3b*^18,19^, and GO terms associated with proliferation and epithelial cell differentiation, defining populations of self-replicating E12.5 mesothelial cells (Fig. 1g, h). In contrast, C5 is enriched in E12.5 EPDCs that exhibit a predisposition towards EMT prior to the acquisition of a motile gene program, based on the expression of early markers of the mesenchymal and smooth muscle phenotype, including *Pdgfra*, *Itgb5*, *Sox9,* and *Tagln2*^20,21^ (Fig. 1g, i). C2 and C4 contain an over-abundance of E16.5 EPDCs that express genes related to maintenance of mesothelial characteristics (*Maf*, *Btg2*), likely representing cells that remain on the cardiac surface^22,23^ (Fig. 1g, j). Genes encoding extracellular matrix proteins such as *Postn*, *Dpt*, and *Col3a1* are robustly expressed in E16.5 EPDCs in C6 and C7, consistent with the acquisition of a mesenchymal phenotype^20^ (Fig. 1g, k). Of note, these data also revealed unique vascular programs based upon the emergence of angiogenesis among the most enriched GO terms at E16.5, and the enrichment of *Hspb1* and *Dlk1*^24,25^ in mesothelial cells and *Ramp2* and *Sfrp2*^26,27^ in mesenchymal cells (Fig. 1g, j, k and Supplementary Fig. 5a).

### Distinct epicardium-derived vascular guidance programs

**Fig. 2:**
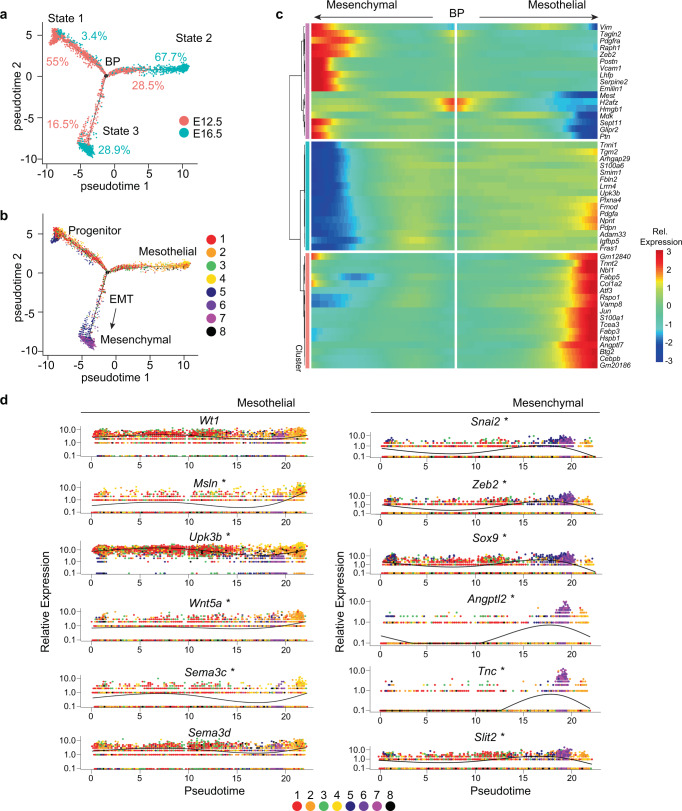
Epicardial-to-mesenchymal transition restricts expression of secreted ligands genes. **a** Monocle-generated pseudotime trajectory reveals the composition of three cell states based on the developmental stage (%). Cells in state 1 are at the beginning of pseudotime. Cell states 2 and 3 diverge at a common branchpoint (BP). **b** Pseudotime trajectory reveals the composition of cell identity within cell states. State 1 contains epicardial progenitor cells from Cluster 1 (C1)/C3. State 2 is populated by mesothelial cells from C2/C4. State 3 emerges from a common progenitor through a transient state characterized by cells in C5 actively undergoing EMT and culminates in mesenchymal cells of C6/C7. **c** Hierarchical clustering visualization of genes that define pseudotime states. Relative (Rel.) expression represents the scaled expression of rlog normalized data. **d** Pseudotime-dependent genes augmented in mesenchymal versus mesothelial cells. Cells are colored according to cell cluster identity. *Genes with significant correlation with pseudotime state.

We next evaluated the divergence of mesothelium and mesenchyme from a common epicardial progenitor, and the potential contribution of these distinct cellular populations to angiogenic processes, by assessing pseudotime trajectory using Monocle, an unsupervised learning algorithm that identifies branch points and cell commitment decisions^28,29^. We observed an abundance of E12.5 progenitor cells at the start of pseudotime (primarily C1, C3), that transition to cell states ultimately dominated by E16.5 EPDCs (Fig. 2a, b and Supplementary Fig. 6a, b). Cell state 2 is enriched in E16.5 mesothelial cells (C2, C4), while cell state 3 is composed of mesenchymal cells (C6, C7) following an early EMT intermediate (C5) (Fig. 2b). Analysis of pseudotime kinetics of differentially regulated genes across cell states revealed that mesothelial cells within state 2 maintain expression of genes such as *Wt1*, *Msln*, and *Upk3b*^18^, while cells in state 3 show downregulated expression *Msln* and *Upk3,b* as well as *Pdpn* and *Pdgfa*^30,31^, markers that are generally associated with the mesothelium (Fig. 2c). Instead, state 3 cells are enriched in EMT and mesenchyme genes such as *Snai2*, *Zeb2*, *Sox9*, *Postn, and Ptn*^20^ (Fig. 2c, d and Supplementry Fig. 6c). To further interrogate the angiogenic programs we identified in mature mesothelial and mesenchymal cells, we probed pseudotime kinetics for genes encoding axon guidance cues that regulate neuronal and vascular migration and pathfinding during development^32^. Among the more restricted guidance genes, we found epicardium-derived mesenchymal cells express an over-abundance of *Angptl2, Tnc*, and *Slit2*, whereas epicardium-derived mesothelial cells display an enrichment in *Wnt5a*, *Sema3c*, and *Sema3d* (Fig. 2d and Supplementary Fig. 6c; Supplementary Dataset 2).

**Fig. 3:**
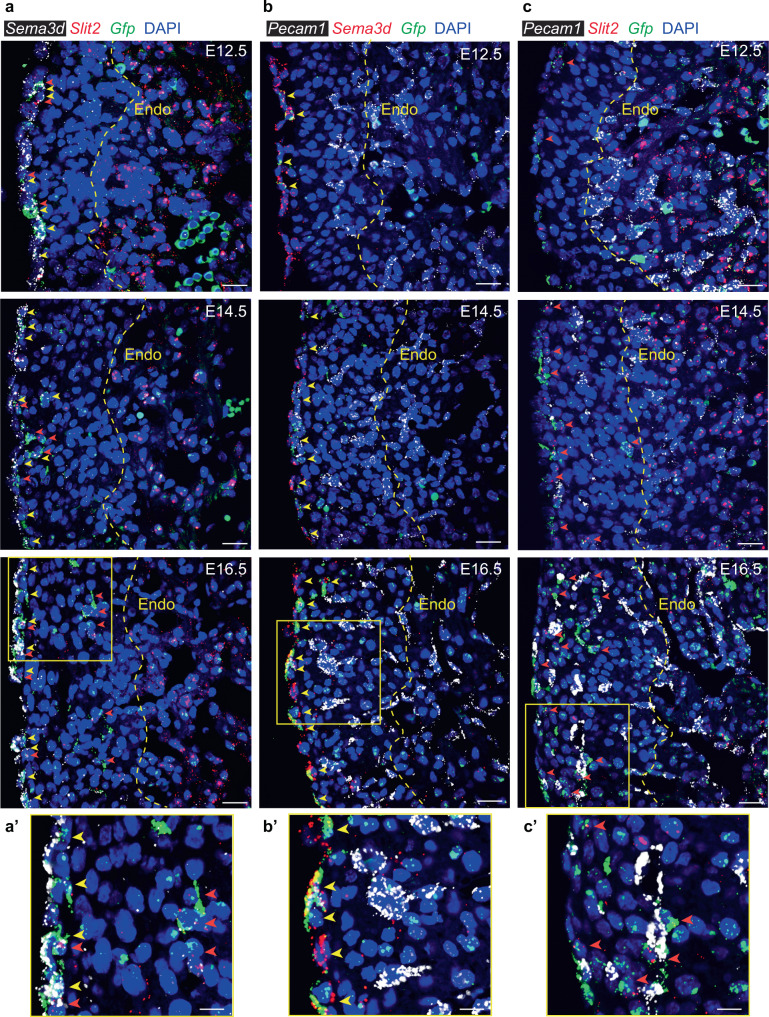
In situ analysis confirms the expression and localization of vascular guidance genes in epicardium-derived cells. **a** FISH using probes against *Gfp* (green) to detect epicardium-derived cells and *Sema3d* (white) or *Slit2* (red) reveals diverging localization of vascular guidance cues within the epicardium and interstitium between embryonic (E)12.5 and E16.5. **a**′, 3× zoom of E16.5. **b** Expression of *Sema3d* (red) in *Wt1* lineage-derived cells (*Gfp*^*+*^, green) relative to *Pecam1*^+^ (platelet endothelial cell adhesion molecule-1, white) endothelial cells. **b**′, 3× zoom of E16.5. **c** Expression of *Slit2* (red) in *Wt1* lineage-derived cells (*Gfp*^*+*^*, green*) relative to *Pecam1*^+^ (white) endothelial cells. **c**′ 3× zoom of E16.5. Yellow arrowhead, *Gfp*^*+*^*/Sema3d*^*+*^ cells. Orange arrowhead, *Gfp*^*+*^*/Slit2*^*+*^ cells. Dashed yellow line represents the endocardium–myocardium border. DAPI staining was utilized to visualize nuclei (blue). Scale bar, 20 μm. Scale bar 3× zoom, 10 μm. Endo endocardium.

To confirm that expression of *Slit2* and *Sema3d* is enriched in epicardium-derived mesenchyme and mesothelium, respectively, we performed RNAscope multiplex fluorescence in situ hybridization (FISH) on heart sections obtained from Wt1^CreERT2^;R26R^mTmG^ embryos labeled at E9.5/E10.5 and collected at E12.5 (pre-EMT), E14.5 (mid-EMT), and E16.5 (post-EMT). *Sema3d* is observed broadly within GFP^+^ mesothelial cells on the epicardial surface at all time points examined (Fig. 3a, b and Supplementary Fig. 7a, b). In contrast, *Slit2* is observed on the epicardial surface at E12.5, and is also expressed within a discrete population of epicardium-derived mesenchymal cells that are closely associated with *Pecam1*-expressing EC as early as E14.5, and most notably at E16.5 (Fig. 3a, c and Supplementary Fig. 7a, c). Of note, the expression of key angiogenic factors often increased in GFP^+^ EPDCs between E12.5 and E16.5, regardless of localization to mesothelial or mesenchymal cell populations (Supplementary Fig. 7d). The striking restriction of genes encoding secreted vascular guidance cues to distinct populations of EPDCs led us to speculate that angiogenic cues may be coordinately regulated with epicardial cell fate.

### Disruption of epicardial EMT alters expression of vascular guidance cues

**Fig. 4:**
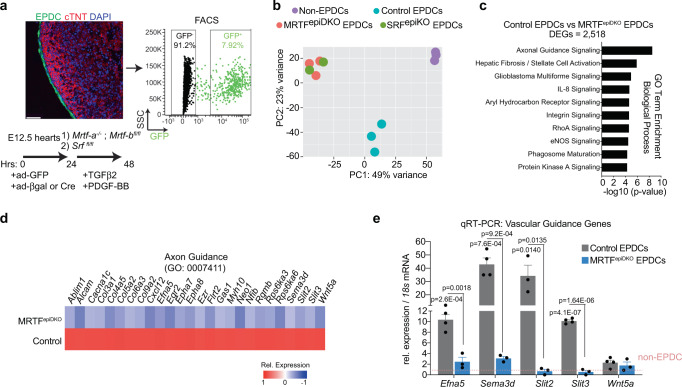
Genetic disruption of epithelial-to-mesenchymal transition alters the expression of vascular guidance cues. **a** Schematic of experimental design to isolate epicardial cells (EPDC) of indicated genotypes for bulk RNA-seq. Embryonic day (E)12.5 hearts were removed from Mrtf-a^−/−^;Mrtf-b^fl/fl^, and Srf^fl/fl^ mice and incubated with adenovirus (ad) expressing GFP to label epicardium (green), and either ad/βgalactosidase (βgal) or ad/Cre. Hearts were cultured ex vivo in TGFβ2 [2 ng/mL] and PDGF-BB [20 ng/mL] to induce EMT, and GFP^+^ epicardial cells were collected using FACS. GFP^−^ cells were collected as non-EPDCs. cTNT, cardiac troponin T (red), and DAPI staining were utilized to visualize nuclei (blue). Scale bar, 50 μm. Refer to Supplementary Fig. 8e for FACS sequential gating and enrichment of epicardial cells. **b** Principal component analysis (PCA) of GFP^−^ non-EPDCs and GFP^+^ EPDCs from control, *Mrtf-a*;*Mrtf-b* double knockout (MRTF^epiDKO^) or *Srf* knockout (SRF^epiKO^) mice. **c** Analysis of differentially expressed genes (DEGs) reveals biological processes that are enriched between MRTF^epiDKO^ and control EPDCs. GO-term enrichment significance was determined using Fisher exact test. **d** Heat map highlighting the relative expression of genes involved in axon guidance signaling in EPDCs of the indicated genotype. Relative (Rel.) expression represents the scaled expression of rlog normalized data. **e** Expression of genes encoding axon guidance cues was validated by qRT-PCR. Values are represented as a fold change in expression relative to non-EPDCs (dashed line at 1). *n* represents samples acquired from independent embryos, which were analyzed in 1 experiment. *n* = 3 non-EPDCs, *n* = 4 for *Efna5*, *Sema3d*, *Slit3*, *Wnt5a,* and *n* = 3 for Slit2 in Control EPDCs; *n* = 3 MRTF^epiDKO^ EPDCs were analyzed. Data are presented as mean values ± SEM. Statistical significance was determined by a two-sample unpaired Student’s *t*-test. TGFβ2 transforming growth factor beta-2, PDGF-BB platelet-derived growth factor BB, m-tdTom membrane tdTomato, m-eGFP membrane-enhanced green fluorescent protein, TAM tamoxifen.

Myocardin-related transcription factors (MRTFs) are mechanosensitive transcriptional co-activators of serum response factor (SRF) that facilitate induction of cell contractility and motility gene programs^33^. We previously reported that epicardium-specific Cre-mediated deletion of *Mrtf-a* and *Mrtf-b* (MRTF^epiDKO^) or *Srf* (SRF^epiKO^) during development impedes epicardial EMT, precipitating epicardium detachment and defects in coronary plexus formation and EC integrity^7^. This phenotype was attributed to a depletion of microvascular pericytes, which partially phenocopied the SRF-dependent emergence of mural cells from the epicardium^19^. In order to evaluate whether epicardial EMT controls the angiogenic programs identified by scRNA-seq, we used FACS to isolate GFP^−^ non-EPDCs (enriched in myocytes), and GFP^+^ EPDCs from wild-type (control) and mutant embryos for RNA-sequencing (Fig. 4a and Supplementary Fig. 8a–d). EPDCs obtained from MRTF^epiDKO^ and SRF^epiKO^ mice are transcriptionally indistinguishable based on principal component analysis (PCA), but diverge from control EPDCs stemming from the dysregulation of 2,518 genes (Fig. 4b, c and Supplementary Fig. 9a, b). MRTF^epiDKO^ EPDCs display a significant reduction in genes associated with biological processes such as cell migration (*Mylk*, *Vin*), ECM production (*Col1a2*, *Col3a1*), and mesenchymal cell differentiation (*Pdgfra*, *Acta2*, *Tagln*) (Supplementary Fig. 9c–i). Surprisingly, MRTF^epiDKO^ EPDCs also exhibit a significant downregulation of genes associated with paracrine regulation of chemotaxis and axon guidance (Supplementary Fig. 9c, j). Indeed, axon guidance was the most significantly dysregulated biological process in MRTF^epiDKO^ EPDCs, represented by secreted ligands such as *Efna5*, *Sema3d*, *Slit2*, *Slit3*, and *Wnt5a* (Fig. 4c, d). qRT-PCR confirmed genes encoding select guidance cues are enriched in EPDCs, and significantly downregulated upon MRTF deletion (Fig. 4e). RNAscope FISH also confirmed the reduction of *Sema3d* and *Slit2* in EPDCs of MRTF^epiDKO^ hearts, compared to controls (Supplementary Fig. 10a–c). These findings corroborated our scRNA-seq results revealing the restriction of vascular guidance cues to distinct populations of epicardium-derived cells, and provided evidence that EMT contributes to the expression and localization of these factors.

### EMT regulates the expression of genes encoding vascular guidance cues

**Fig. 5:**
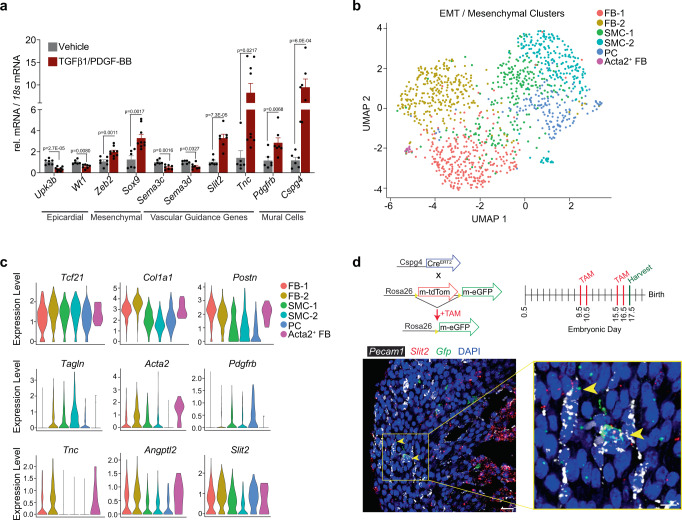
Vascular guidance cues are differentially expressed upon in vitro induction of EMT. **a** Primary epicardial cells were cultured ± TGFβ1 [10 ng/mL] and PDGF-BB [20 ng/mL] to induce EMT. Relative expression of genes encoding axon guidance cues was evaluated by qRT-PCR, along with markers of epicardial, mesenchymal, and mural cells. *n* represents samples isolated from independent embryos, which were analyzed over 2 experiments. Vehicle *n* = 6 for *Sox9*, *Sema3c*, *Tnc* and *n* = 7 for *Upk3b*, *Wt1*, *Zeb2*, *Sema3d*, *Slit2*, *Pdgfrb*, *Cspg4*; and TGFβ1/PDGF-BB *n* = 7 for *Wt1*, *Sema3c*, *Sema3d*, *Slit2*, *Pdgfrb*, *Cspg4* and *n* = 9 for *Upk3b*, *Zeb2*, *Sox9*, *Tnc*. Data are presented as mean values ± SEM. Statistical significance was determined by a two-sample unpaired Student’s *t*-test. **b** Seurat was used to re-cluster cells undergoing EMT (cluster 5) and mesenchymal cells (clusters 6/7) defined in Fig. 1f. **c** Violin plots representing select genes associated with fibroblast or mural cell identity. Expression level represents log normalized expression. **d** Experimental strategy for lineage tracing vascular mural cells in Cspg4^CreERT2^;R26^mTmG^ embryos. FISH was performed on hearts with probes directed against *Gfp* (green) and *Slit2* (red) as demonstrated in low (left) and high (right) magnification images and relative to *Pecam1*^+^ (platelet endothelial cell adhesion molecule-1, white) endothelial cells. Yellow arrowheads, *Slit2* expression in *Cspg4* lineage-derived cells. Scale bar, 25 μm (left image) and 10 μm (zoom right image). DAPI staining was utilized to visualize nuclei (blue). Immunostaining was repeated independently 3 times with similar results. EMT epithelial-to-mesenchymal transition, TAM tamoxifen, FB fibroblast, SMC smooth muscle cell, PC pericyte.

In order to further examine the effect of EMT on vascular guidance gene expression, we treated primary epicardial cells isolated from E11.5 embryos with TGFβ1 and PDGF-BB, which resulted in the downregulation of epicardial/mesothelial genes and upregulation of EMT-associated and mesenchymal genes (Fig. 5a)^34^. *Sema3c* and *Sema3d* were both significantly suppressed upon induction of epicardial EMT, whereas *Tnc and Slit2* were upregulated (Fig. 5a). These gene expression changes are consistent with their in vivo distribution within mesothelial cells and mesenchymal cells, respectively. We also found evidence that EMT induces the mural cell phenotype based on the expression of pericyte marker genes *Pdgfrb* and *Cspg4* (Fig. 5a). We, therefore, re-evaluated EPDC populations 5, 6, and 7 (from Fig. 1) to establish the identity of *Wt1*-lineage mesenchymal cells and define the source of epicardium-derived guidance cues (Fig. 5b). We were able to identify fibroblasts (Fb-1, Fb-2, Acta2^+^ Fb) based on increased expression of *Col1a1*, *Postn*, and *Tnc*; smooth muscle cells (SMC-1, SMC-2) based on increased expression *Tagln*; and pericytes (PC) based on increased expression of *Pdgfrb* (Fig. 5c). *Slit2* and *Angptl2* are enriched in FB1 and FB2, and *Slit2* is especially pronounced in pericytes (Fig. 5c). The *Cspg4*^*CreERT2*^ mouse line has been used to lineage trace vascular mural cells, including pericytes^35^. FISH using probes against *Gfp* and *Slit2* on heart sections obtained from Cspg4^CreERT2^;R26R^mTmG^ embryos obtained at E17.5 revealed *Slit2* transcripts within some *Cspg4* lineage-derived mural cells (Fig. 5d). Collectively, these data describe a paradigm whereby epicardial EMT is responsible for the restriction of individual chemotactic cues to distinct epicardium-derived lineages, including coronary mural cells, which may represent a vascular guidance cell reminiscent of the guidepost neuron^16^.

### Single-cell transcriptomics defines the EC response to epicardial dysfunction

**Fig. 6:**
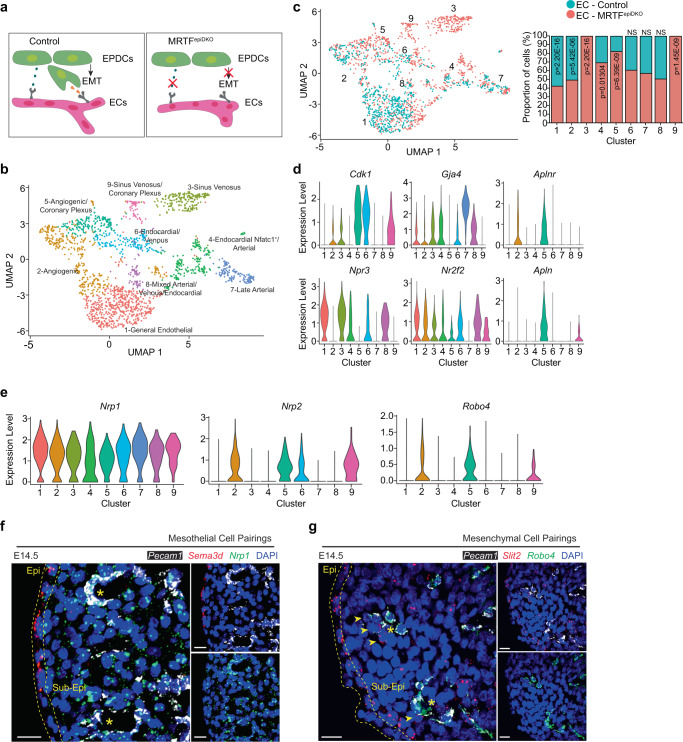
Disruption of epicardial EMT alters intercellular cross-talk between the epicardium and coronary vasculature. **a** Schematic of vasculature development representing disruption of intercellular signaling hypothesized in *Mrtf-a*;*Mrtf-b* double knockout (MRTF^epiDKO^) hearts. **b**, **c** UMAP representation of single-cell transcriptomes of embryonic day (E)14.5 endothelial cells (ECs) represented by **b** cell identities and **c** genotype with the proportion of each cell cluster (%) contributing to either the Control or MRTF^epiDKO^ genotype is graphed on the right. A two-sample Student’s *t*-test was performed to determine the significance of the proportion of cells in single-cell clusters. Refer to Supplementary Fig. 11b, c for FACS sequential gating and enrichment of ECs. **d** Violin plots showing expression of select genes associated with EC identity. **e** Violin plots showing expression of select receptors for Sema3d and Slit2 ligands in the 9 EC clusters. Expression level in **d** and **e** represents log normalized expression. **f**, **g** Expression of select ligand–receptor pairs within epicardial cells and Pecam1^+^ ECs (platelet endothelial cell adhesion molecule-1, white) at E14.5 visualized by FISH. **f** Mesothelial ligand *Sema3d* (red) is observed on the epicardial surface and *Nrp1* (green) expression is in ECs. **g** The mesenchymal enriched ligand *Slit2* (red) is expressed in interstitial epicardial cells, often associated with *Robo4*-positive (green) ECs. Yellow asterisks, developing coronary vessels. Yellow arrowheads, *Slit2* expressing cells. Dashed lines signify the epicardial border. Scale bars, 20 μm. DAPI staining was utilized to visualize nuclei (blue). Immunostaining was repeated independently 3 times with similar results. NS not-significant, EMT epithelial-to-mesenchymal transition, Epi epicardium, Sub-Epi sub-epicardium.

Coronary EC re-specification into arterial and venous fates occurs at around E14.5^1,2^. In order to interrogate the impact of epicardial EMT on individual ECs, we isolated CD31^+^/CD45^−^ cells from MRTF^epiDKO^ and Control hearts at E14.5 by FACS followed by single-cell capture and scRNA-seq using the 10× Genomics platform (Fig. 6a and Supplementary Figs. 11a–c and 12a, b). CCA defined 9 unique EC populations that were enriched in *Pecam1* (Supplementary Fig. 13a, b), and alleviated concerns of batch effects based on genotype and cell cycle analysis (Supplementary Fig. 13c, d; Supplementary Dataset 3 and 4). Since cell cycle is reported to underlie transcriptional differences during EC differentiation^9,36^, we performed unbiased clustering without regression of cell cycle to allow for identification of EC phenotypes that emerge upon disruption of epicardial EMT (Fig. 6b and Supplementary Fig. 13e). This analysis defined 9 unique cell populations consisting of ECs categorized as sinus venosus (SV), coronary plexus, angiogenic, venous, arterial, endocardial, and general endothelial (Fig. 6b). UMAP plots of filtered and typed ECs showed that cell clusters C3-C5 and C9 were significantly enriched with MRTF^epiDKO^ ECs (Fig. 6b, c and Supplementary Fig. 13f, g). Violin gene expression plots defined MRTF^epiDKO^-enriched sub-populations of SV and coronary plexus ECs that exhibit differential expression of *Aplnr*, *Apln,* and the proliferative marker *Cdk1*^9,37^ (Fig. 6d). Angiogenic genes *Sparcl1* and *Cd47*^38^ and pre-arterial genes *Dll4* and *Sox17*^9^ were also enriched in MRTF^epiDKO^ dominated clusters (Supplementary Fig. 14a–c). However, genes associated with the endocardium (*Npr3*, *Nfatc1*)^8,39^, venous (*Nr2f2*)^9^ or mature arterial (*Gja4*, *Gja5*, *Fbln5*)^9,38^ were similarly expressed in UMAP clusters of Control and MRTF^epiDKO^ ECs (Fig. 6d and Supplementary Fig. 14c, d). Overall, these data reveal that disruption of epicardial EMT leads to the emergence of a population of ECs that exhibit an immature vascular cell phenotype.

### Identification of potential receptor–ligand pairings between the endothelium and epicardium

Coordination of cell-to-cell signaling is critical to building vascular networks and underlies the acquisition of arterial and venous cell identity. To characterize the intercellular signaling between EPDCs and coronary ECs that influence developmental angiogenesis, we constructed a receptor–ligand visualization by matching EC-expressed receptors to epicardium-derived ligands identified by scRNA-seq, indicating whether a particular ligand is enriched within epicardium-derived mesothelial or mesenchymal cells (Supplementary Fig. 15a). We then identified epicardium-derived vascular guidance genes that are influenced by epicardial EMT by cross-referencing to our bulk RNA-sequencing of MRTF^epiDKO^ EPDCs (Supplementary Fig. 16a and Supplementary Dataset 5). We identified 99 genes encoding ligands that are dysregulated upon *Mrtf* deletion, potentially impacting pathways related to proliferation, growth factor signaling, non-canonical Wnt signaling, ECM–receptor interactions, and axon guidance (Supplementary Fig. 16a–c); a total of 52 ligands that are normally restricted to the mesothelium or mesenchyme in control mice are disrupted (Supplementary Fig. 17a–c). Notably, 34 genes encoding receptors that are detected in ECs were significantly altered following *Mrtf* deletion within the epicardium (Supplementary Fig. 18), which led to the potential disruption of 87 receptor–ligand pairs (Supplementary Fig. 15b and Supplementary Dataset 6).

We next re-evaluated the single EC transcriptome and conducted RNA FISH to evaluate the distribution of key cell surface receptors within the fetal heart, focusing on candidate receptors for Sema3d (mesothelium-derived) and Slit2 (guidepost cell-derived). We detected the semaphorin co-receptor *Nrp1* within all EC clusters (Fig. 6e). RNA FISH confirmed the widespread expression of *Nrp1* within a majority of *Pecam1*-expressing ECs, while *Sema3d* is restricted to mesothelial cells on the cardiac surface (Fig. 6f and Supplementary Figs. 19, 20a). In contrast, *Nrp2* and *Robo4* are primarily restricted to angiogenic and coronary plexus EC within clusters 2, 5, and 9 (Fig. 6e). FISH confirmed the expression of *Robo4* within a distinct population *Pecam1*^+^ ECs that often reside in close proximity to *Slit2*^+^ cells (Fig. 6g and Supplementary Figs. 19, 20b). These data reveal the potential for complex intercellular cross-talk between EPDC and EC that may influence coronary angiogenesis.

### Disruption of epicardial EMT alters EC developmental trajectory

**Fig. 7:**
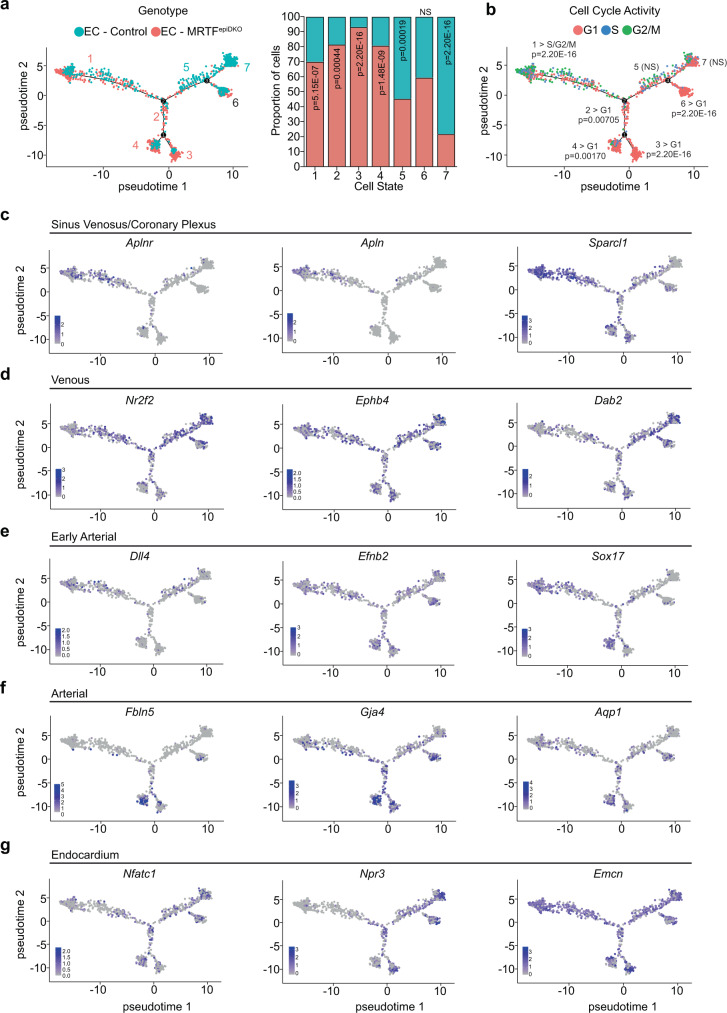
Disruption of epicardial EMT alters arteriovenous differentiation and maturation. **a**, **b** Monocle-generated pseudotime trajectory of ECs from control and *Mrtf-a*;*Mrtf-b* double knockout (MRTF^epiDKO^) hearts. Developmental trajectories represented by **a** genotype with the proportion (%) of control or MRTF^epiDKO^ ECs represented in each cell state (right) and **b** cell cycle activity with a representation of cells in the G1, S, or G2/M phase of the cell cycle. A two-sample Student’s t-test was performed to determine significance of the proportion of cells in single-cell clusters presented in **a** and **b**. **c**–**g** Pseudotime feature plots representing the expression of genes related to the **c** sinus venosus/coronary plexus, **d** venous, **e** early arterial, **f** late arterial, and **g** endocardial cell identity. The scale represents relative (Rel.) expression. NS not-significant, EMT epithelial-to-mesenchymal transition.

To define how EC developmental trajectory is altered upon disruption of epicardium-derived paracrine signaling, we ordered ECs obtained from control and MRTF^epiDKO^ mice at E14.5 in pseudotime using Monocle (Supplementary Fig. 21a and Supplementary Dataset 7). While each pseudotime state is composed of ECs obtained from animals of both genotypes, states 5 and 7 are enriched in ECs from control mice, and states 1–4 are enriched in MRTF^epiDKO^ ECs (Fig. 7a and Supplementary Fig. 21b, c). Cell state also correlates with cell cycle activity (Fig. 7b), consistent with reports that EC maturation coincides with reduced proliferation^9,36^. Pseudotime originates in state 1 with SV and angiogenic coronary progenitors, marked by the expression of *Aplnr*, *Apln*, and *Sparcl1*, and an over-abundance of cells in the G2/M and S phases of the cell cycle (Fig. 7b, c). ECs obtained from control hearts primarily followed a trajectory from progenitor state 1 through a mixed venous/primed arterial EC state 5 (represented by *Nr2f2, Ephb4, Dab2*) towards terminal states 6 and 7. State 6 displays immature arterial-like characteristics, with relatively lower levels of the venous markers *Nr2f2* and *Ephb4*, elevated levels of the early arterial gene *Efnb2,* and intermediate levels of the mature arterial gene marker *Gja4* (Fig. 7d–f and Supplementary Fig. 22a, b). In contrast, terminal state 7 is represented by cells with more venous-like characteristics, expressing higher levels of *Nr2f2*, *Ephb4,* and *Dab2* (Fig. 7d and Supplementary Fig. 22a, b). The start of pseudotime is enriched in ECs obtained from MRTF^epiDKO^ hearts, which transition through a unique developmental trajectory (states 2–4), characterized by ECs that have exited the cell cycle (Fig. 7a, b). State 2 represents a transient EC phenotype leading towards state 4, which is defined by early (*Efnb2, Sox17*) and mature (*Fbln5*, *Gja4,* and *Aqp1)* arterial markers (Fig. 7e, f and Supplementary Fig. 22a, b). Cell state 3 is composed nearly entirely of ECs from MRTF^epiDKO^ hearts that have exited the cell cycle and express intermediate levels of angiogenic and arterial genes as well and endocardial markers (Fig. 7f, g and Supplementary Fig. 22a, b). Endocardial gene markers *Nfatc1* and *Npr3* are most enriched in terminal states 3, 6, and 7, whereas *Emcn* was detected broadly across cells ordered in pseudotime (Fig. 7g and Supplementary Fig. 22a, b). Collectively, ECs from MRTF^epiDKO^ hearts lack obvious venous identity markers and show a bias towards immature arterial fate, indicating epicardial EMT-dependent EPDC-EC signaling may directly impact EC maturation.

### Disruption of epicardial EMT alters EC maturation and localization

**Fig. 8:**
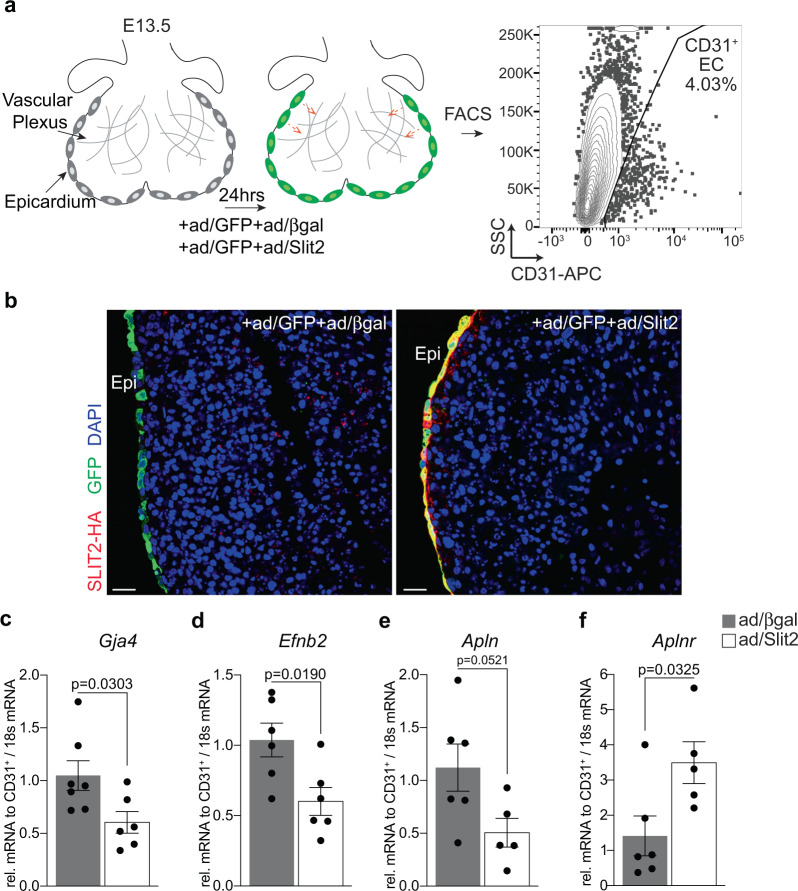
SLIT2 expression in the epicardium inhibits artery specification. **a** Schematic of experimental design to isolate ECs following adenovirus infection of the epicardium. Hearts were extracted at embryonic day (E) 13.5 and infected with adenovirus (ad) to express β-galactosidase (ad/β-gal) or SLIT2-HA (ad/Slit2, red). Ad expressing GFP was added to hearts to confirm the specificity of infection to cells of the epicardium (green). Following 24-h, hearts were digested and subjected to FACS to acquire CD31 expressing ECs. Refer to Supplementary Fig. 23d, e for FACS sequential gating and enrichment of ECs. **b** Representative images of embryonic hearts following infection with adenoviruses. SLIT2 protein expression was detected in the epicardium using an anti-HA antibody. Scale bar, 20 μm. DAPI staining was utilized to visualize nuclei (blue). Immunostaining was repeated independently 3 times with similar results. **c**–**f** Gene expression represented as fold change relative to CD31^+^ cells acquired from ad/βgal-treated hearts. *n* represents samples acquired from independent embryos. ad/β-gal *n* = 6 for *Efnb2*, *Apln*, *Aplnr* and *n* = 7 for *Gja4;* and ad/Slit2 *n* = 5 for *Apln* and *Aplnr* and *n* = 6 for *Gja4* and *Efnb2*. Data are presented as mean values ± SEM. Statistical significance was determined by a two-sample unpaired student’s *t*-test.

Coronary vessel maturation requires the re-specification of immature ECs towards arterial fate in deeper myocardium, and venous fate in sub-epicardial myocardium. To begin investigating the potential impact of guidepost cell-derived ligands on coronary EC differentiation, we used an adenoviral-vector to express Slit2 in E13.5 hearts cultured ex vivo (Fig. 8a and Supplementary Fig. 23a). This method allowed for specific targeting of Slit2, or GFP control, to the epicardium (Fig. 8b). Following 24 h of infection, CD31^+^ ECs were isolated from hearts using FACS (Fig. 8a), and EC differentiation and maturation markers were evaluated by qRT-PCR. Slit2 overexpression led to a reduction in the expression of arterial markers *Gja4*, *Efnb2,* and *Apln*, and increased expression of the venous/angiogenic endothelium marker *Aplnr*, although key venous markers (*Nr2f2* and *Ephb4*) were unchanged^9,38,40,41^ (Fig. 8c–f and Supplementary 23b, c). Our data are consistent with reports of Slit2 regulating angiogenesis^42,43^, and suggest a common mechanism may guide vascular pathfinding and arteriovenous fate specification.

**Fig. 9:**
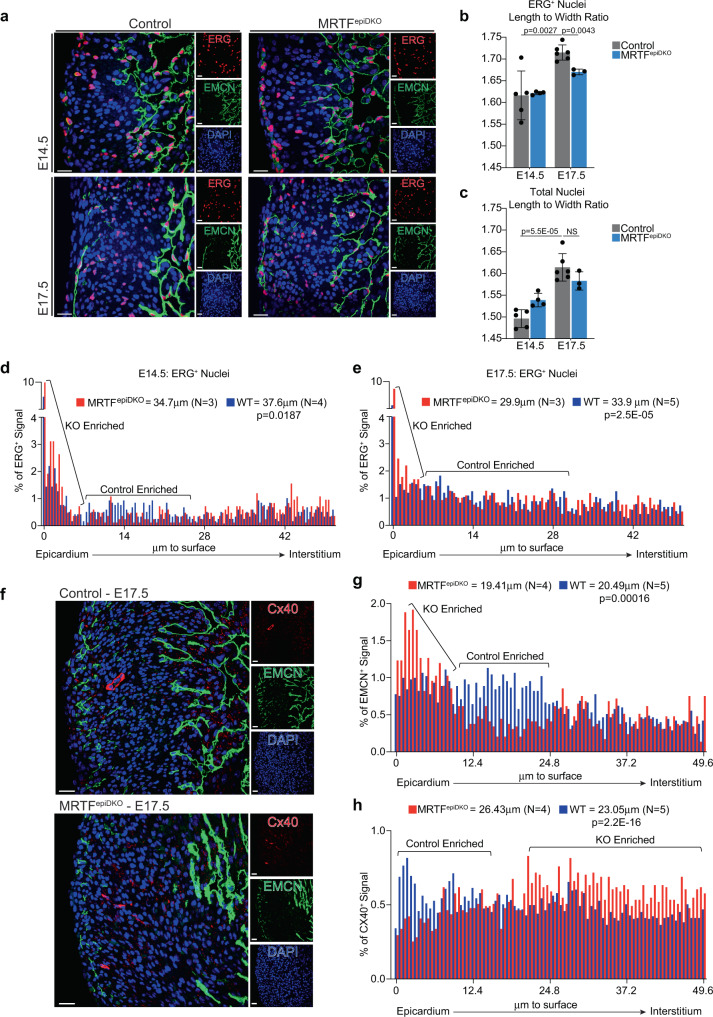
Epicardial dysfunction alters endothelial cell localization and maturation. **a** Immunofluorescence staining of sections from hearts isolated at embryonic stage (E)14.5 and E17.5. Antibodies are directed against ERG (red, pan-EC) and EMCN (green, venous, and endocardial EC). Scale bar, 20 μm. **b** Quantitation of the length-to-width ratio of ERG^+^ nuclei (top) or **c** total nuclei (bottom). *n* represent samples acquired from independent embryos. At E14.5, *n* = 5 Control hearts and *n* = 4 *Mrtf-a*;*Mrtf-b* double knockout (MRTF^epiDKO^) hearts. At E17.5, *n* = 6 Control hearts and *n* = 3 MRTF^epiDKO^ hearts. For each heart, at least three fields of view were assessed. Data are presented as mean values ± SEM. Statistical significance was determined by a two-sample unpaired Student’s *t*-test. **d**, **e** Quantitation of ERG^+^ nuclei localization, reported as a percentage of cells within a particular bin representing the distance from the epicardial surface of the heart at **d** E14.5 and **e** E17.5. **f** Immunofluorescence staining of sections from hearts isolated at E17.5 with antibodies directed against EMCN (green) and Cx40 (red, arterial). Scale bar, 25 μm. **g**, **h** Quantitation of **g** EMCN^+^ cell localization and **h** Cx40^+^ cell localization, reported as a percentage of cells within a particular bin representing the distance from the epicardial surface of the heart. For localization experiments, *n* represents data acquired from independent embryos, which was analyzed in 1 experiment. For ERG + nucleus localization *n* = 4 Control hearts and *n* = 3 MRTF^epiDKO^ hearts at E14.5; and *n* = 5 Control hearts and *n* = 4 MRTF^epiDKO^ hearts at E17.5. For Cx40 and Emcn localization, *n* = 5 Control hearts and *n* = 4 MRTF^epiDKO^ hearts at E17.5. Significant accumulation of ECs in particular regions of the heart are marked by brackets that indicate the over-represented genotype. For each heart, at least three fields of view were assessed. DAPI staining was utilized to visualize nuclei (blue). For data in **d**, **e**, **g**, **h** statistical significance was determined by a two-tailed Mann–Whitney test. NS not-significant, WT wild-type, KO knockout.

EC maturation is also characterized by cell polarization, induced by the connection of the nascent vasculature to arterial blood flow, which increases hemodynamic shear stress and supports EC alignment and migration against flow^44^. In order to evaluate EC maturation and polarization, we immuno-stained sections from control and MRTF^epiDKO^ hearts obtained at E14.5 and E17.5 with antibodies directed against EMCN and ERG, a pan-EC ETS-family transcription factor^45^. The length-to-width ratio of ERG^+^ nuclei was quantified as an indicator of cell polarity, revealing considerable elongation between E14.5 and E17.5 in control hearts, an alteration that was not observed in ERG^+^ nuclei of MRTF^epiDKO^ hearts (Fig. 9a–c). Quantification of ERG^+^ nucleus localization also revealed an inappropriate accumulation of ECs near the epicardial surface in MRTF^epiDKO^ hearts at E14.5 and E17.5 (Fig. 9a, d, e and Supplementary Fig. 24a, b). To further interrogate the distribution of mature ECs, immunostaining was performed to visualize venous EC (EMCN, green) and arterial EC (Cx40, red), which display sub-epicardial and mid-myocardial localization in control E17.5 hearts. In contrast, EMCN^+^ venous cells displayed an abnormal accumulation near the epicardial surface in MRTF^epiDKO^ hearts (Fig. 9f, g; Supplementary Fig. 25). We also often found Cx40^+^ arterial EC as discontinuous patches of cells without a discernable lumen, which resided deeper within the myocardium of MRTF^epiDKO^ hearts (Fig. 9f, h; Supplementary Fig. 25). Collectively, these findings reveal a contribution of epicardium-derived pathfinding cues to EC localization and AV specification.

## Discussion

In summary, our data establish epicardial EMT as a driving force in the generation of distinct expression domains of vascular patterning cues characterized by: (1) Mesothelial cells on the surface of the heart expressing angiogenic chemokines such as Sema3d; and (2) Epicardium-derived mesenchymal cells that express chemokines such as Slit2 and Angptl2. Our data also reveal the coordinated regulation of coronary EC localization and AV specification by epicardium-derived vascular patterning cues.

We previously reported that deletion of MRTFs in the epicardium prevents EMT, and inhibits coronary plexus formation^7^. The current transcriptome analyses further establish the epicardium as an important source of vascular guidance cues in the embryo, which is disrupted in MRTF mutant mice. Here, we define the specific role of epicardial EMT in establishing the spatial pattern of vascular cues that control EC patterning. We found that EMT induces the expression of secreted ligands that are found in epicardium-derived mesenchyme, while silencing those ligands that are restricted to the mesothelium. *Slit2* is especially induced upon epicardial EMT, and localizes to a minor population of epicardium-derived fibroblasts and pericytes that we term vascular “guidepost cells”. This population is reminiscent of the guidepost neuron in axon patterning, which provides non-continuous landmarks that act as “stepping stones” for growing axons^16^. While the regulation of vascular guidance molecules seems largely dependent on EMT, reduction of the mesothelium-restricted *Sema3d* in MRTF mutant mice suggests general epicardial dysfunction, supported by the suppression of canonical epicardial genes *Aldh1a2*, *Tbx18*, *Tcf21,* and *Wt1*^15,46,47^.

Prior studies have revealed the importance of individual factors such as Sema3d and Slit2 in patterning of coronary venous cells and supporting cardiomyocyte cytokinesis^48,49^. Here, we found *Slit2*^*+*^ guidepost cells in close proximity to *Robo4*^+^ ECs in the sub-epicardium; thus, Slit2-Robo4 interactions are positioned to control angiogenesis and vascular stability, as described in other contexts^37,43,50,51^. Indeed, our study found that overexpression of Slit2 suppressed the arterial EC phenotype in ex vivo heart culture, based on the expression of arterial (*Gja4* and *Efnb2)* and angiogenic venous markers (*Aplnr*). This result is consistent with the accumulation of ECs that exhibit an immature arterial phenotype upon suppression of *Slit2* expression in MRTF^epiDKO^ hearts. However, Cx40^+^ arterial ECs become mislocalized and fail to consistently form lumens in MRTF^epiDKO^ embryos at E17.5, revealing a defect in EC maturation. Evidence for improper arterial cell differentiation upon epicardial disruption is consistent with the retention of a sinus venosus and coronary plexus EC phenotype, represented by the expression of *Aplnr*, *Apln*, *Vegfa*, *Vegfc*, *Cd47*. Of note, AV specification is in part regulated by COUP-TFII (also known as Nr2f2), which inhibits Notch activity in ECs and blocks differentiation into arterial cells^52^. However, *Nr2f2* expression was not altered by Slit2 overexpression in heart cultures; therefore, it appears the impact of Slit2 on EC identity is only partial, suggesting additional factors are required for normal EC maturation. Therefore, future studies should interrogate the combinatorial paracrine code that governs normal AV specification.

Importantly, acquired and developmental vascular abnormalities underlie many human diseases, including stroke and heart disease. For example, coronary artery disease (CAD) disrupts the vascular network that supplies the heart with oxygen and nutrients. Although environmental factors including a sedentary lifestyle and a high-fat diet contribute to CAD progression, accumulating evidence suggests a considerable genetic component to disease risk^53^. One of the strongest genetic risk factors for CAD is the *Tcf21* gene, which is highly expressed in the fetal epicardium and is essential for normal cardiac fibroblast and coronary vessel formation^46,47^. Therefore, a better understanding of epicardium-directed coronary vessel formation in development may provide insight into CAD mechanisms.

Regenerative therapeutic strategies for cardiac repair include approaches to promote cardiomyocyte proliferation^54^ and sympathetic innervation^55,56^; however, strategies to stimulate re-vascularization such as through enhancing coronary collateralization must complement new muscle formation. Single-cell transcriptomic analysis has identified populations of neovasculogenic ECs that emerge following MI^57^, and limited angiogenesis of the injured adult heart is reported to occur through the activation of developmental angiogenic programs^58,59^. Indeed, the epicardium induces a fetal gene program after myocardial infarction that includes a paracrine signature^60,61^. Unfortunately, the reactivation of endogenous angiogenic programs in the adult heart is insufficient to support meaningful collateralization of ischemic tissue. Therefore, our study describing the paracrine cues underlying developmental coronary angiogenesis may provide a framework to establish re-vascularization strategies for cardiac regenerative medicine.

## Methods

### Animal models

All animal experiments were conducted in accordance with the ethical regulations for testing and research and approved by the University Committee on Animal Resources at the University of Rochester (UCAR-2011-026E). C67BL/6J mice were purchased from The Jackson Laboratory (stock number 000664) and all mouse lines were maintained on the C57BL/6J background. Rosa26^mTmG/mTmG^ mice were purchased from The Jackson Laboratory (stock number 007576). Rosa26^tdTomato^ mice were purchased from The Jackson Laboratory (stock number 007909). The Wt1^CreERT2^ mouse strain expresses the CreERT2 fusion protein in the presence of tamoxifen and under the control of *Wt1* promoter. Wt1^CreERT2^ mice were used to efficiently label the epicardium and its derivatives and as previously described^6^ and were purchased from The Jackson Laboratory (stock number 010912). Cspg4^CreERT2^ mice express the tamoxifen-inducible Cre-recombinase under the control of *Cspg4* promoter. Cspg4^CreERT2^ mice were used to label cardiac pericytes during embryonic development and is a validated model to label *Cspg4* expressing cells^35^ and were purchased from The Jackson Laboratory (stock number 008538). Mrtfa^−/−^ and Mrtfb^flox/flox^ mice were previously described^7^ and were gifts from Dr. Eric Olson (UT Southwestern, Dallas, TX, USA). The Srf^flox/flox^ mice were previously described^62^ and were a gift from Dr. Joseph Miano (Augusta University, Augusta, GA, USA).

Timed pregnancies were determined after placing one male with up to two females in a single cage in the late afternoon. The next morning, a confirmed plug was termed as embryonic day (E)0.5. In order to induce Cre-based recombination, 4-Hydroxytamoxifen (4-OHT, Millipore Sigma H6278) was dissolved in sunflower seed oil from helianthus annus (Millipore Sigma S5007) at a final concentration of 10 mg/mL with 10% ethanol. 4-OHT was administered by oral gavage at 75 mg/kg to pregnant dams.

4-OHT administration and dissection schedules for individual experiments were: (1) The breeding strategy to generate developmentally staged embryos for single-cell RNA-sequencing of epicardial cells and isolation of hearts for immunostaining and in situ hybridization assays: Wt1^CreERT2/+^ males were crossed to Rosa^mTmG/mTmG^ females. 4-OHT was administered at E9.5 and E10.5 and embryos were isolated at E12.5 and E16.5. (2) The breeding strategy to generate developmentally staged embryos for gene expression analysis in epicardial cells: Wt1^CreERT2/+^ males to Rosa^mTmG/mTmG^ females or Rosa^tdTomato/tdTomato^ females. 4-OHT was administered at E9.5 and E10.5 and embryos were isolated at E12.5, E14.5, and E16.5. (3) The breeding strategy to generate developmentally staged embryos for the analysis of cardiac pericytes by in situ hybridization assays: Cspg4^CreERT2/+^ males were crossed to Rosa^mTmG/mTmG^ females. 4-OHT was administered at E9.5/E10.5 and E15.5/E16.5 and embryos were isolated at E17.5. (4) The breeding strategy to generate developmentally staged embryos for single-cell RNA-sequencing of endothelial cells and isolation of hearts for immunostaining and in situ hybridization assays: Wt1^CreERT2/+^ males were crossed to C57BL/6J mice to generate Control embryos. Wt1^CreERT2/+^; Mrtf-a^−/−^; Mrtf-b^flox/flox^ males were crossed to Mrtf-a^−/−^; Mrtf-b^flox/flox^ females to generate MRTF^epiDKO^ embryos. 4-OHT was administered at E9.5 and E10.5 and embryos were isolated at E14.5 and E17.5. (5) The breeding strategy to generate developmentally staged embryos for isolation of Control and MRTF mutant epicardial cells for bulk RNA-sequencing and gene expression studies: Mrtf-a^−/−^; Mrtf-b^flox/flox^ males were crossed to Mrtf-a^−/−^; Mrtf-b^flox/flox^ to generate Mrtf-a^−/−^; Mrtf-b^flox/flox^ embryos. SRF^flox/flox^ males were crossed to SRF^flox/flox^ females to generate SRF^flox/flox^ embryos. Embryos were dissected at E12.5 for heart culture and epicardium-derived cell labeling and gene deletion was conducted via adenoviral-vector mediated delivery of GFP and Cre-recombinase, as described below. (6) The breeding strategy to generate developmentally staged embryos for ex vivo expansion of primary epicardial cells and gene expression studies: C57BL/6J males were crossed to C57BL/6J females and embryos were isolated at E11.5. (7) The breeding strategy to generate developmentally staged embryos for isolation of endothelial cells following ex vivo heart culture and infection with adenoviruses: C57BL/6J males were crossed to C57BL/6J females and embryos were isolated at E13.5.

### Embryonic heart digestion protocol

Epicardium-derived cells (EPDCs) and endothelial cells (ECs) were isolated from developmentally staged hearts as defined above. On the day of isolation, pregnant dams were anesthetized with an intraperitoneal injection of 0.5 mL of ketamine-xylazine cocktail (13 mg/mL ketamine in 0.88 mg/mL xylazine in DPBS) followed by cervical dislocation. After the use of 70% ethanol to sterilize the abdominal area, an incision to enter and remove decidua away from the mesometrium was performed, and embryos were placed in pre-warmed HBSS (ThermoFisher Scientific, SH30031.02). After the removal of extraembryonic tissue and the yolk sac, the heart was removed from the embryo and placed in a cell culture well-containing culture media made up of M199 (ThermoFisher Scientific, SH3025301) supplemented with 10% FBS (Gemini Bio-Products, 100106) and 1% Penicillin/Streptomycin (Pen-Strep; ThermoFisher Scientific, SV30010). Digestion of embryonic hearts began by removing residual HBSS from wells and replacing media with a digestion solution containing 0.08% Collagenase IV (Millipore Sigma, C5138), 0.05% Trypsin Protease (ThermoFisher Scientific, SH30042.01), 1% chicken serum (Vector Laboratories, S-3000) diluted in pre-warmed HBSS before placing hearts in a 37 °C hybridization oven with gentle shaking for 5 min intervals. Following incubation, hearts were dissociated by gentle pipetting (3 times with a P1000 pipette) and undigested tissue was allowed to settle for 30 s. After settlement of tissue, media was collected and added to a separate tube containing horse serum (Vector Laboratories, S-2000) to neutralize digestion, and digested cells were then saved on ice. Digestion, pipetting, and collection of media were repeated 3-5 more times, and cells were then filtered through a 70 μm filter and centrifuged at 200 × *g* for 5 min at 4 °C. The resulting pellet was placed in 10% FBS in DMEM (without phenol red, ThermoFisher Scientific, SH30284.01) and saved on ice before performing fluorescence-activated cell sorting FACS using a BD FACS Aria II using a 100 μm nozzle (BD Biosciences). DAPI (4′,6-Diamidino-2-Phenylindole, Dihydrochloride) was added to cells immediately before sorting (0.5 μg/mL; ThermoFisher Scientific, D1306) to exclude dead cells. Cells were sorted directly into 1.5 mL Eppendorf tubes containing 0.5% bovine serum albumin (BSA, Millipore Sigma, A9647) in DPBS at 4 °C and immediately processed.

### Cell isolation of epicardial cells at E12.5 and E16.5 for scRNA-seq

EPDCs were collected from Wt1^CreERT2/+^; R26^mTmG/+^ embryos that were administered 4-OHT at E9.5 and E10.5 via pregnant dams. A total of 7 E12.5 staged hearts were pooled from 2 dams, and a total of 17 E16.5 staged hearts were pooled from 4 dams based on visual confirmation of green fluorescent protein (GFP) expression in the epicardium using a ZOE Fluorescent Cell Imager (Bio-Rad). Hearts negative for the expression of the Wt1^CreERT2^ allele, exhibited tdTomato fluorescence alone, and were either discarded or used as tdTomato positive fluorescence controls for flow cytometry. Developmentally staged C57BL/6J embryos were collected as non-fluorescence controls for flow cytometry. Additionally, genomic DNA was isolated from all embryos, and Wt1^CreERT2^; R26^mTmG/+^ positive embryos were confirmed by PCR genotyping using transgene-specific primers. Following the digestion protocol described, EPDCs were gated as single cells (based on FSC × SSC dimensions), DAPI negative, tdTomato negative, and GFP-positive. TdTomato positive cells were sorted for downstream gene expression analysis. EPDCs collected by FACS were immediately processed for single-cell capture, library preparation, and sequencing, as described below.

### Cell isolation of epicardial cells at E12.5, E14.5, and E16.5 for gene expression analysis

EPDCs were collected from both Wt1^CreERT2/+^; R26^mTmG/+^ and Wt1^CreERT2/+^; R26^tdTomato/+^ embryos that were administered 4-OHT at E9.5 and E10.5 via pregnant dams. Fluorescence was confirmed using the ZOE Fluorescent Cell Imager (Bio-Rad). Hearts negative for the expression of the Wt1^CreERT2^ allele, exhibited tdTomato fluorescence (R26^mTmG/+^) or were non-fluorescent (R26^tdTomato/+^) and were either discarded or used as fluorescence controls for flow cytometry. Following the digestion protocol described, EPDCs were gated as single cells (based on FSC × SSC dimensions), DAPI negative, tdTomato negative, and GFP-positive if the cross was to the R26^mTmG^ fluorescent reporter. If the R26^tdTomato^ fluorescent reporter was used, DAPI negative and tdTomato positive EPDCs were collected. EPDCs collected by FACS were then processed for RNA isolation prior to conducting quantitative RT-PCR.

### Cell isolation of endothelial cells at E14.5 for scRNA-seq

ECs were collected from Wt1^CreERT2/+^ (Control) and Wt1^CreERT2/+^; Mrtf-a^−/−^; Mrtf-b^flox/flox^ (MRTF^epiDKO^) mice after administration of 4-OHT at E9.5 and E10.5 via oral gavage of pregnant dams. A total of 10 Control hearts were pooled from 2 dams. A total of 7 MRTF^epiDKO^ hearts were pooled from 2 dams. Prior to digestion, hearts were placed in HBSS at 37 °C and 5% CO_2_ and genomic DNA from all embryos were subjected to genotyping to detect the Wt1^CreERT2/+^ allele within 2 h. Following confirmation of positive embryos, hearts were subjected to the digestion protocol described. After filtering and centrifuging cells, ECs were incubated with fluorescently conjugated antibodies directed against CD31-APC (dilution at 1:100; BD Biosciences 551262) and CD45-FITC (dilution at 1:50; BD Biosciences 553079) for 30 min in 0.5% BSA in DPBS on ice. After antibody labeling, cells were washed and centrifuged at 200 g for 5 min and placed in 10%FBS/DMEM buffer. ECs were gated as single cells that are DAPI negative, CD45-FITC negative, and CD31-APC positive. ECs collected by FACS were immediately processed for single-cell capture, library preparation, and sequencing.

### Ex vivo embryonic heart culture for isolation of endothelial cells following adenovirus infection

ECs were collected from C57BL/6 hearts that were extracted at E13.5 and placed in culture media (DMEM:M199 with 10% FBS and 1% Pen-Strep) containing adenovirus to express β-galactosidase (Vector Biolabs, 1080) or SLIT2-HA (Applied Biological Materials, 132844A) for 24 h at 37 °C and 5% CO_2_ and subjected to the digestion protocol described. This method primarily transduces surface epicardial cells with adenovirus. After filtering and centrifuging cells, ECs were incubated with fluorescently conjugated antibodies to select for vascular EC (CD31-APC; BD Biosciences 551262) for 30 min in 0.5% BSA in DPBS on ice. After antibody labeling, cells were washed and centrifuged at 200 × *g* for 5 min and placed in 10%FBS/DMEM buffer. ECs were gated as single cells that are DAPI negative and CD31-APC positive. ECs collected by FACS were immediately processed for RNA isolation prior to conducting quantitative RT-PCR.

### Ex vivo embryonic heart culture for isolation of epicardial cells for bulk RNA-sequencing

Hearts were collected from Srf ^flox/flox^ (for control EPDC, Srf-KO EPDCs, and non-EPDC) and Mrtf-a^−/−^; Mrtf-b^flox/flox^ (for Mrtf-dKO) embryos that were extracted at E12.5 and placed in culture media (M199 with 10% FBS and 1% Pen-Strep) containing TGF-β2 (2 ng/mL; R&D Systems) and PDGF-BB (20 ng/mL; R&D Systems) to induce epithelial-mesenchymal transition. All explants were transduced with adenovirus to express a green fluorescent protein (GFP, Vector Biolabs, 1060) on the epicardial surface. Control hearts were co-transduced with adenovirus expressing β-galactosidase (Vector Biolabs, 1080) while gene deletion was accomplished by co-transduction with adenovirus expressing Cre-recombinase (Vector Biolabs, 1045) to excise floxed alleles (all adenovirus treatments were at 1 × 10^6^ pfu/mL). Following 48 h of culture at 37 °C and 5% CO_2_, hearts were dissociated and EPDCs were isolated via FACS by gating for single cells, and separated as GFP negative (non-EPDCs) or GFP-positive (EPDCs) from each group and collected in 5 mL FACS tubes containing 0.5 mL HBSS supplemented with 10% FBS. Hearts not treated with ad-GFP were used as non-fluorescence gating controls during flow cytometry analysis. Sorted cells were then pelleted at 200 × *g* for 5 min at 4 °C. Total RNA was isolated using TRIzol Reagent (ThermoFisher Scientific, 15596018) per manufacturer’s instructions and cleaned up with column purification. RNA quality was evaluated using a bioanalyzer and prepared into NGS libraries for bulk RNA-sequencing or was used for conducting quantitative RT-PCR.

### Single library preparation and processing of single epicardial cells and endothelial cells

Single-cell libraries were generated from epicardial cells and endothelial cells acquired by FACS. Prior to capture using the 10× Genomics Chromium controller (10× Genomics), the number of cells was quantitated (TC20 Automated Cell Counter, Bio-Rad) and cell viability was assessed via the trypan blue exclusion test of cell viability. Only cell populations exhibiting greater than 80% viability were used. All cells were loaded in order to maximize the number of single cells acquired using the Chromium single Cell 3′ Reagent Kit. Libraries were prepared according to the manufacturer’s instructions using the Chromium Single Cell 3′ Library and Gel Bead Kit v.2 (10× Genomics). CellRanger v2.2.0 was used to demultiplex each capture, process base-call files to fastq format, and perform 3′ gene counting for each individual cell barcode with mouse reference data set (mm10, v 2.1.0).

### Single-cell transcriptome sequencing of epicardial cells

Cell filtering and cell-type annotation and clustering analysis: Quality control, identification of variable genes, principle component analysis, and non-linear reduction using UMAP were performed using Seurat (v3.0.0.9000 and R v3.5.1) for each individual time point separately. The integration function RunCCA was utilized to identify cell type-specific clusters without respect to developmental time. Cell-type annotations were identified based on significant cluster-specific marker genes and the Mouse Gene Atlas using Enrichr (enrichR_2.1). In order to understand the effect of developmental time, the Seurat (v3.0.0.9150) function merge() was used to combine the E12.5 and E16.5 captures to maintain the variation introduced by developmental time. Cell cycle scoring was performed and the variation introduced as a number of genes involved in mitochondrial transcription, and cell cycle phases S and G2/M were regressed out during data scaling. Data was visualized in UMAP space and clustered were defined using a resolution of 0.5.

Developmental trajectory and prediction of cell-fate determinants: The GetAssayData() function in Seurat (v3.0.0.9150) was used to extract the raw counts to construct the Monocle object. To construct the trajectory the default functions and parameters as suggested by Monocle (v2.10.1) were used along with the following deviations: the hypervariable genes defined using Seurat VariableFeatures() function were used as the ordering genes in Monocle, 8 principle components were used for further non-linear reduction using tSNE, and num_clusters was set to 5 in the clusterCells() Monocle function. The resulting Monocle trajectory was colored based on Monocle State, Pseudotime, developmental origin (E12.5 or E16.5), and Seurat clusters previously identified. Genes that are dynamically expressed at the one identified branchpoint were analyzed using the BEAM() function. The top 50 genes that are differentially expressed at the branchpoint were visualized using the plot_genes_branched_heatmap() function in Monocle.

Integration with Mouse Cell Atlas. Neonatal hearts from one-day-old pups were downloaded from the Mouse Cell Atlas (https://figshare.com/articles/MCA_DGE_Data/5435866) and re-analyzed using Seurat v3 following standard procedures previously outlined. Epicardial (E12.5 and E16.5) and neonatal-heart (1 day old) were integrated using the FindIntegegrationAnchors() and IntegrateData() functions using Seurat v3. Data were visualized in the 2-dimensional UMAP space. Marker genes were identified for the integrated clusters and Enrichr (enrichR_2.1) was used to identified significantly enriched Biological Processes (Gene Ontology 2018).

### Single-cell transcriptome sequencing of endothelial cells

Cell filtering, cell-type clustering analysis, and creation of cellular trajectories: Seurat (3.0.2) was used to filter low-quality cells, score the cells by the cell cycle, and integrate the E14.5 MRTF^epiDKO^ and Control datasets using the merge function. Cells were clustered using the first 36 dimensions of PCA to the resolution of 0.7 and visualized using UMAP. Monocle (2.10.1) was used to infer cellular trajectory after the removal of cell cycle-related genes. The determined cell states were used to determine cell state proportions of MRTF^epiDKO^ and Control and identify potential markers for these cell states. Originating datasets, pseudotime states, and cell cycle state colorings were used within generated graphics.

Receptor–ligand expression analysis: Using published lists of pairings from Ramilowski et al.^63^, the receptor–ligand pairings were converted to MGI gene symbol from HGNC gene symbol using biomaRt (2.42.0)^64,65^. Ligands that were shown to be differentially expressed within the whole-transcriptome sequencing of the MRTF^epiDKO^ epicardial cells in comparison to the Control were flagged for later consideration.

Both the endothelial and epicardial datasets were filtered for expressed receptors and ligands, respectively. Ligands expressed within the epicardial data set were categorized as being differentially expressed between mesothelial and mesenchymal cell populations. Receptors expressed within the E14.5 MRTF^epiDKO^ and Control combined data set were characterized as differentially expressed between the two conditions. Seurat’s DotPlot and doHeatMap functions were used to visualize differential expression across both datasets.

For network visualization, tidyverse (1.3)^66^ was used for data analysis, viridis (0.5.1) (https://cran.r-project.org/web/packages/viridis/index.html) was used for color mapping, and both igraph (1.2.4.2) (https://igraph.org/) and ggraph (2.0.1) (https://cran.r-project.org/web/packages/ggraph/index.html) were used to generate and plot the network map. Epicardial ligands and endothelial receptors were grouped together and colored based on differential regulation; green if they were solely differentially regulated within that data set or red if they had a corresponding differentially regulated ligand or receptor. Red-lines connect receptors and ligand pairs, which were both confirmed to be differentially expressed. The epicardial ligands were further colored by expression in specific cell populations identified as mesothelial, mesenchymal, or general epicardial.

### Whole-transcriptome sequencing of epicardial cells

The Clontech Ultralow RNA Kit in conjunction with NexteraXT DNA Library Prep Kit (Illumina) was used for next-generation sequencing library construction according to the manufacturer’s protocols. Briefly, mRNA was purified from 1 ng total RNA with oligo-dT magnetic beads and fragmented. First-strand cDNA synthesis was performed with random hexamer priming followed by second-strand cDNA synthesis using dUTP incorporation for strand marking. End repair and 3′ adenylation was then performed on the double stranded cDNA. Illumina adaptors were ligated to both ends of the cDNA, purified using Ampure beads, and amplified with PCR primers specific to the adaptor sequences to generate cDNA amplicons of ~200–500 bp in size. The amplified libraries were hybridized to the Illumina single-end flow cell and amplified using the cBot (Illumina). Single-end reads of 100nt were generated for each sample using Illumina’s HiSeq2500v4. Raw reads were generated from Illumina HiSeq2500 sequencing and demultiplexed using bcl2fastq version 1.8.4. Quality filtering and adapter removal were performed using Trimmomatic version 0.32 with the following parameters: “TRAILING:13 LEADING:13 ILLUMINACLIP:adapters.fasta:2:30:10 SLIDINGWINDOW:4:20 MINLEN:15”. Processed/cleaned reads were then mapped to the GRCm38 reference genome using the SHRiMP version 2.2.3 and the following parameters: “–qv-offset 33–all-contigs”. Uniquely aligned and multi-mapped reads were counted within the gencode GRCm38 gene annotations, in a strand-specific manner, using the cuffdiff tool from the cufflinks-2.0.2 package and the following parameters: “–FDR 0.05 -u -b GENOME”. Differential expression analyses and data normalization were performed using DESeq2-1.14.1R/Bioconductor package with an adjusted *p*-value (Benjamini–Hochberg) threshold of 0.05 within the R version 3.3.1 environment (https://www.R-project.org). The PCA plot was created given the top500 genes with the most variation using the stats-3.4.0 (prcomp) and rgl-0.98.1R packages. *K*-means clustering was performed on DEGs using log-transformed, normalized counts in Cluster 3.0 (http://bonsai.hgc.jp/~mdehoon/software/cluster/software.htm). Heat maps were generated using TreeView software (Version 1.1.6r4) and GraphPad (Version 8.4.2). Gene ontology analysis was performed using EnrichR and Ingenuity Pathway Analysis. Kyoto Encyclopedia of Genes and Genomes (KEGG) pathway analysis was performed using EnrichR.

### Epicardial explant culture and induction of EMT

In order to acquire primary epicardial cells for in vitro experiments, pregnancies were timed to obtain E11.5 embryos from C57BL/6J female dams^34^. On the day of isolation, pregnant dams were anesthetized and administered ketamine-xylazine via intraperitoneal injection followed by cervical dislocation. After removal of decidua, embryos were placed in pre-warmed HBSS, extraembryonic tissue and the yolk sac were dissected, and hearts were extracted from the embryo and placed dorsal side down on collagen-coated culture wells (Corning, 354557) and incubated at 37 °C and 5% CO_2_ for 30 min to allow adhesion of hearts to the collagen matrix. Following incubation, media composed of M199 supplemented with 5% FBS and 1% Pen-Strep was added slowly around the hearts (50–100 μL) and incubated at 37 °C and 5% CO_2_ for approximately 24 h to allow for epicardial outgrowth. Next day, hearts were removed using fine-tip forceps and the epicardial cell monolayer was washed 2 times with DPBS. Primary epicardial cells were then treated with culture media (M199 with 1% FBS and 1% Pen-Strep) containing recombinant human TGF-β1 (10 ng/mL) and recombinant human PDGF-BB (20 ng/mL) to induce EMT for a total of 48 h (with replenishment of fresh media and recombinant factors after 24 h) at 37 °C and 5% CO_2_. After a total of 72 h in culture, epicardial cells were lysed in TRIzol Reagent and processed for RNA isolation. Gene expression analysis was performed with samples combined from two separate experiments. Vehicle (*n* = 6–7) and TGFβ1/PDGF-BB (*n* = 7–9).

### RNA isolation, cDNA biosynthesis, and quantitative RT-PCR

RNA was isolated using TRIzol Reagent according to the manufacturer’s instructions. RNA was treated with the TURBO DNA-free Kit (ThermoFisher Scientific, AM1907) to eliminate genomic DNA. Purified RNA was then made into cDNA using iScript cDNA Synthesis Kit (Bio-Rad, 1708891BUN). qRT-PCR was performed with cDNA, primers (Supplementary Table 1), and IQ SYBR Green Supermix (Bio-Rad, 1708887). Data were analyzed using the ΔΔ*C*(*t*) method. Samples with insufficient melt curves were not used in analyses. Due to the low yields of RNA following enrichment or culture of embryonic cardiac cells, varying *n* displayed in Figs. 4, 5, and 8 and Supplementary Fig. 23 are reflective of samples that could not be included in all gene expression analyses due to insufficient cDNA quantities.

### In situ hybridization assays

Embryonic hearts from Wt1^CreERT2/+^; R26^mTmG/+^ (E12.5 and E16.5), Cspg4^CreERT2/+^; R26^mTmG/+^ (E17.5), Wt1^CreERT2/+^ (E14.5), and Wt1^CreERT2/+^; Mrtf-a^−/−^; Mrtf-b^flox/flox^ (E14.5) were harvested and fixed in 10% neutral buffered formalin (NBF; ThermoFisher Scientific, 22-110-869) in DPBS for 18–24 hs at room temperature on a rocking platform. After fixation, tissue was dehydrated in an ethanol series followed by xylene before embedding tissue in paraffin wax and cutting hearts into 5 μm sections using a microtome. After sectioning, slides were allowed to dry overnight at room temperature and stored with desiccants for long-term storage. In order to perform in situ hybridization, we utilized the RNAscope Multiplex Fluorescent V2 Assay (Advanced Cell Diagnostics, 323100) as per the manufacturer’s instructions for formalin-fixed paraffin-embedded (FFPE) tissue, and with small modifications. Manual antigen retrieval was performed for 10 min and RNAscope protease plus solution was added for 30 min to each section. Following pre-treatment protocols, a combination of 2–3 mRNA probes (Supplementary Table 2) was hybridized for 2 h at 40 °C and stored at room temperature in 5× Saline Sodium Citrate (SSC) overnight (16–18 h). The next day, amplification of probes was performed by hybridization and the development of horseradish peroxidase (HRP) to C1, C2, or C3 conjugated probes followed by addition of Tyramide-Signal Amplification (TSA Plus Fluorescein, Cyanine 3 and Cyanine 5; Perkin Elmer, NEL741001KT, NEL744001KT, and NEL745001KT) to fluorescently label probes (Supplementary Table 2) in a sequential manner. DAPI was added to sections after the last wash step for 30 s, and slides were mounted with ProLong Gold Antifade Mountant (ThermoFisher Scientific, P10144), coverslipped (#1.5 mm), and imaged using an Olympus Confocal Microscope IX81 (Olympus Corporation).

### Immunohistochemistry

Embryonic hearts from Wt1^CreERT2/+^; R26^mTmG/+^ (E12.5 and E16.5), Wt1^CreERT2/+^ (E14.5 and E17.5), and Wt1^CreERT2/+^; Mrtf-a^−/−^; Mrtf-b^flox/flox^ (E14.5 and E17.5) were harvested and fixed in 4% paraformaldehyde (PFA; Electron Microscopy Sciences 15710) in DPBS for 18–24 h at room temperature. After fixation, tissue was dehydrated in an ethanol series followed by xylene before embedding in paraffin wax. Hearts were then cut at 5 μm sections using a microtome, baked at 60 °C overnight, and stored at room temperature for long-term storage. To begin immunostaining, slides were deparaffinized in a series of xylenes, followed by 3-min incubations in 100% ethanol (EtOH, 3 times), 95% EtOH (1 time), and then placed in distilled water. Antigen retrieval was performed in pH6 Dako Target Antigen Retrieval buffer (Agilent Technologies, S169984-2) followed by an incubation with 3% H_2_O_2_ in 15 mM NaCl/100 mM Tris pH 7.5 (TN buffer) to quench endogenous HRP. To prevent non-specific binding of antibodies, TSA Blocking Reagent (Perkin Elmer, FP1012) diluted at 0.5% in TN wash buffer (TNB) was added for at least 30 min to sections at room temperature. After the blocking step, primary antibodies diluted in TNB were incubated on sections overnight at 4 °C. After overnight incubation (16–18 h), slides were washed 3 times in TN buffer and incubated in secondary antibodies diluted in TNB for 2 h at room temperature. Amplification was performed as necessary. Biotin secondaries were amplified by adding a streptavidin-conjugated fluorophore for an additional 1 h at room temperature in TNB after performing 3 washes of biotin secondary. HRP secondaries were amplified by using the TSA Plus Fluorescent system for 10 min at room temperature in amplification diluent (provided in TSA Plus kit) after performing 3 washes of an HRP secondary. Following, secondary or tertiary incubations, slides were washed 3 more times in TN buffer with the final wash containing DAPI (0.5 μg/mL) for at least 10 min to stain nuclei. Slides were mounted with VECTASHIELD Anti-Face Mounting Media (Vector Labs, H-1000) before being imaged on an Olympus Confocal Microscope IX81 (Olympus Corporation). Primary Antibodies and dilutions: Rabbit anti-GFP (1:200, Torrey Pines Biolabs Inc., TP401), Goat anti-PDGFRα (1:100, R&D Systems, AF1062), Mouse anti-MYL2 (1:50, Santa Cruz Biotechnology sc517244), Mouse anti-cTNT (1:100, ThermoFisher Scientific, PIMA512960), Rabbit anti-ERG (1:200, Abcam, ab92513), Rat anti-EMCN (1:100, eBioscience, 14-5851-85), Rabbit anti-Cx40 (1:100, Alpha Diagnostic, CX40-A), Rabbit anti-HA-Tag (1:100, Cell Signaling Technology, 3724s). Secondary Antibodies and dilutions: Donkey anti-Rabbit Biotin (1:500, Jackson ImmunoResearch Laboratories, 711-065-152), Bovine anti-Goat HRP (1:200, Jackson ImmunoResearch Laboratories, 805-035-180), Donkey anti-Rabbit HRP (1:200, Jackson ImmunoResearch Laboratories 711-035-152), Streptavidin-555 (1:100, ThermoFisher Scientific, S21381), Tyramide FITC (1:200, Perkin Elmer, NEL741E001KT), Tyramide Cy3 (1:200, Perkin Elmer, NEL744E001KT), Donkey anti-Mouse 647 (1:200, Jackson ImmunoResearch Laboratories, 715-605-151), Donkey anti-Rat 488 (1:200, Jackson ImmunoResearch Laboratories, 703-545-155), Donkey anti-Rabbit 488 (1:100, Jackson ImmunoResearch Laboratories, 711-545-152).

### Quantitation of endothelial cell polarity

The evaluation of nuclear polarity in embryonic tissue was performed following immunostaining of hearts with endothelial-specific nuclear protein marker ERG, which was counterstained with DAPI to visualize nuclei. Quantitation of nuclear dimensions of ERG^+^ nuclei and total nuclei was performed using ImageJ (Fiji Version: 2.0.0-rc-69/1.52p). Specifically, to measure EC nuclei, single scans of ERG and DAPI labeling (imaged via Confocal Microscope Olympus BX41 at ×60) were colocalized using the Colocalization Threshold function in ImageJ software, automatically creating an image of all ERG^+^ and DAPI^+^ nuclei. Subsequently, the images were filtered to a threshold to obtain a binary image that was watershed, and images were analyzed through the Analyze Particles function. Nuclear dimensions were evaluated via the Feret’s Diameter function, and the nuclear length to width ratio was determined by dividing the Feret value by the minimum Feret of each cell^67^. At E14.5, 5 Control hearts and 3 MRTF^epiDKO^ hearts were analyzed. At E17.5, 6 Control hearts and 3 MRTF^epiDKO^ hearts were analyzed. For each heart, at least three fields of view were assessed.

### Quantitation of endothelial cell localization

Evaluation of ERG^+^, EMCN^+^, and Cx40^+^ cell localization starting from the surface of the heart (epicardium) was performed using ImageJ software. The DAPI channel was used to delimit the epicardium layer, defined as the outer layer of nuclei. Each channel/protein was processed with a smoothing filter, adjusted for brightness and contrast, and filtered to obtain a mask. In order to minimize manual errors, an automated script was written to measure the distances of each channel/protein to the epicardium layer. The masks obtained in ImageJ provided the input for the script. The script was written in Python^68^ and utilized the image processing packages scikit-image^69^ and mahotas^70^. At E14.5, 4 Control hearts and 3 MRTF^epiDKO^ hearts were analyzed. At E17.5, 5 Control hearts and 3 MRTF^epiDKO^ hearts were analyzed for ERG^+^ cells and 4 MRTF^epiDKO^ hearts were analyzed for EMCN^+^ and Cx40^+^ cells. For each heart, at least three fields of view were assessed.

### Statistical analyses

Data were expressed as mean ± SEM for bar graph data presented and statistical analyses were performed using unpaired two-tailed Student’s *t*-test when comparing two groups. All measurements in this paper were acquired from distinct samples and no samples were measured repeatedly. Bar graph data analysis was performed using GraphPad Prism 8 for macOS (Version 8.4.2). Statistical analysis of endothelial cell localization was performed using a two-tailed Mann–Whitney test. A value of *p* < 0.05 was considered statistically significant.

### Reporting summary

Further information on research design is available in the Nature Research Reporting Summary linked to this article.

## Supplementary information

Supplementary Information

Description of Additional Supplementary Files

Supplementary Dataset 1

Supplementary Dataset 2

Supplementary Dataset 3

Supplementary Dataset 4

Supplementary Dataset 5

Supplementary Dataset 6

Supplementary Dataset 7

Reporting Summary

## Data Availability

All transcriptomic analyses were performed using standard protocols with previously described R packages in the methods. Analysis of endothelial cell localization was determined using Python script described in the methods. R and Python scripts mentioned in this manuscript are available upon request.

## Source data

Source Data

## Data availability

Bulk RNA-sequencing data from epicardial cells have been deposited in the Gene Expression Omnibus database under accession code “GSE153367”. Single-cell transcriptomic analysis of epicardial cells and endothelial cells data generated in this study have been deposited in the Gene Expression Omnibus database under accession code “GSE154715”. All other relevant data supporting the key findings of this study are available within the article and its Supplementary Information files or from the corresponding author upon reasonable request. Source data are provided with this paper.
