# Supplementary material for: Coordination of endothelial cell positioning and fate specification by the epicardium
Source: Nat Commun. 2021 Jul 6;12:4155. doi: 10.1038/s41467-021-24414-z (PMC8260743; doi:10.1038/s41467-021-24414-z)
Supplement: Supplementary file 1 — Supplementary Information [file 41467_2021_24414_MOESM1_ESM.pdf]

Supplementary Information

Coordination of endothelial cell positioning and fate specification by the epicardium

Pearl Quijada[1,2], Michael A. Trembley[1], Adwiteeya Misra[1,3], Jacquelyn A. Meyers[4,5], Cameron Baker[4,5], Marta Pérez-Hernández[6], Jason R. Myers[4,5], Ronald A. Dirkx Jr.[1], Ethan David Cohen[7], Mario Delmar[6], John M. Ashton[4,5], and Eric M. Small[1,5,8*]

Contents:

Supplementary Tables 1 & 2

Supplementary Figures 1-25

Supplementary Table 1. List of Primers

Primers for qRT-PCR		
gene target	forward primer	reverse primer
18s	CGAGCCGCCTGGATACC	CATGGCCTCAGTTCCGAAAA
Acta2	GTTCAGTGGTGCCTCTGTCA	ACTGGGACGACATGGAAAAG
Aldh1a2	TCTATGCCGATGTGAGAAG	ATATGGGAGCCCTCATCAAG
Apln	TGAATCTGAGGCTCTGCGTG	ACATCAGTGGCACTCCACAA
Aplnr	CCTCCATGCTCTGCTGTGAT	GCCCGGAAGAATAACTGGCT
Col3a1	TAGGACTGACCAAGGTGGCT	GGAACCTGGTTTCTTCTCACC
Cspg4	TCTTACCTTGGCCTTGTTGG	ATGTGGAGAACTGGAGCAGC
Cxcr4	TGCAGCAGGTAGCAGTGAAA	TGTATATACTCACACTGATCGGTTC
Dll4	CCAGCAACCCCTGTCGAAAT	GCTATTCTCCTGGTCCTTACAGC
Efna5	CTACATCTCCTCTGCAATCCCA	GGCTGACTCATGTACGGTGT
Efnb2	TGTGTGGAAGTACTGTTGGGG	GCTATTCTCCTGGTCCTTACAGC
Epha7	ACTGCTGCCGGTTATGGAAA	ACTGCTGTCGCTTCAAACA
Ephb4	CTACCTTAGCCACTGGACCAC	ATGCGCCCCTCACGGTC
Flrt2	TGTGCGCTGGGAGATAACAC	ACCAGATAAGAGCGCACCTG
Gja4	AACGGTGCTCTTCATCTTCCGC	GGTCATAGCAGACGTTGGTGCA
Gja5	GAAGAAGCAGCCAGAGTGTGAAG	GGGAACAGATGCCAAAACTTCT
Mrtfa	AGCCAACCTGGATGACATGAA	CGCAGGCGTTCTATCAGCTC
Mrtfb	TCCCGTGCTCCCTACAA	CGGTGTTTGTCGTTTGGATTC
Myh7	GTGGCTCCGAGAAAGGAAG	GAGCCTTGGATTCTCAAACG
Nr2f2	CCTATAAATCGCATTCCCTCCC	CGCGGAGAGAAAAGAACAGAGA
Nr2f2	TGCGGAGGAACCTGAGCTAC	CTGTACAGCTTCCCGTCTCAT
Pard3	AAGCAAGTGCAATGAGCTGAA	CCTACTGAGGGCAGCATTTC
Pard6	ATACGGATGCTCATGGCGAC	AGTCAGCTTCTGCCCGCTTC
Pdgfra	GGGAGAGAAACAAACGGAGGA	GCTCCTGAGACCTTCTCCTTCTA
Pdgfrb	GGGAGACACTGGGGAATACTTTTG	TGAACAGGTCCTCGGAGTCCATAG
Pecam1	TGGTTGTCATTGGAGTGGTC	TTCTCGCTGTTGGAGTTCAG
Postn	GACTGCTTCAGGGAGACACA	TGATCGTCTTCTAGGCCCTT
Sema3c	AAGGAGGTCAGAGGACCAGG	CGGGTTGAAAGAGCATCGTC
Sema3d	TGTCTCGCCAGAGACCCTTA	AGCTCGCCTTTTAGACGTGG
Slit2	GGGTGGGCTTGTACACTCAG	TCTTCCTCATCACTGCAGACAA
SLIT2	TCCGTTGTTCAGGTACAGAAGAT	GCGACACTTTCAGGGCAAG
Slit3	CCACCAAGTGTACCTGCTCC	TTTCTGTCCAGGTCAAGGCG
Sox9	GAAGCTGGCAGACCAGTACC	GGTCTCTTCTCGCTCTCGTTC
Srf	CACCTACCAGGTGTCGGAAT	GTCTGGATTGTGGAGGTGGT
Tbx18	CAGAATCAGCAGATTACTCGCC	GGCCTCCAGAATGCGTATGA
Tcf21	CATTCACCCAGTCAACCTGA	CCACTTCCTTCAGGTCATTCTC
Tnc	TGATACTACCTCTGGCCTCTAC	AACAATCCATCCACCTCCATC
Tnnt2	TCGACCACCTGAATGAAGACC	TTCCTGCAGGTCGAACTTCTC
Upk3b	TCCACTACGCGTTTCTCCAG	ATGAAGGAGCCAATCCGCAG
Wnt5a	AATTCCTCGGCCGCCTTCGC	GCGGTCCCCAAAGCCACTCC
Wt1	ATCCGCAACCAAGGATACAG	GGTCCTCGTGTTTGAAGGAA
Zeb2	AGGCGCGAGAGAAAGGGCAC	CCCGGTTCATCAGCAGCTCGG
Bio-Rad Primers for qRT-PCR		
gene target	catalog#	
Angptl2	qMmuCID0020392	
Col5a1	qMmuCID0021746	
Col18a1	qMmuCID0015488	
Dlk1	qMmuCID0007317	
Efna5	qMmuCID0017765	
Fgf2	qMmuCID0015817	
Fgf9	qMmuCID0005457	
Gpc3	qMmuCID0013051	
Hprt	qMmuCID0005679	
Hspg2	qMmuCID0021282	
Lama4	qMmuCID0005280	
Lpl	qMmuCID0020151	
Mfge8	qMmuCID0040120	
Nid1	qMmuCID0018281	
Pdgfa	qMmuCID0022342	
Ptn	qMmuCID0005591	
Rarres2	qMmuCID0024298	
Sema3d	qMmuCID0019844	
Sema5a	qMmuCID0018023	
Serpine2	qMmuCID0017042	
Sfrp1	qMmuCID0018787	
Slit2	qMmuCID0005159	
Slit3	qMmuCID0021154	
Tfpi	qMmuCID0025771	
Tgfb2	qMmuCID0024408	
Tgm2	qMmuCID0005401	
Thbs1	qMmuCID0014108	
Thbs2	qMmuCID0015318	
Wnt5a	qMmuCID0005764	

Supplementary Table 2. RNAscope in situ probes and secondary fluorophores.

RNAscope probes for *in situ* hybridization assays		
gene target	catalog #	secondary & dilution
Egfp	400281-C1	TSA Plus Fluorescein; 1:750
Slit2	449691-C2	TSA Plus Cyanine 3; 1:1000
Sema3d	488111-C3	TSA Plus Cyanine 5; 1:1000
Egfp	400281-C1	TSA Plus Fluorescein; 1:750
Slit2	449691-C2	TSA Plus Cyanine 3; 1:1000
Pecam1	488111-C3	TSA Plus Cyanine 5; 1:1000
Egfp	400281-C1	TSA Plus Fluorescein; 1:750
Pecam1	316721-C2	TSA Plus Cyanine 3; 1:1000
Sema3d	488111-C3	TSA Plus Cyanine 5; 1:1000
Nrp1	471621-C1	TSA Plus Fluorescein; 1:750
Pecam1	316721-C2	TSA Plus Cyanine 3; 1:1000
Sema3d	488111-C3	TSA Plus Cyanine 5; 1:1000
Robo4	466321-C1	TSA Plus Fluorescein; 1:750
Slit2	449691-C2	TSA Plus Cyanine 3; 1:1000
Pecam1	316721-C3	TSA Plus Cyanine 5; 1:1000

Supplementary Figure 1

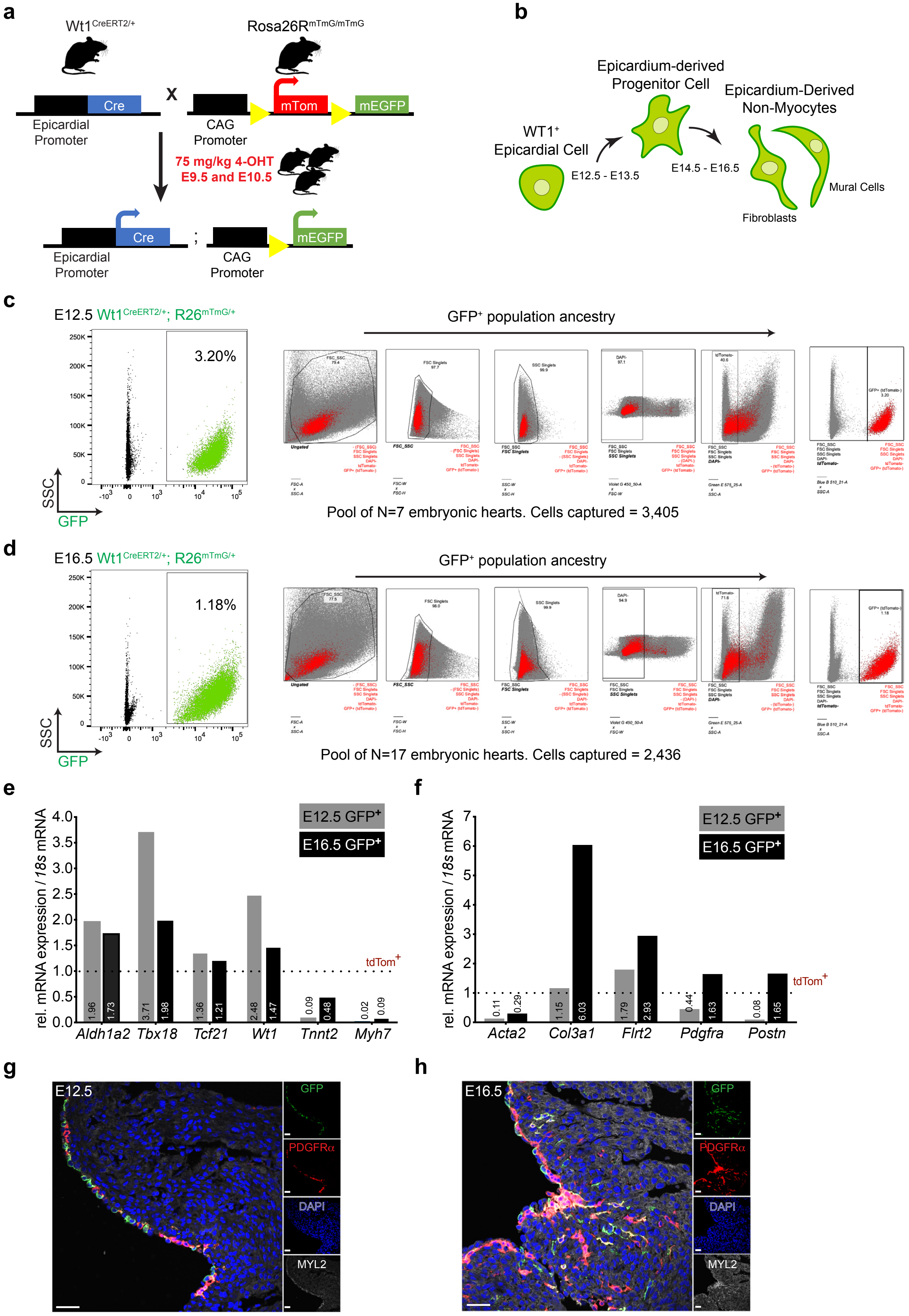

Supplementary Figure 1. Labeling and acquisition of epicardial cells during embryonic development. a, Epicardium-derived cells (EPDCs) were labeled by crossing the heterozygous $Wt1^{CreERT2/+}$ mouse to a two-fluorescent and membrane tethered $Rosa26R^{mTmG/mTmG}$ mouse reporter strain. Pregnant dams were administered 4-Hydroxytamoxifen (4-OHT) at a concentration of 75μg/kg at embryonic day (E)9.5 and E10.5 to induce Cre-based recombination, deletion of the membrane tdTomato cassette and expression of membrane green fluorescent protein (GFP) in cells expressing *Wt1*. b, The *Wt1*-based lineage tracing model allows for visual tracing of GFP^+ cells, the rise of EPDCs from the epicardium and differentiation towards the non-myocyte lineages. c and d, Flow cytometric analysis and strategy to isolate GFP^+ epicardial cells from whole embryonic hearts at both E12.5 (n=7 hearts; 3,405 cells) and E16.5 (n=17 hearts; 2,436 cells), which were subsequently sorted into Eppendorf tubes and submitted for single-cell capture using the 10x Genomics Chromium Platform prior to single cell RNA-sequencing. FACS sequential gating strategy was identical for both E12.5 and E16.5 developmental time-points. First, single cells were selected based on FSC-A and SSC-A, FSC-W x FSC-H, and SSC-W x SSC-H. Then, single and viable cells were selected based on a negative DAPI stain. Elimination of tdTomato positive cells from the sort was performed by selecting the tdTomato negative population, followed by a positive selection of GFP^+ *Wt1*-lineage-derived cells from this tdTomato negative population. e and f, Bulk-captured GFP^+ epicardial cells were analyzed for marker gene expression at either E12.5 or E16.5 by qRT-PCR. Gene expression is presented as relative fold change as compared to the $tdTomato^+$ cell fraction sorted simultaneously. g and h, Immunohistochemical analysis of the spatial-temporal expression patterns of GFP (*Wt1*-lineage, green) and platelet-derived growth factor-α (PDGFRα, red) at E12.5 and E16.5. Scale bar, 25μm. MYL2, myosin regulatory light chain (white); DAPI (4',6-diamidino-2-phenylindole) staining was utilized to visualize nuclei (blue). Immunostaining was repeated independently 3 times with similar results.

Supplementary Figure 2

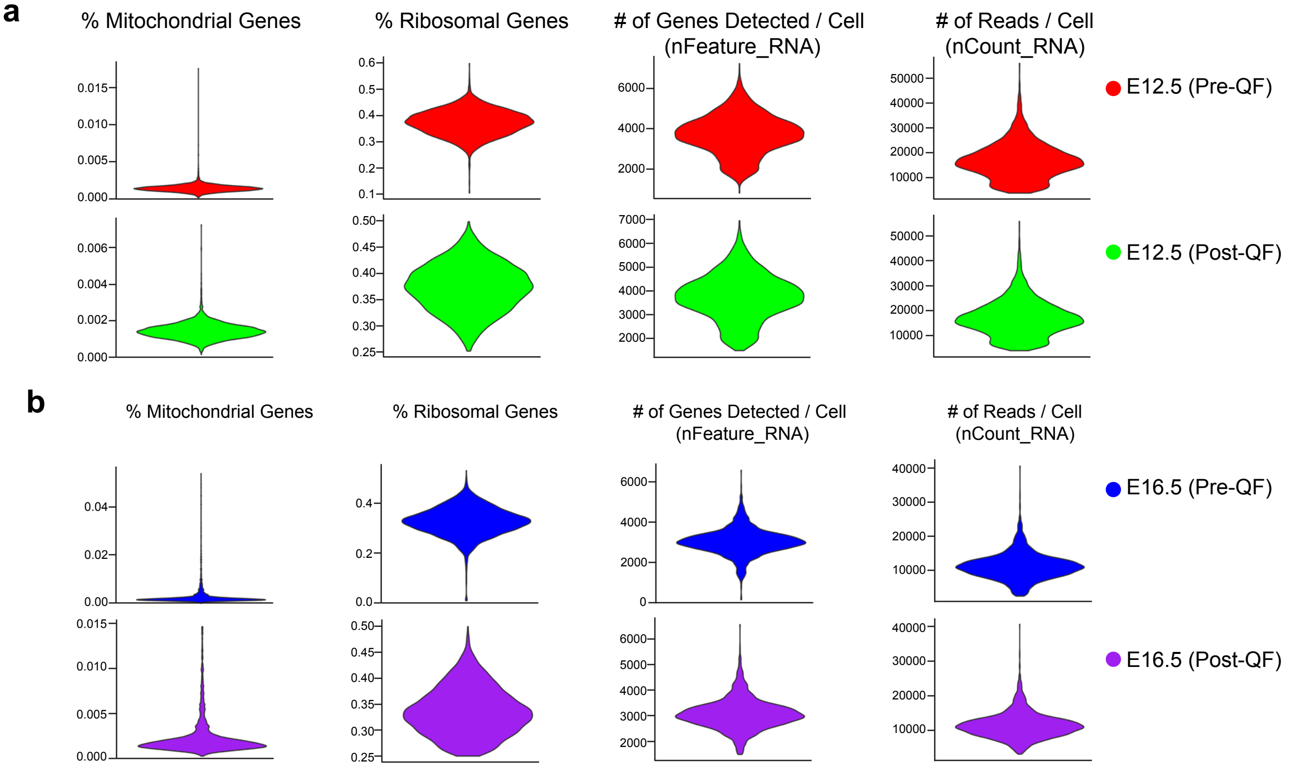

Supplementary Figure 2. Single cell RNA-sequencing quality control of epicardial cells. a and b, The percentage (%) of mitochondrial and ribosomal reads, number (#) of genes detected per cell (nFeature_RNA), # of reads per cell (nCount_RNA) were analyzed for quality filtering and presented as Pre-Quality Filtering (Pre-QF) versus Post-QF.

Supplementary Figure 3

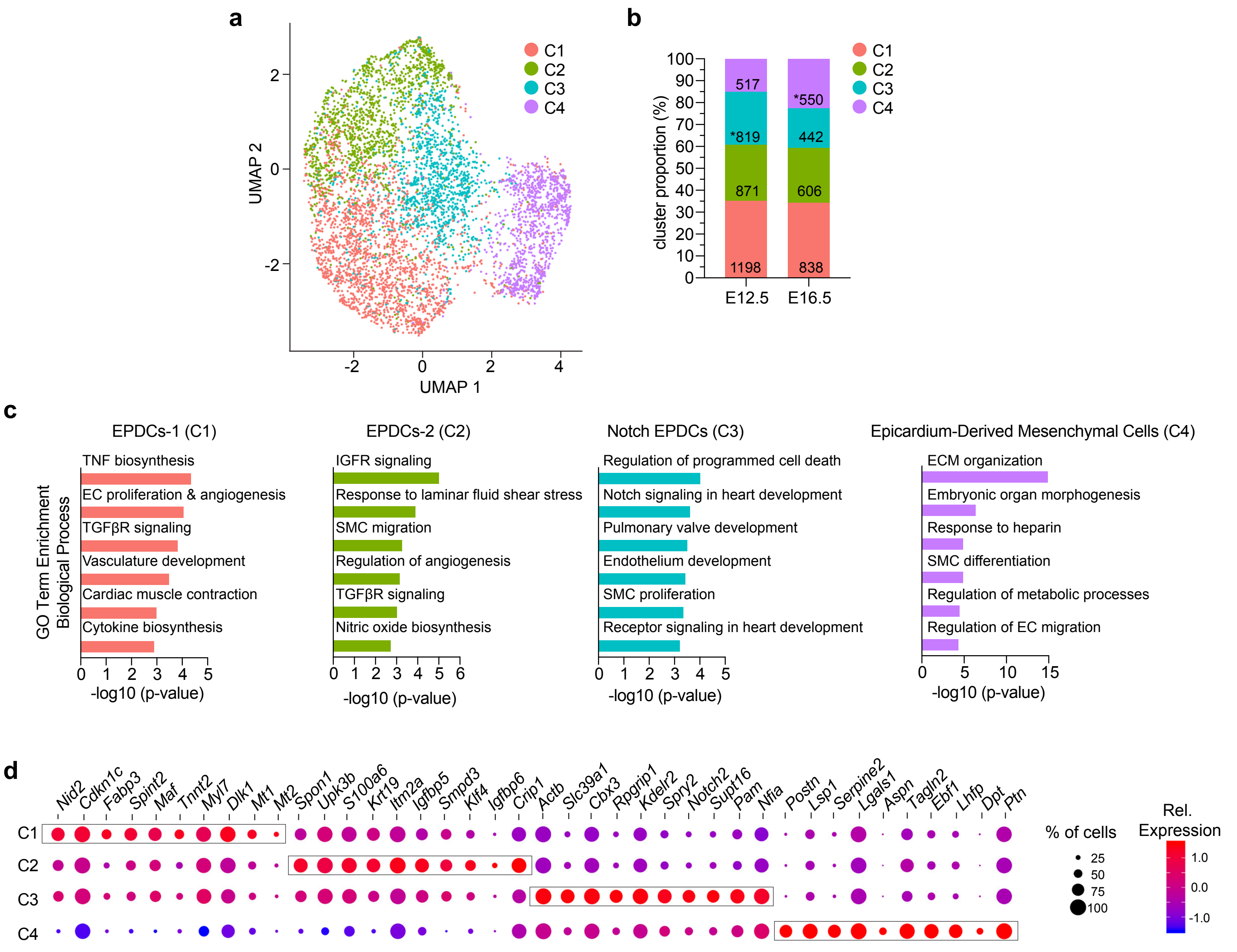

Supplementary Figure 3. Canonical Correlation Analysis (CCA) of epicardial cells acquired at E12.5 and E16.5. a, Uniform manifold approximation projection (UMAP) representation of embryonic day (E)12.5 and E16.5 epicardial cells following CCA. b, Relative contribution of epicardial cells to each of the four clusters represented by developmental age. *p<0.05 and a significant difference in the number of cells observed in cluster 3 (C3) and C4 and between E12.5 and E16.5. Two-sample student's t-test was performed to determine significance of the proportion of cells in single-cell clusters. c, Gene ontology (GO) analysis indicates most enriched biological processes within cells defined as epicardial-derived cells (EPDCs)-1 (C1), EPDCs-2 (C2), Notch EPDCs (C3), and Epicardium-Derived Mesenchymal Cells (C4). d, Dot plot presentation of the top 10 expressed genes in C1-C4.

Supplementary Figure 4

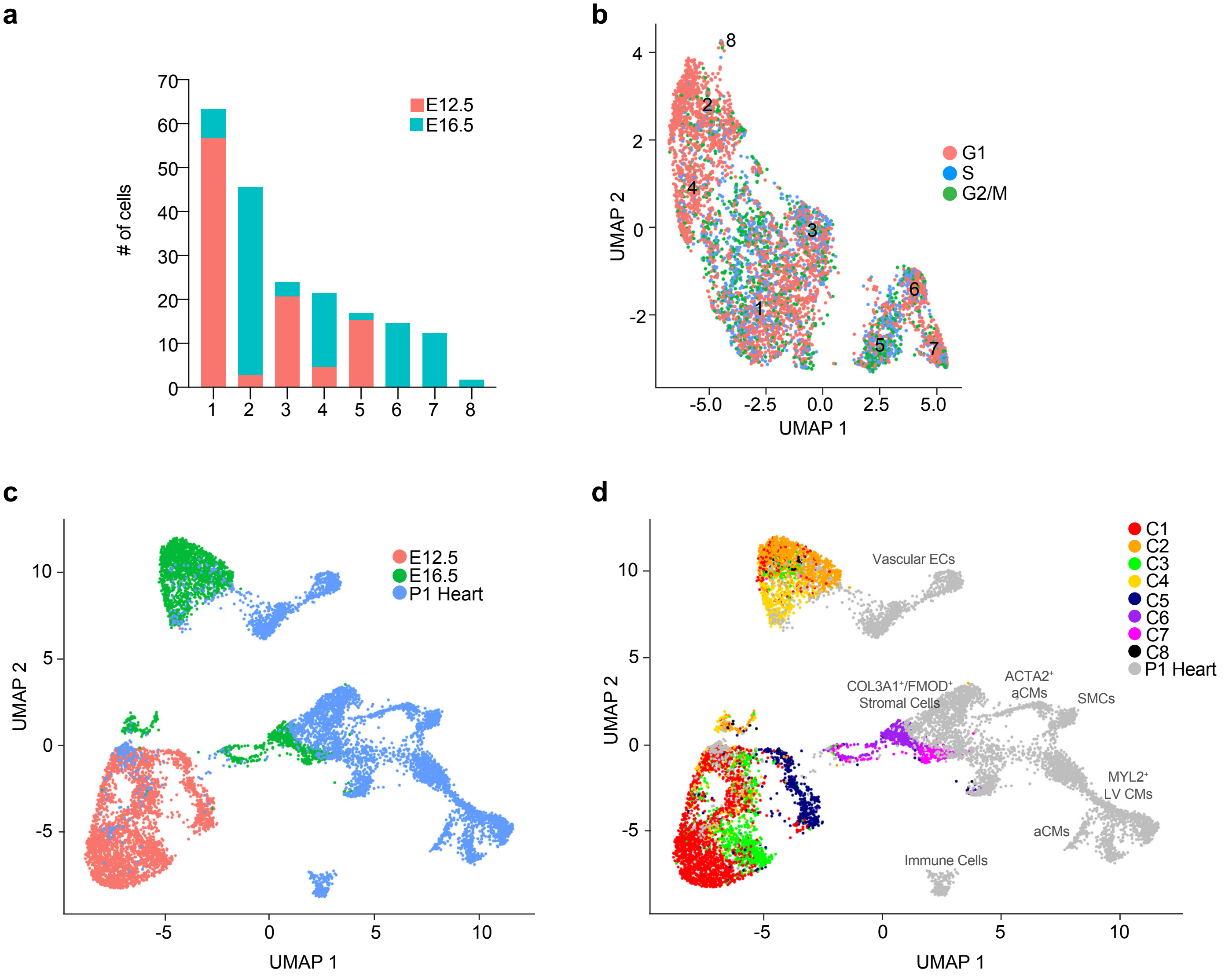

Supplementary Figure 4. Single cell transcriptomes of epicardial cells following merging of cells by developmental age. a, The number of cells from embryonic day (E)12.5 or E16.5 in each cluster identity. b, Uniform manifold approximation projection (UMAP) representation of cell cycle activity across 8 cell clusters. c and d, UMAP plot of epicardial cells after performing canonical correlation analysis (CCA) with cardiac cells (non-myocytes and cardiomyocytes) extracted from the postnatal day 1 (P1) heart. (c) UMAP plot of epicardial cells and P1 cardiac cells by developmental stage (d) and cell population clustering.

Supplementary Figure 5

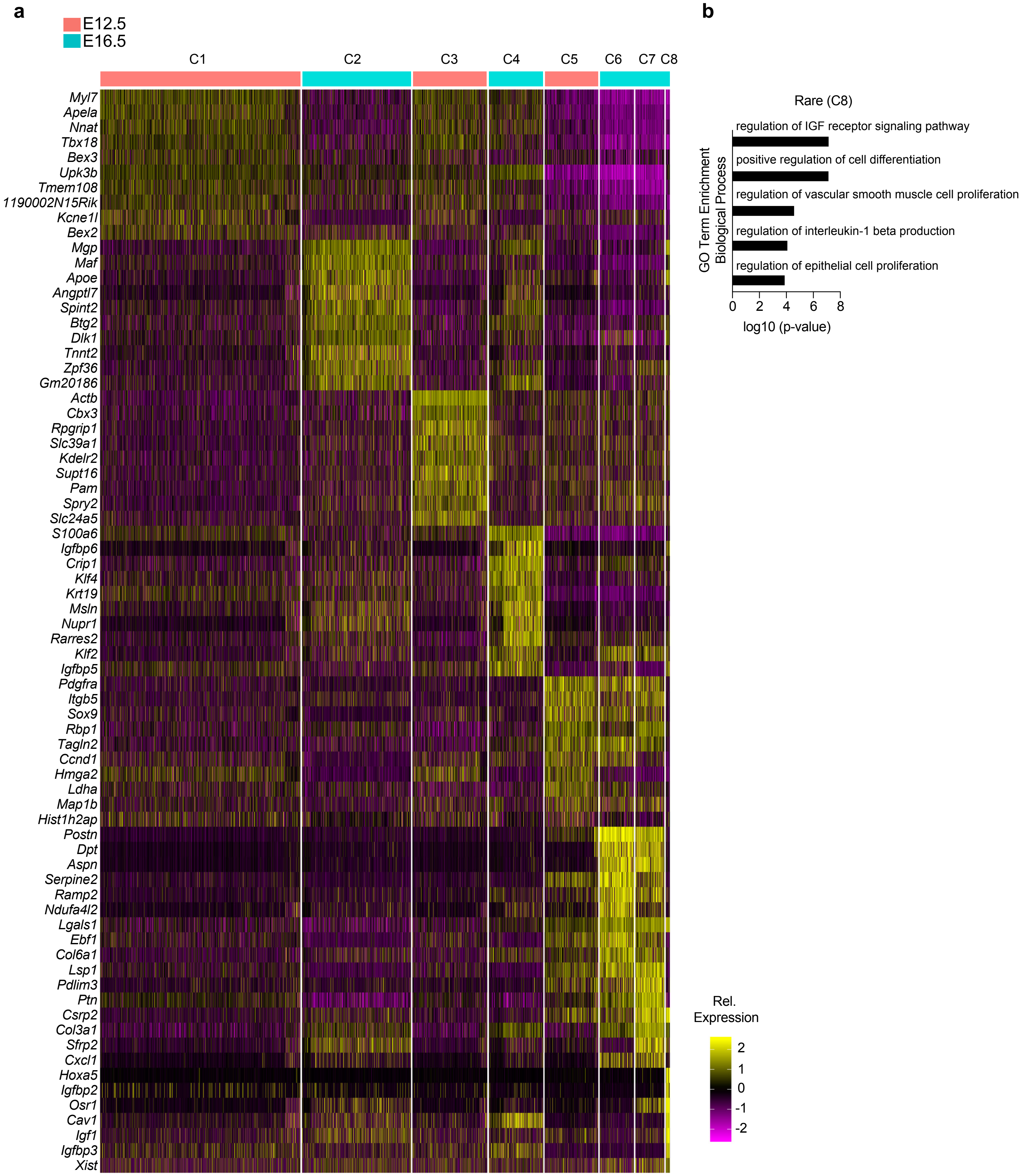

Supplementary Figure 5. Hierarchical gene expression of epicardial cell clusters. a, Expression heat map of the top differentially expressed genes in epicardial cells clusters 1-8. Scale represents relative (Rel.) expression values. b, Gene ontology (GO) terms associated with differentially expressed genes (DEGs) termed as “Rare” epicardial cells in Cluster 8. GO term enrichment significance was determined using Fisher exact test.

Supplementary Figure 6

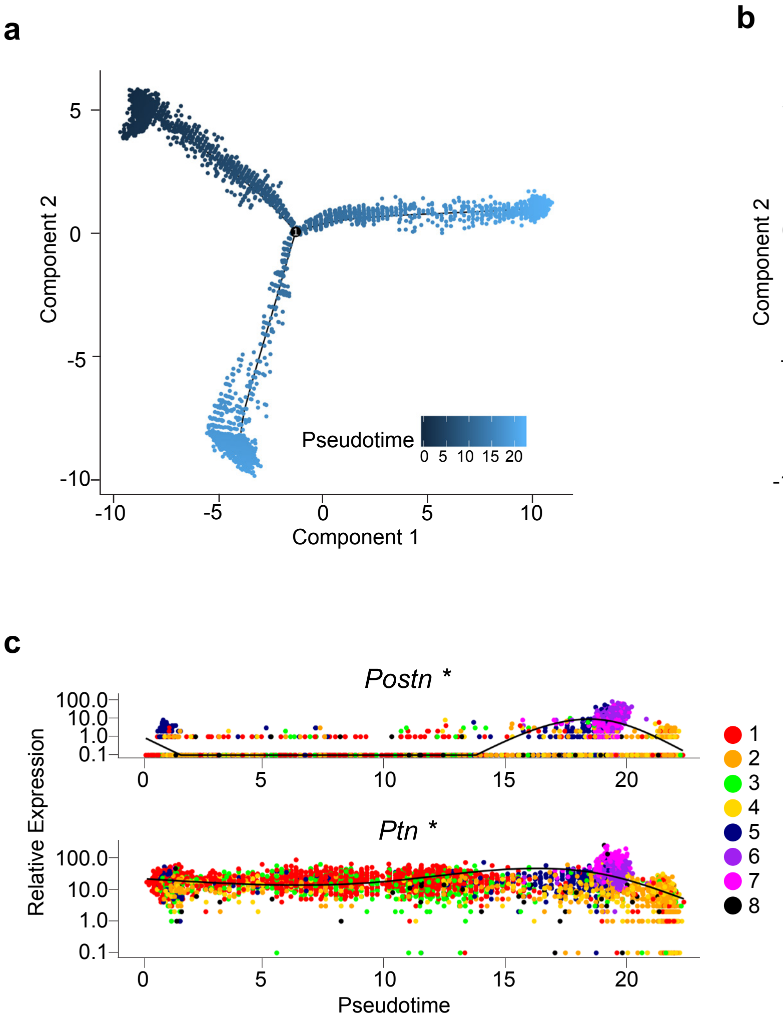

Supplementary Figure 6. Pseudotime trajectory analysis of epicardial cells. a and b, Pseudotime trajectory of epicardial cells as represented by (a) pseudotime score for each individual epicardial transcriptome and (b) pseudotime cell states. Cells in state 1 are at the beginning of pseudotime (left). Cell state 2 and 3 diverge from a common branchpoint (BP) located on the right. c, Pseudotime-dependent genes (*Postn* and *Ptn*) augmented in mesenchymal cells. Cells are colored according to cell cluster identity. *Genes with significant correlation with pseudotime state.

Supplementary Figure 7

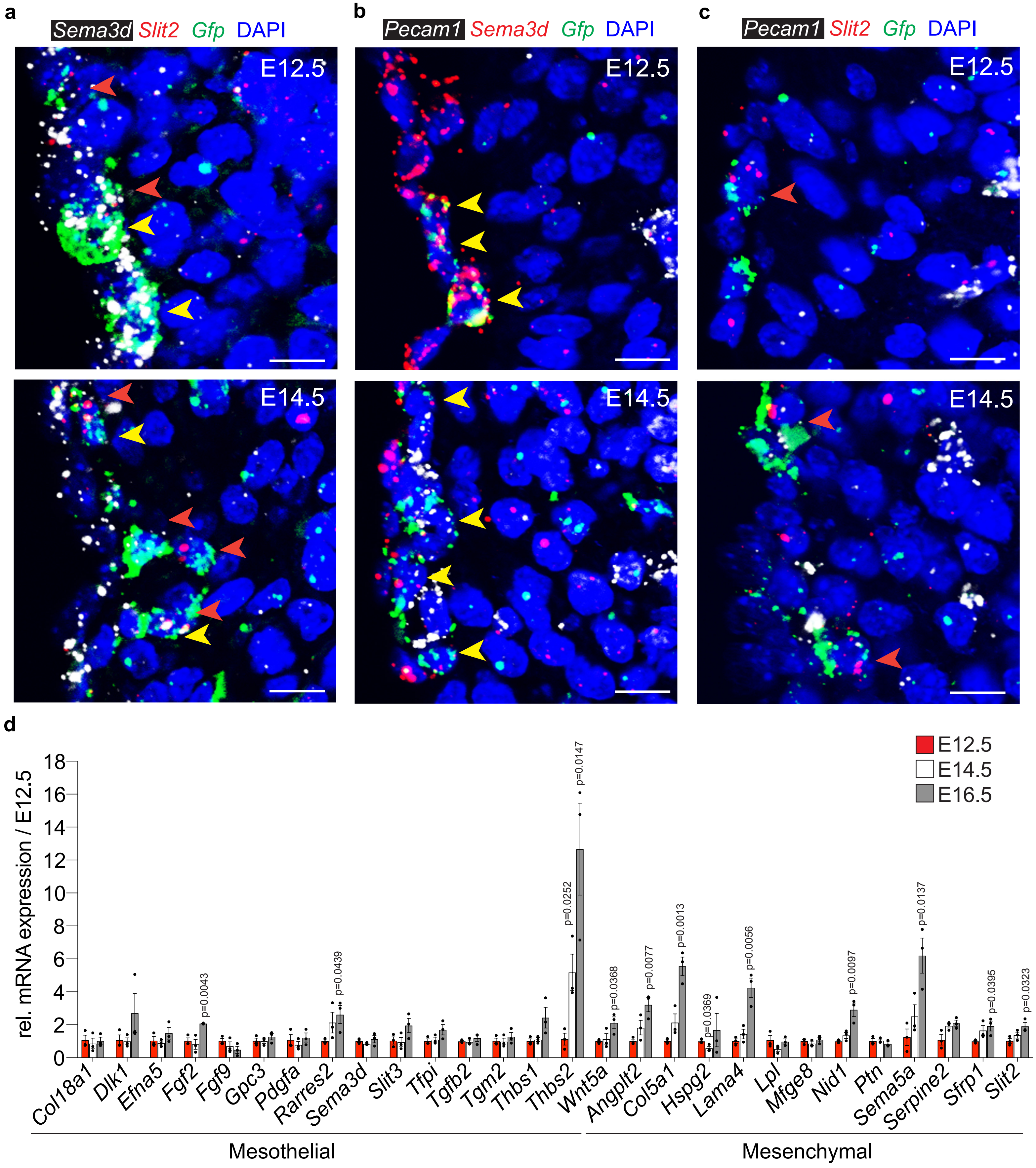

Supplementary Figure 7. Analysis of secreted ligands gene expression during cardiac development. a, Fluorescence in situ hybridization (FISH) using probes against *Gfp* (green) to detect epicardium-derived cells and *Sema3d* (white) or *Slit2* (red) reveals diverging localization of vascular guidance cues within the epicardium and interstitium between E12.5 and E14.5. b, Expression of *Sema3d* (red) in *Wt1* lineage-derived cells (Gfp^{+}, green) relative to Pecam1^{+} (platelet endothelial cell adhesion molecule 1, white) endothelial cells. c, Expression of *Slit2* (red) in *Wt1* lineage-derived cells (*Gfp*$^{+}$, green) relative to *Pecam1*$^{+}$ (white) endothelial cells. Yellow arrowhead, *Gfp*$^{+}$ *Sema3d*$^{+}$ cells. Orange arrowhead, *Gfp*$^{+}$ *Slit2*$^{+}$ cells. Scale bar, 10µm. DAPI (4',6-diamidino-2-phenylindole) staining was utilized to visualize nuclei (blue). d, *Wt1*-lineage cells were extracted from hearts at E12.5, E14.5 and E16.5 and analyzed for secreted ligand genes that were identified in either mesothelial or mesenchymal cell populations determined by single cell RNA-sequencing. Values are represented as a fold change in expression relative to E12.5 epicardial cells and n=3 samples were analyzed per timepoint. Each sample was isolated from at least 1 heart at the indicated developmental age. Data are presented as mean values +/- SEM. Statistical significance was determined by Two-sample unpaired student's t-test as compared to E12.5. For FACS sequential gating and enrichment of epicardial cell populations at E12.5, E14.5, and E16.5, refer to Supplementary Figure 1 c and d.

Supplementary Figure 8

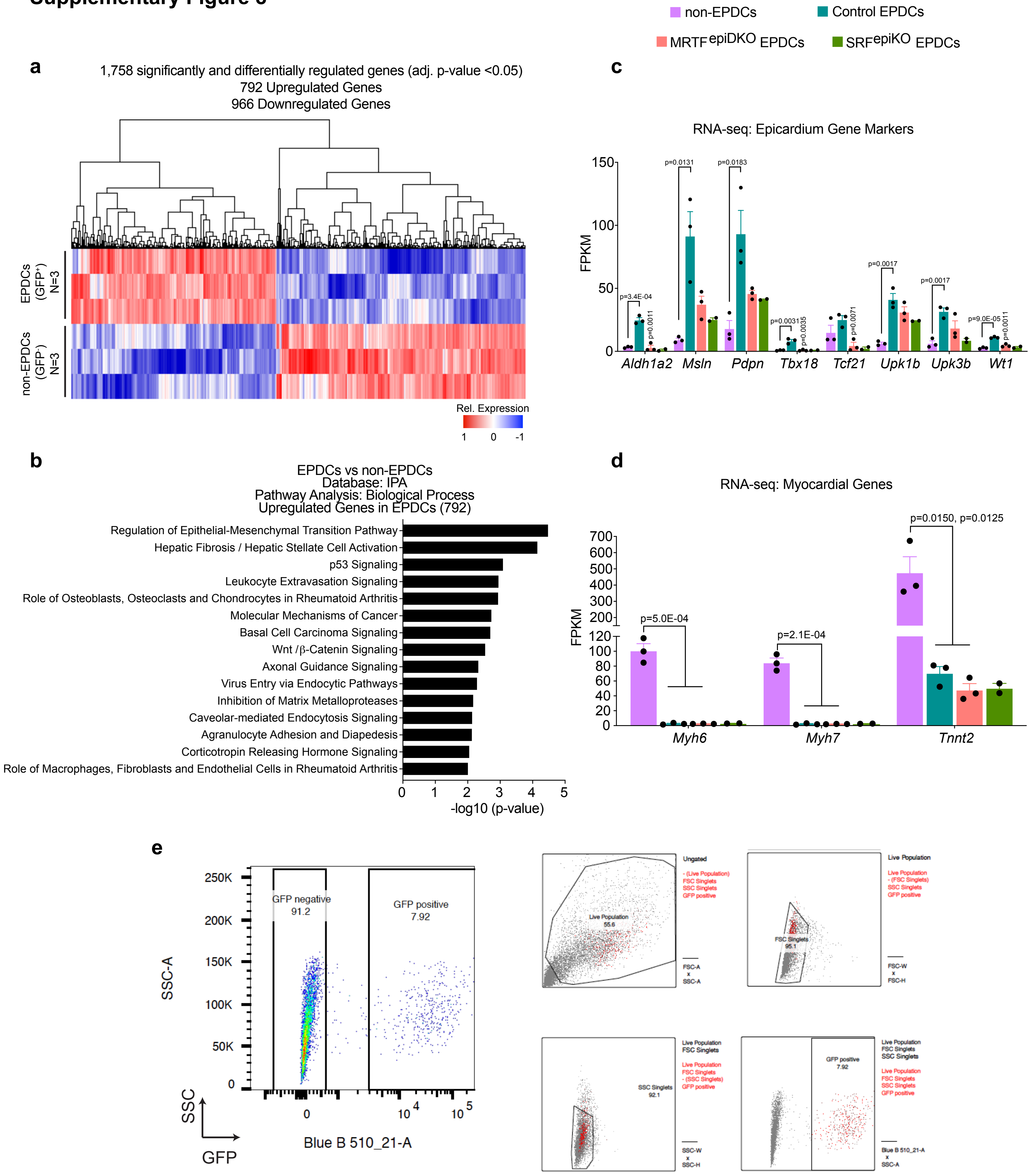

Supplementary Figure 8. Enrichment of epicardial cells for bulk RNA-sequencing. a, Gene expression heat map of differentially expressed genes between epicardium-derived cells (EPDCs) or non-EPDCs isolated from Control hearts. Scale represents relative (Rel.) expression. b, Ingenuity pathway analysis (IPA) of upregulated genes in Control EPDCs (792) and the top 15 gene ontology (GO) terms. Heat map and GO term enrichment significance was determined using Fisher exact test. c, Fragments per kilobase of transcript per million mapped reads (FPKM) values of established epicardial enriched genes and d, cardiomyocyte enriched genes in non-EPDCs as compared to EPDCs isolated from Control, myocardin-related transcription factor-A and -B ($MRTF^{epiDKO}$) and serum response factor (SRF^{epiKO}) mice. n=3 non-EPDCs, Control EPDCs, and $MRTF^{epiDKO}$ EPDCs and n=2 SRF^{epiKO} and examined over 1 experiment. Data are presented as mean values +/- SEM. Statistical significance was determined by Two-sample unpaired student's t-test. e, FACS sequential gating strategy for isolation of epicardial cells following infection of ex vivo hearts with adenovirus to express green fluorescent protein (GFP) was identical for all groups analyzed prior to RNA-sequencing. First, single cells were selected based on FSC-A and SSC-A, FSC-W x FSC-H, and SSC-W x SSC-H. Then, single epicardial cells were selected and enriched based on GFP fluorescence.

Supplementary Figure 9

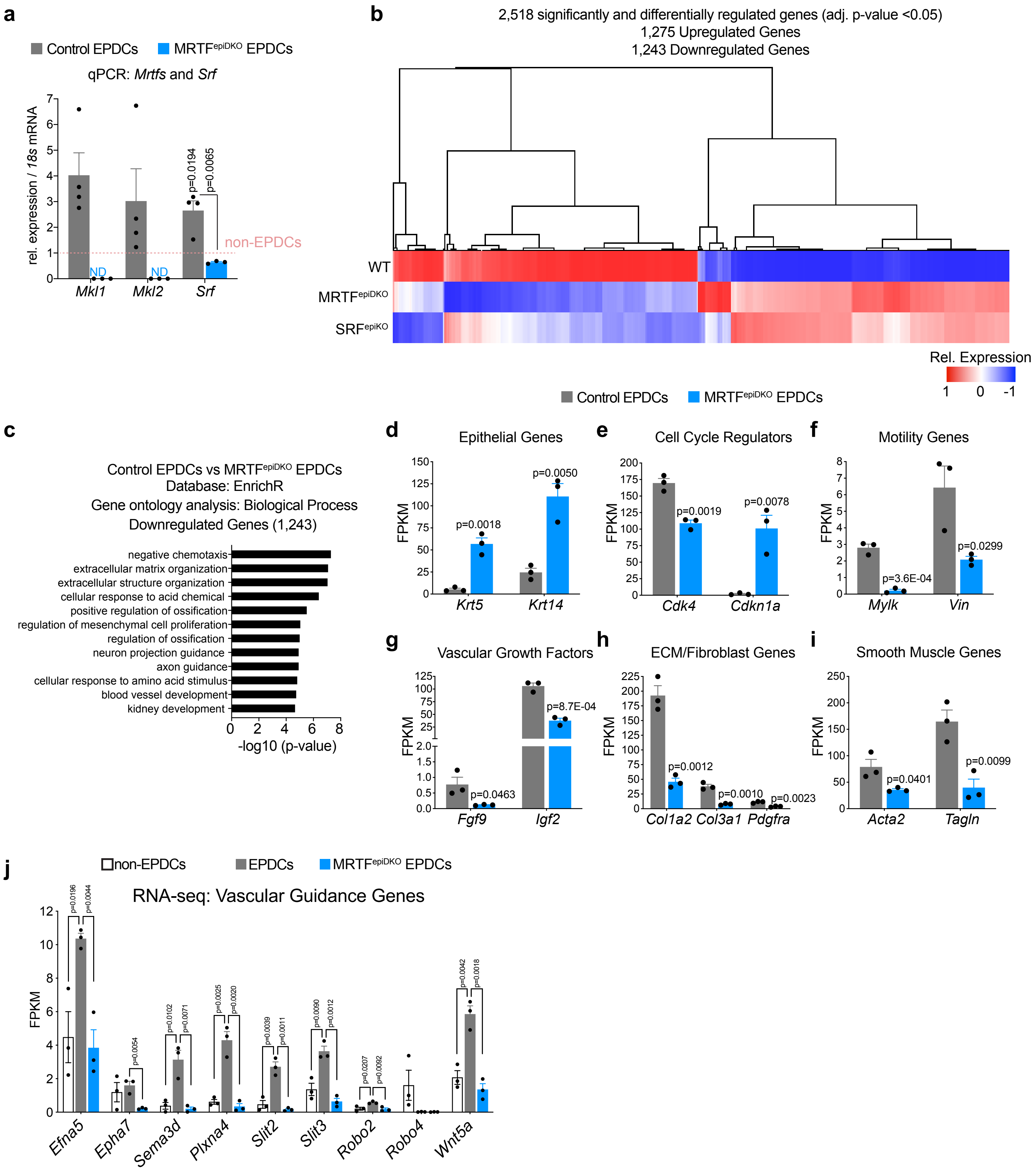

Supplementary Figure 9. Transcriptional analysis of epicardial cells from MRTF^{epiDKO} hearts. a, Expression of *Mkl1* (*Mrtfa*), *Mkl2* (*Mrtfb*) and *Srf* in epicardium-derived cells (EPDCs) and validated by qRT-PCR. Values are represented as a fold change expression relative to non-EPDCs (dashed line at 1). n=4 Control EPDCs and n=3 MRTF depleted EPDCs. b, Gene expression heat map of differentially regulated genes between epicardial cells isolated from Control or *Mrtf-a;Mrtf-b* double knockout (MRTF^{epiDKO}) hearts. Scale represents relative (Rel.) expression. c, Ingenuity pathway analysis (IPA) of downregulated genes (1,243) in EPDCs from MRTF^{epiDKO} hearts. Heat map and GO term enrichment significance was determined using Fisher exact test. d-i, Fragments per kilobase of transcript per million mapped reads (FPKM) values of genes associated with epithelial status (*Krt5*, *Krt14*), cell cycle regulation (*Cdk4*, *Cdkn1a*), migration (*Mylk*, *Vin*), vascular growth factors (*Fgf9*, *Igf2*), extracellular matrix production (*Col1a2*, *Col3a1*), and fibroblast (*Pdgfra*) or smooth muscle cell identity (*Acta2*, *Tagln*). j, Axon guidance gene expression (also termed as vascular guidance genes) are increased in Control EPDCs as compared to Myocardial Cells and decreased in MRTF depleted EPDCs based on FPKM values from bulk RNA-seq. For data in d-i, n=3 Control EPDCs and MRTF^{epiDKO} EPDCs. For data in j, n=3 non-EPDCs, Control EPDCs, and MRTF^{epiDKO} EPDCs. Bar graph data are presented as mean values +/- SEM. Statistical significance was determined by Two-sample unpaired student's t-test. ND, not-detected.

Supplementary Figure 10

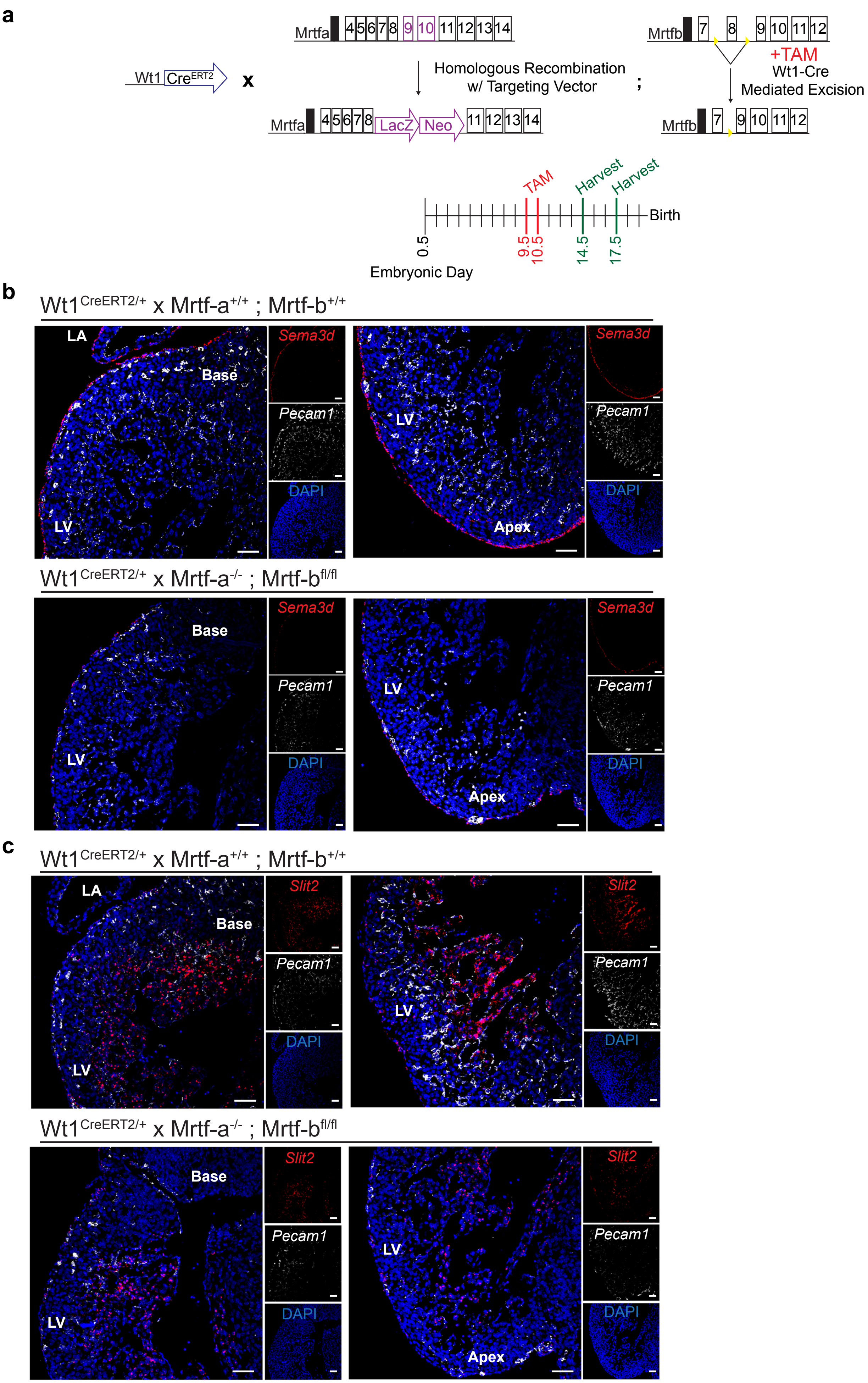

Supplementary Figure 10. In situ analysis of vascular guidance cues during embryonic development. a, Schematic representation of the strategy to delete *Mrtfs* in *Wt1* epicardial lineage cells. Tamoxifen (TAM) was administered to pregnant dams at embryonic day (E)9.5 and E10.5 and embryos were harvested at E14.5 and E17.5. In situ analysis of Sema3d and Slit2 (red) mRNA in b, Control and c, *Mrtf-a;Mrtf-b* double knockout ($MRTF^{epiDKO}$) hearts relative to the detection of *Pecam1*$^{+}$ (white) vascular cells (broad marker for endothelial cells) using RNAscope technology. Scale bar, 50µm. DAPI (4',6-diamidino-2-phenylindole) staining was utilized to visualize nuclei (blue). Immunostaining was repeated independently 3 times with similar results. LA, left atrium; LV, left ventricle.

Supplementary Figure 11

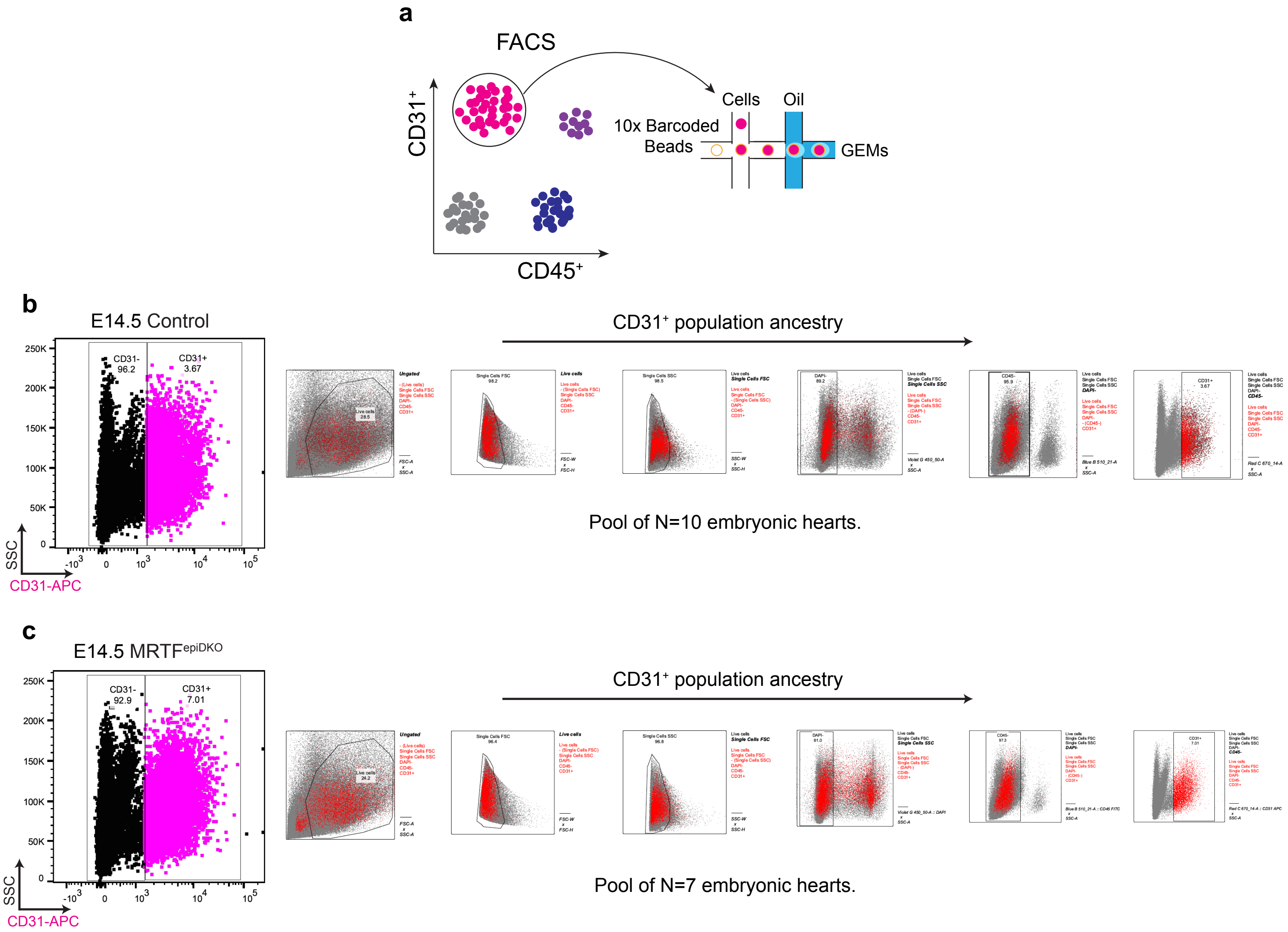

Supplementary Figure 11. Labeling and acquisition of endothelial cells during embryonic development for single cell RNA-sequencing. a, Enrichment of CD31$^+$/CD45$^-$ endothelial cells by fluorescence activated cell sorting (FACS) prior to single cell capture using 10x Genomics Chromium Controller. b and c, Flow cytometric analysis and strategy to enrich for CD31$^+$ endothelial cells (ECs) from whole embryonic hearts at E14.5 from (b) Control (n=10 hearts) and (c) *Mrtf-a*;*Mrtf-b* double knockout (MRTF^{epiDKO}) (n=7 hearts) animals. FACS sequential gating strategy was identical for ECs collected from Control and MRTF^{epiDKO} hearts. First, single cells were selected based on FSC-A and SSC-A, FSC-W x FSC-H, and SSC-W x SSC-H. Then, single and viable cells were selected based on a negative DAPI stain. Elimination of CD45 positive hematopoietic-derived cells from the sort was performed by selecting the CD45 negative population, followed by a positive selection of CD31 positive endothelial cells from the CD45 negative population. ECs were subsequently sorted into Eppendorf tubes and submitted for single-cell capture using the 10x Genomics Chromium Platform prior to single cell RNA-sequencing.

Supplementary Figure 12

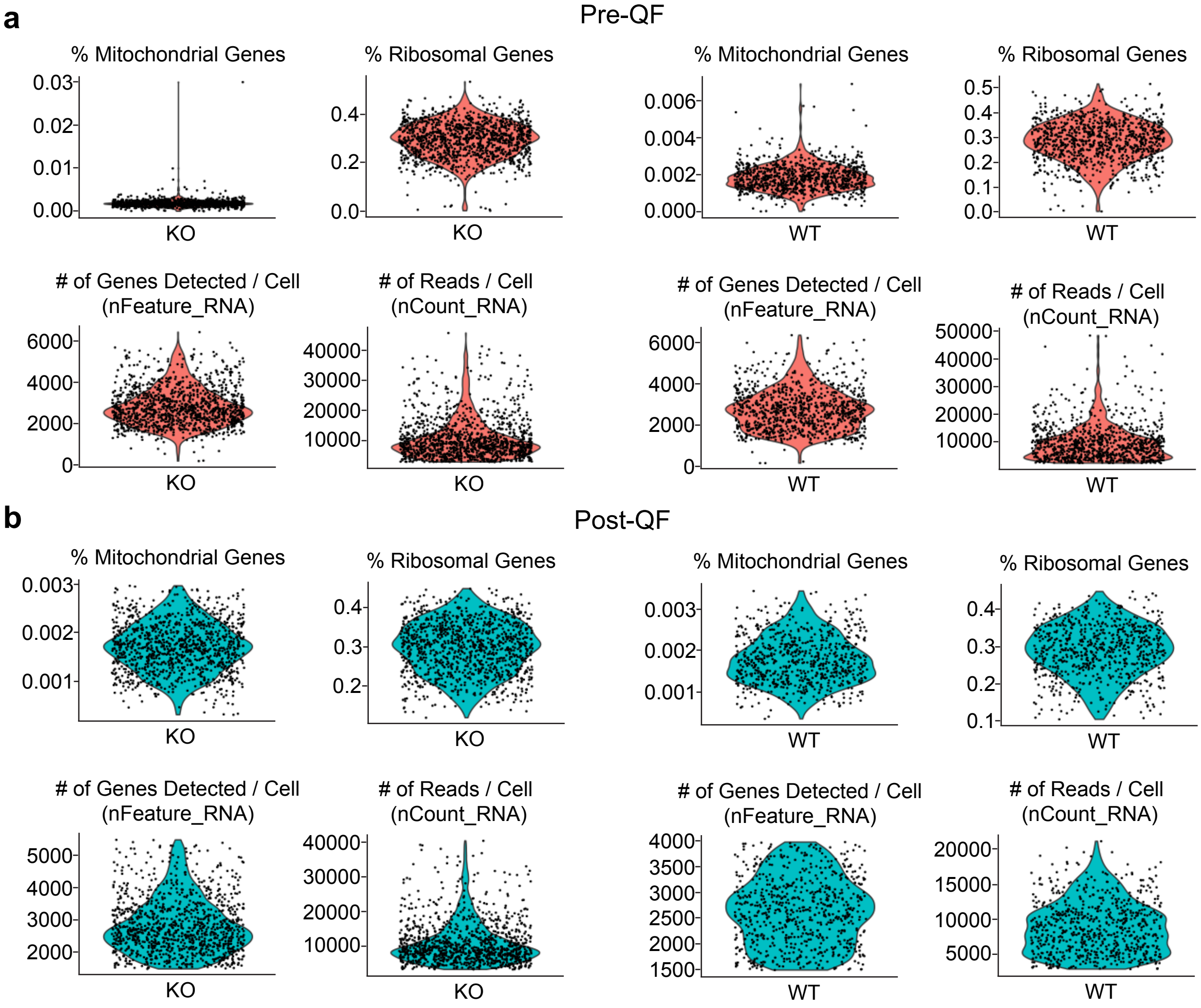

Supplementary Figure 12. Single cell RNA-sequencing quality control of endothelial cells. a and b, The percentage (%) of mitochondrial and ribosomal reads, number (#) of genes detected per cell (nFeature_RNA), # of reads per cell (nCount_RNA) were analyzed for quality filtering and presented as Pre-Quality Filtering (Pre-QF) versus Post-QF.

Supplementary Figure 13

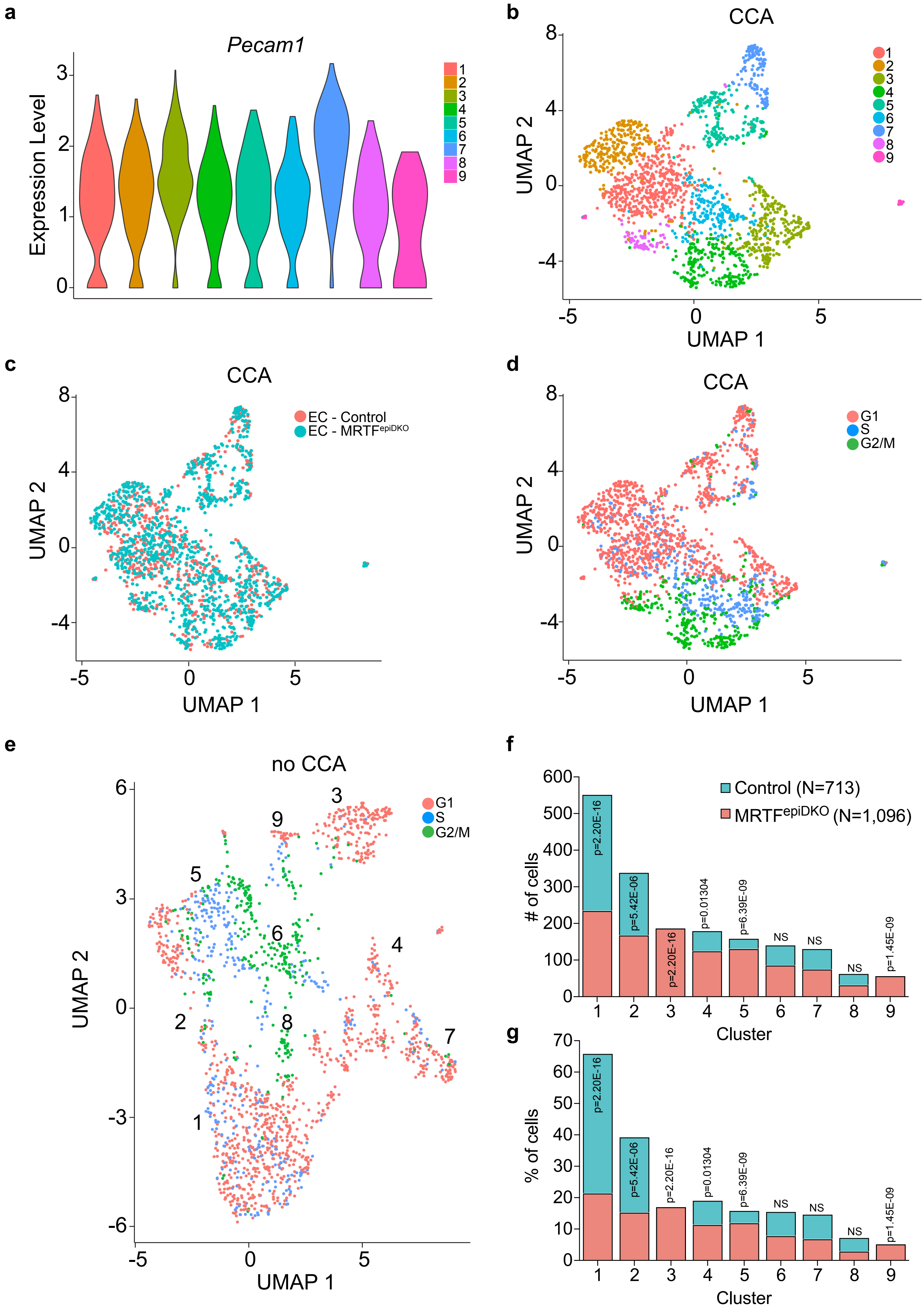

Supplementary Figure 13. Canonical Correlation Analysis (CCA) of endothelial cells acquired from control and MRTF^{epiDKO} hearts and cellular contribution to cell cycle activity and identity. a, Violin plots showing expression of *Pecam1* (platelet endothelial cell adhesion molecule 1) in 9 cell clusters. b-d, Identification of endothelial cell (EC) b, cell identities, c, represented by genotype and d, stage of cell cycle activity after performing a CCA of Control and *Mrtf-a;Mrtf-b* double knockout (MRTF^{epiDKO}) ECs. e, Uniform manifold approximation projection (UMAP) clustering of non-CCA EC clusters by cell cycle activity. f, Numbers of cells and g, the percentage (%) of cells in each cluster from either Control (713 cells) and MRTF^{epiDKO} (1,096 cells) mice. Two-sample student's t-test was performed to determine significance of the proportion of cells in single-cell clusters. NS, not-significant.

Supplementary Figure 14

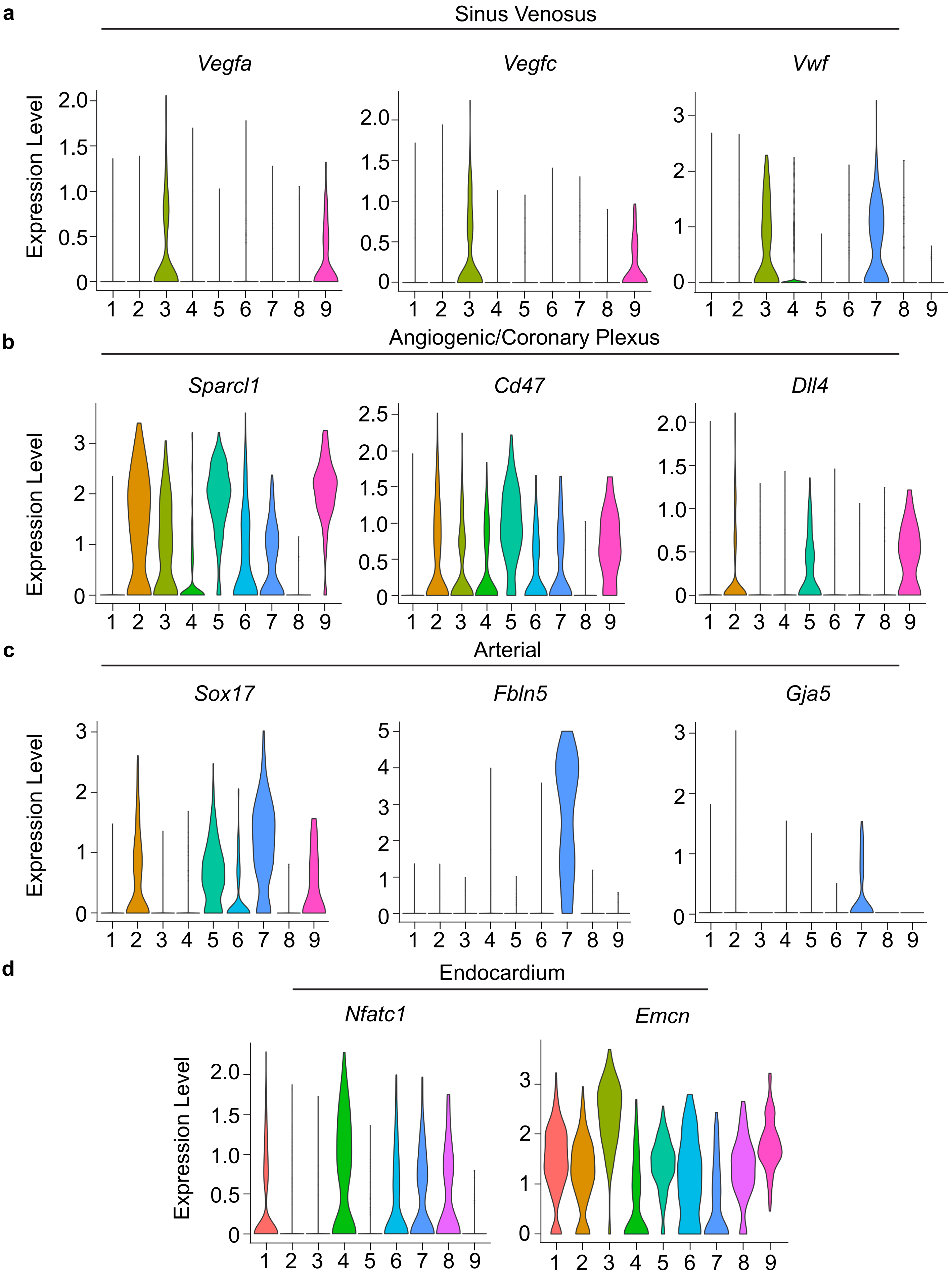

Supplementary Figure 14. Gene expression profiling of embryonic vascular cells. a-d. Violin plots showing expression of selected genes across 9 endothelial cell clusters to demonstrate cell identities associated with cells as (a) sinus venosus, (b) angiogenic/coronary plexus, (c) arterial or (d) endocardial.

Supplementary Figure 15

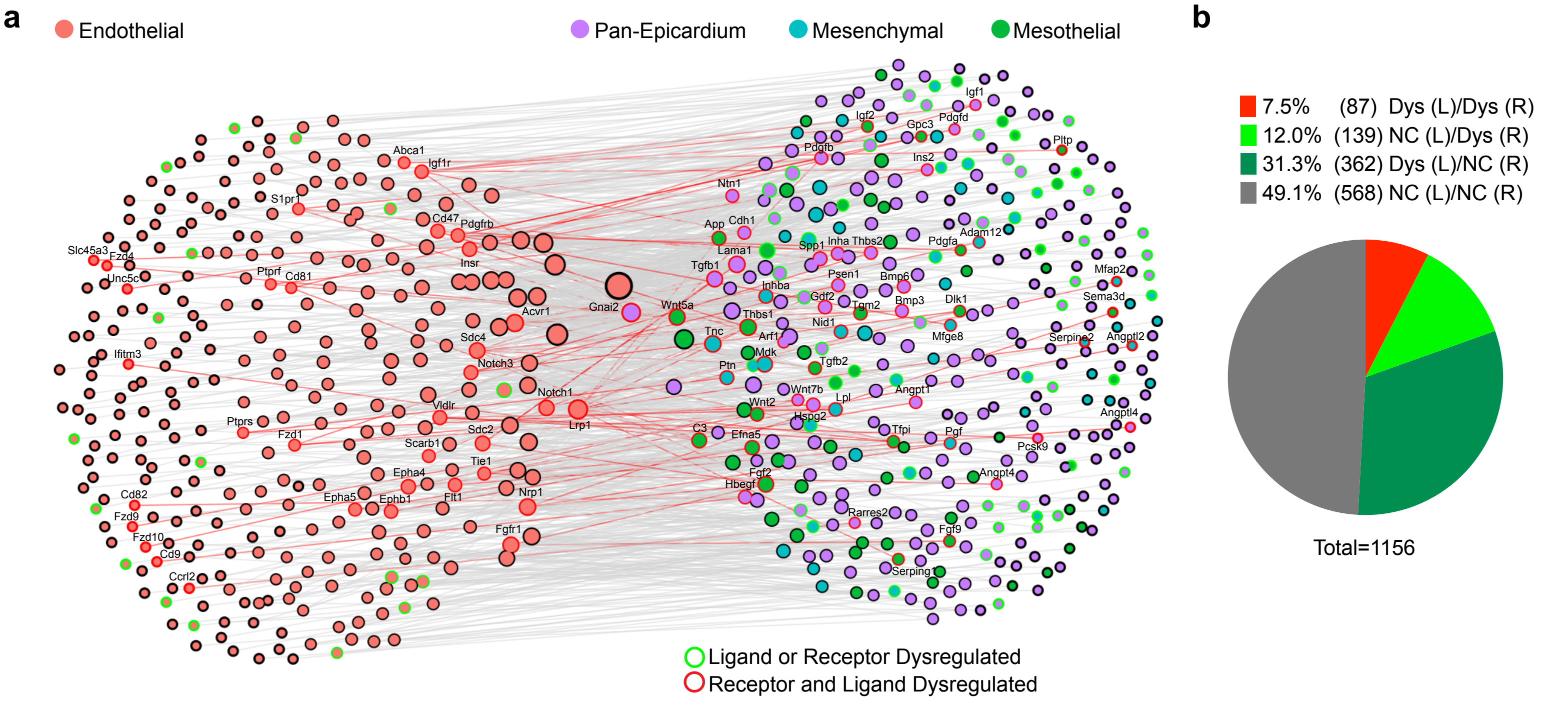

Supplementary Figure 15. Correlation expression network of epicardial cell expressing ligands and endothelial cell expressed receptors. a, Epicardial-derived cell - endothelial cell (EPDC-EC) interactome represented by ligand-receptor pairings. Shade of EPDC indicates whether ligand is broadly expressed or enriched in epicardium-derived mesenchyme or mesothelium. Green outline indicates dysregulation of either ligand or receptor within a pair, and red outline and red connecting line indicates both the receptor and ligand of a pair is disrupted. b, The proportion (%) of ligands (L) or receptors (R), which are dysregulated (Dys) or not-changed (NC).

Supplementary Figure 16

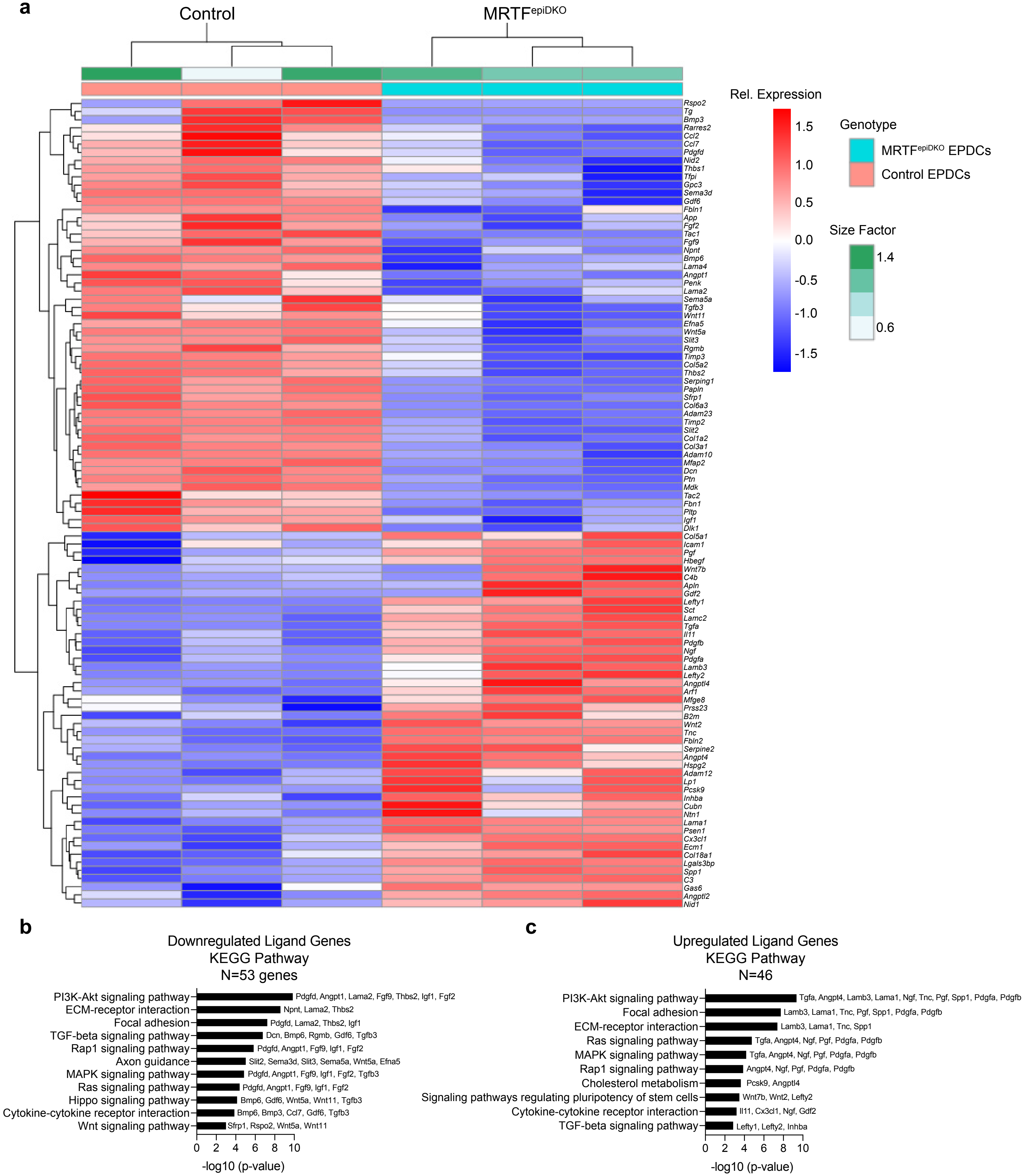

Supplementary Figure 16. Differentially expressed ligand genes in epicardial cells analyzed by bulk RNA-sequencing. a, Heat map of expression of 99 differentially expressed ligand genes in epicardium-derived cells (EPDCs) analyzed from Control and *Mrtf-a*;*Mrtf-b* double knockout (MRTF^{epiDKO}) mice. Scale represents relative (Rel.) expression values. b and c, Kyoto Encyclopedia of Genes and Genomes (KEGG) Pathway analysis of down (53) and up-regulated (46) ligand genes in MRTF^{epiDKO} EPDCs represented as -log10 (p-value). GO term enrichment significance was determined using Fisher exact test.

Supplementary Figure 17

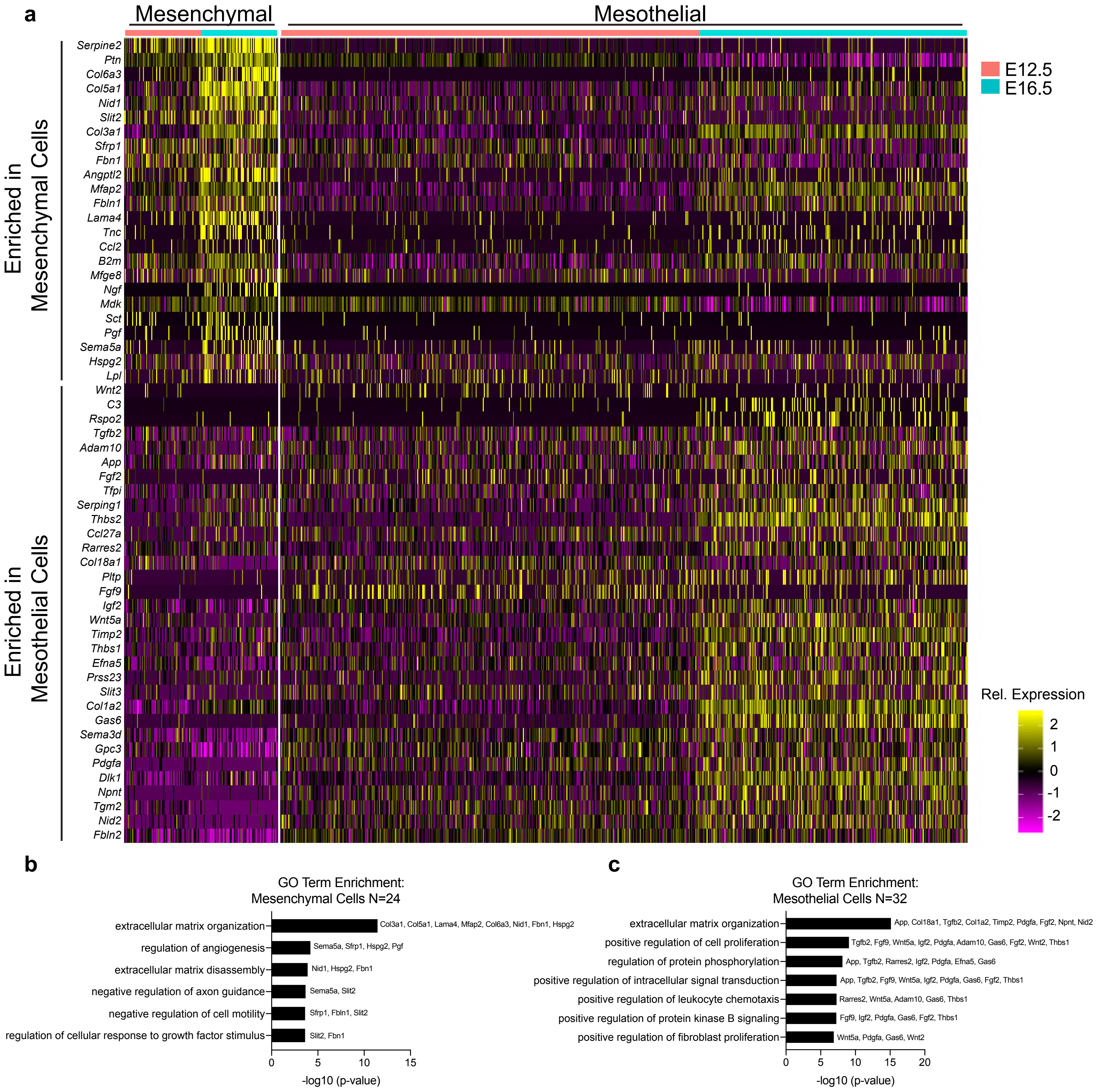

Supplementary Figure 17. Differentially expressed ligand genes in epicardial cells analyzed by single cell RNA-sequencing. a, Heat map presentation of expressed genes in epicardium-derived cells (EPDCs) termed as mesenchymal (Cluster 5-7) or mesothelial (Cluster 1-4) regardless of developmental age. b and c, Gene ontology (GO) term enrichment of the expressed ligands significantly upregulated in mesenchymal or mesothelial cells categorized by single cell RNA-seq and represented -log10 (p-value). GO term enrichment significance was determined using Fisher exact test.

Supplementary Figure 18

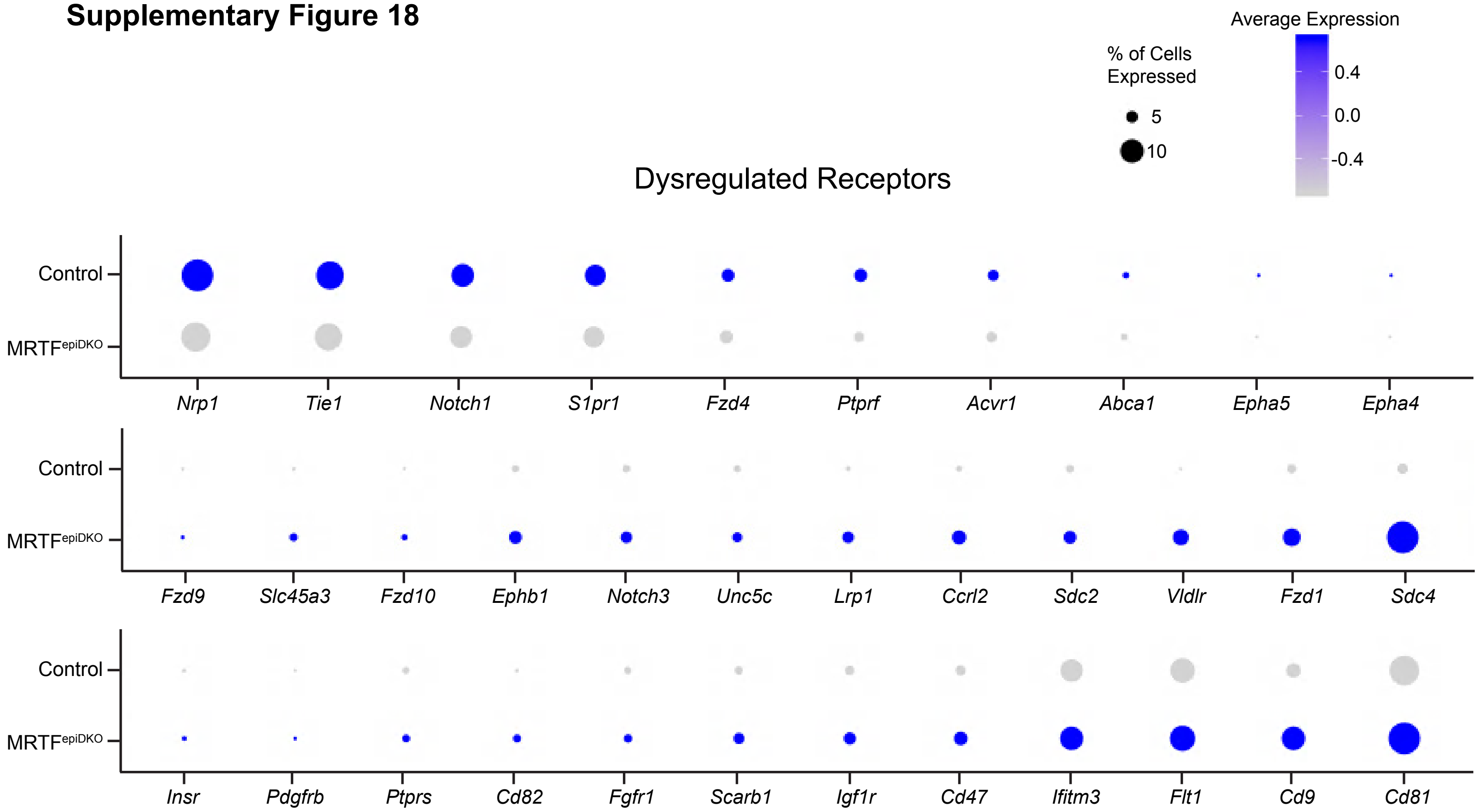

Supplementary Figure 18. Differentially expressed receptor genes in endothelial cells following knockout of *Mrtfs* in the epicardium. Dot plot expression of the significant dysregulated receptors in ECs isolated from Control or *Mrtf-a*;*Mrtf-b* double knockout (MRTF^{epiDKO}) hearts. Color of cells represents average expression levels as compared to other clusters and the size of dot represents the % of cells expressing the particular gene.

Supplementary Figure 19

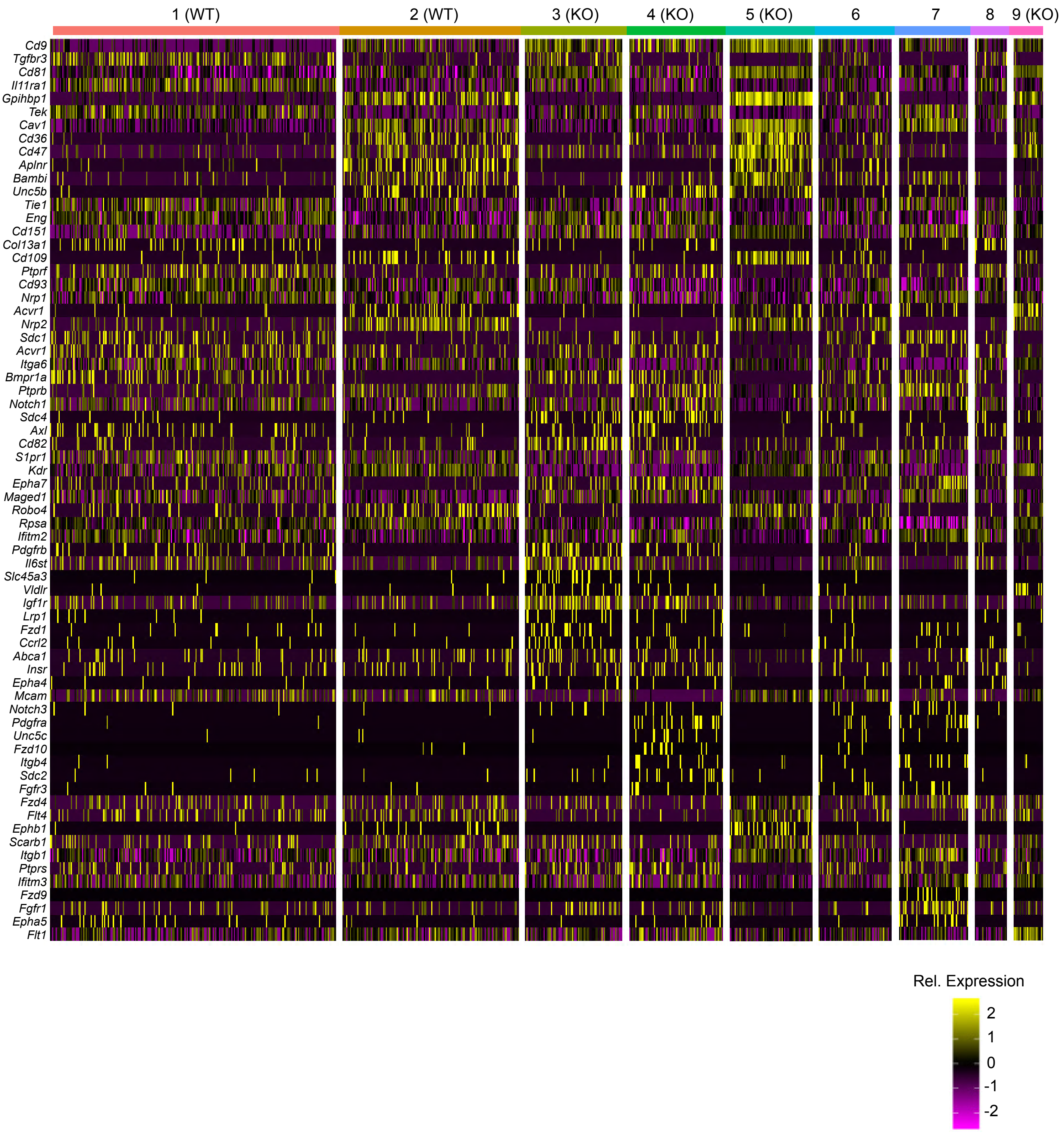

Supplementary Figure 19. Differentially expressed receptor genes across all 9 endothelial cell clusters. Heat map representation of the 68 dysregulated receptors in each endothelial cell cluster and isolated from Control and *Mrtf-a*;*Mrtf-b* double knockout (MRTF^{epiDKO}) hearts. Scale represents relative (Rel.) expression values.

Supplementary Figure 20

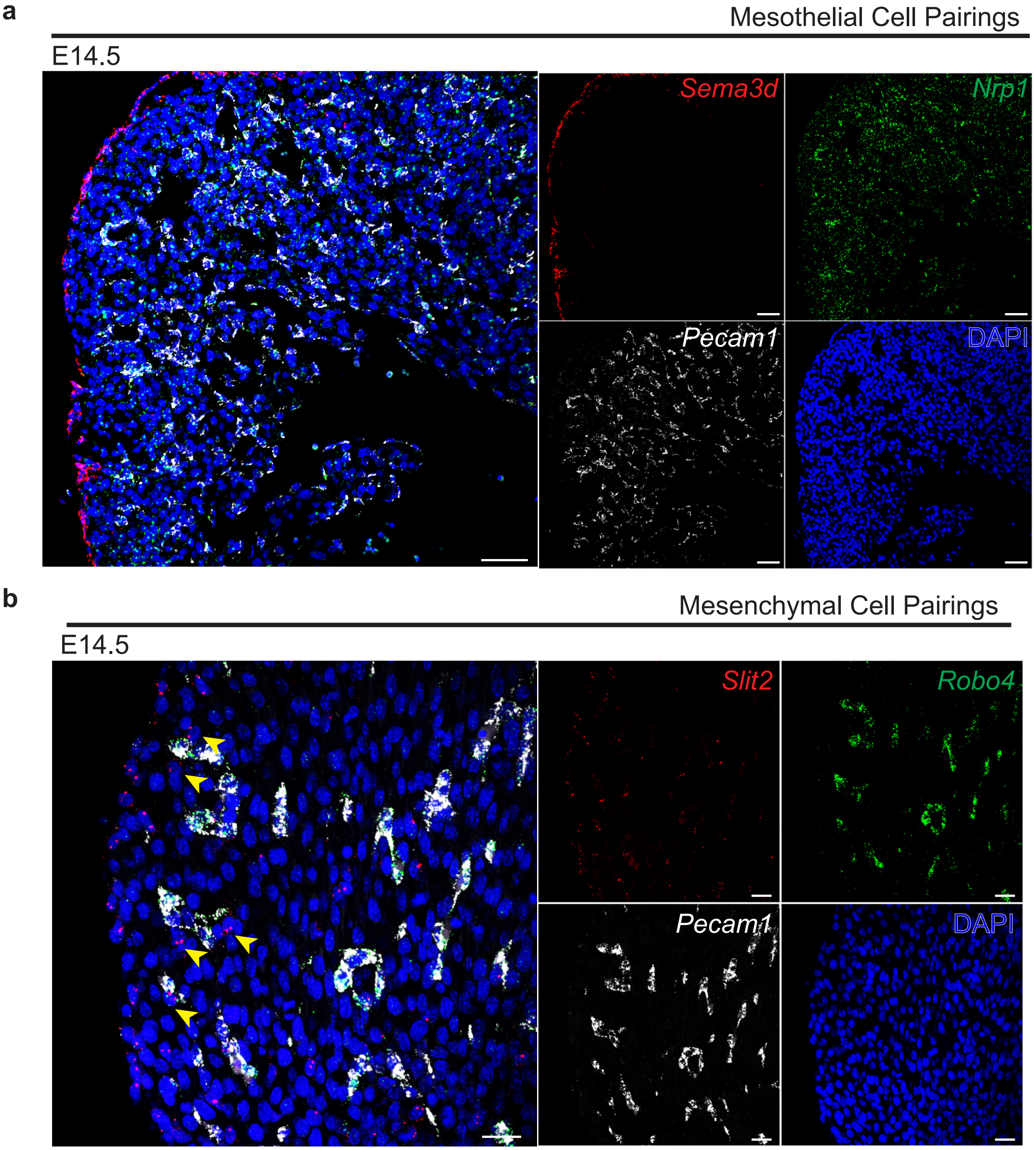

Supplementary Figure 20. Low magnification in situ analysis of ligand-receptor pairs during cardiac development. a, Fluorescence in situ hybridization (FISH) using probes against endothelial cell receptor gene *Nrp1* (green) and epicardial cell ligand gene *Sema3d* (red) at embryonic day (E)14.5. Scale bar, 50μm. b, FISH probes against endothelial cell receptor gene *Robo4* (green) and epicardial cell ligand gene *Slit2* (red) at E14.5. Yellow arrowheads, *Slit2*$^{+}$ cells near *Robo4*$^{+}$ cells. Scale bar, 25μm. a and b, a probe against *Pecam1* (white) was used to detect endothelial cells broadly. DAPI (4',6-diamidino-2-phenylindole) staining was utilized to visualize nuclei (blue). Immunostaining was repeated independently 3 times with similar results.

Supplementary Figure 21

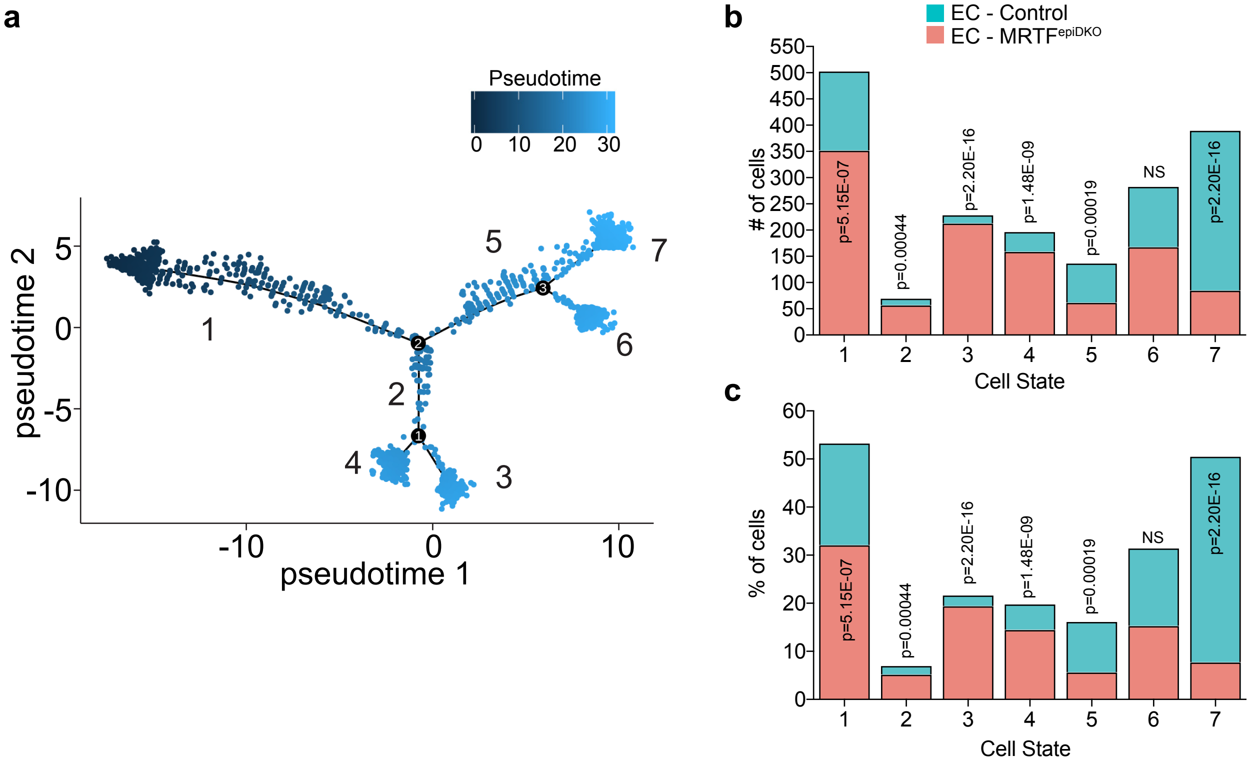

Supplementary Figure 21. Coordinated arteriovenous specification by epicardial epithelial-to-mesenchymal transition. a, Monocle generated pseudotime trajectory of endothelial cell (EC) development. b, Number (#) of cells and c, percentage (%) of cells in each cell state. NS, not-significant. Two-sample student's t-test was performed to determine significance of the proportion of cells in single-cell clusters.

Supplementary Figure 22

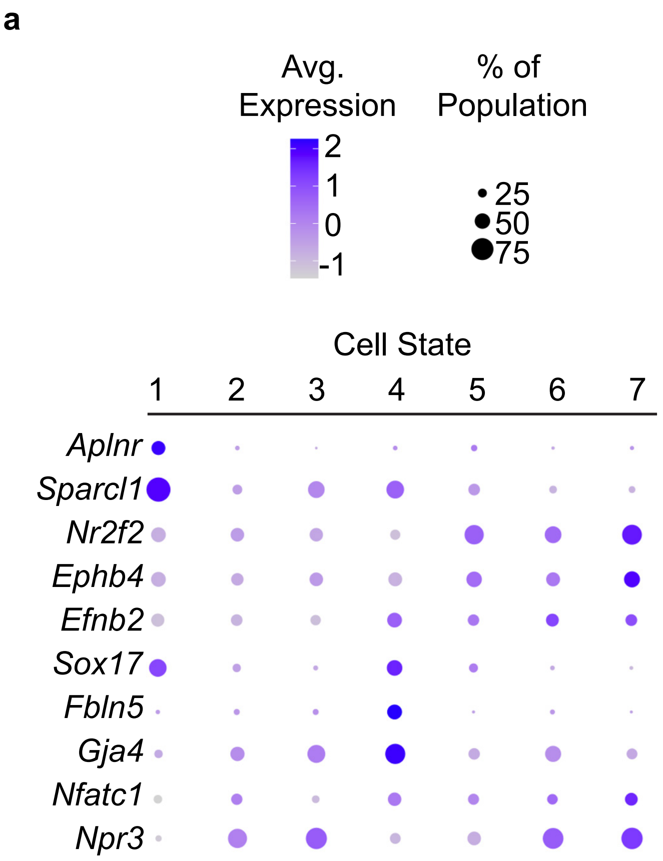

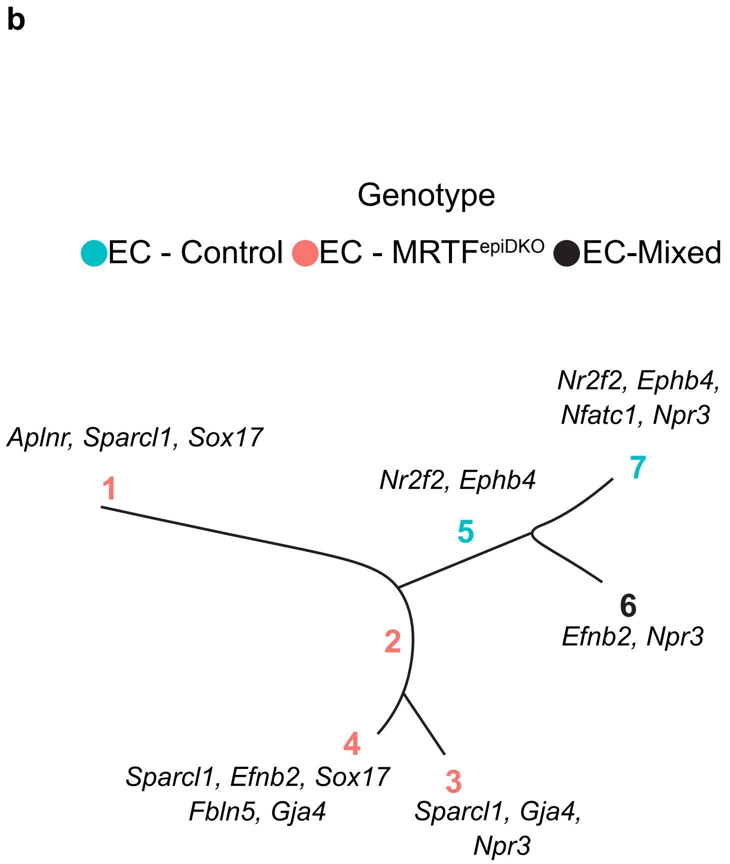

Supplementary Figure 22. Arteriovenous gene expression during pseudotime analysis. a, Dot plot expression of angiogenic, venous, arterial and endocardial markers in ECs from Control or MRTF^{epiDKO} hearts across pseudotime states (1-7). Color of cells represents average expression levels as compared to other clusters and the size of dot represents the % of cells expressing the particular gene. b, Schematic of enriched gene markers in the 7 pseudotime states. States are represented by ECs found to be from control, *Mrtf-a*;*Mrtf-b* double knockout (MRTF^{epiDKO}), or a mixture of both mouse genotypes.

Supplementary Figure 23

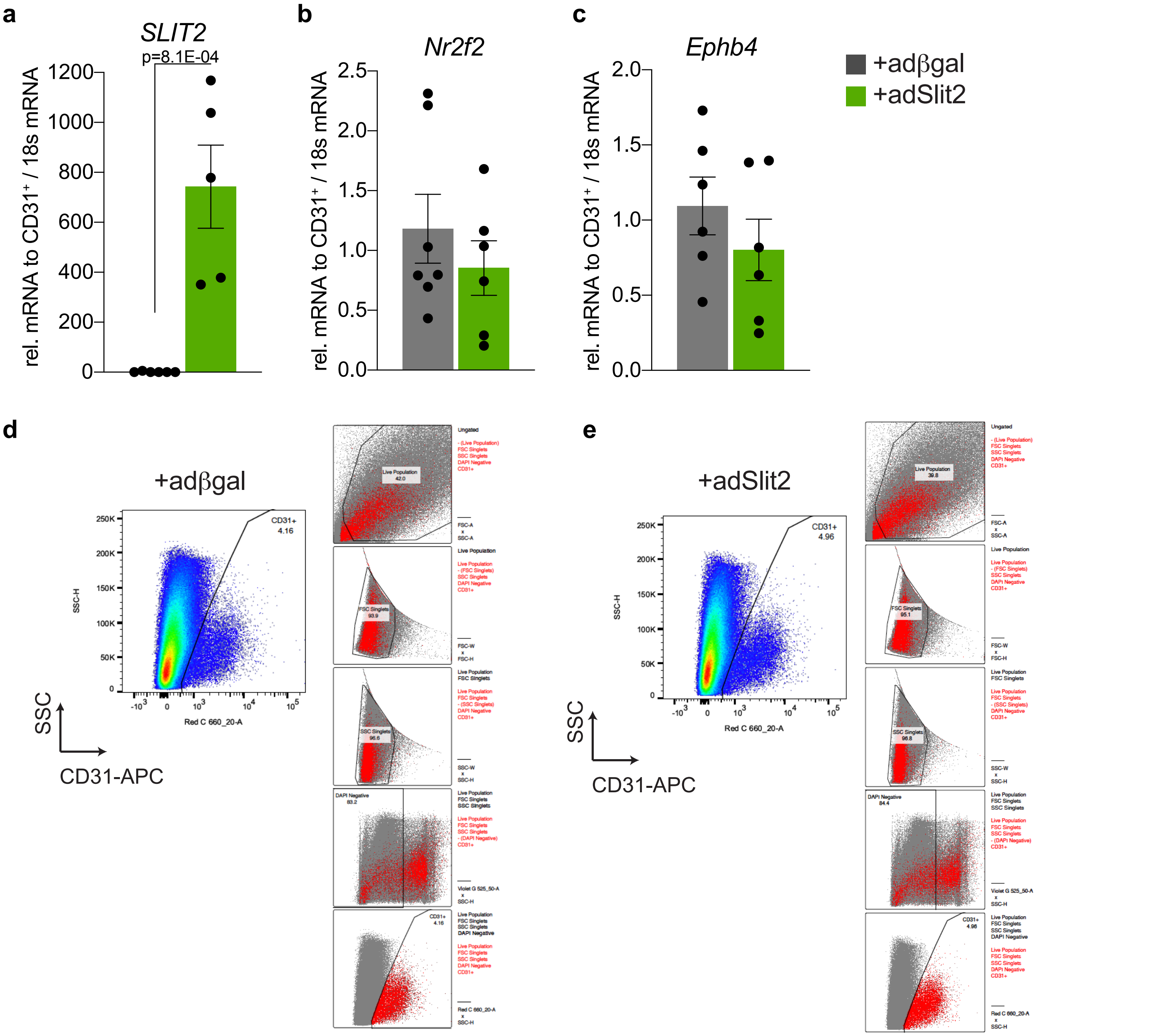

Supplementary Figure 23. SLIT2 expression in the epicardium alters endothelial cell specification. Gene expression represented as fold change relative to $CD31^{+}$ cells acquired from ad/βgal treated hearts for a, *SLIT2*, b, *Nr2f2*, c, *Ephb4*. All n were analyzed as individual samples acquired from independent embryos and examined over 2 experiments. ad/β-gal n=6 *SLIT2* and *Ephb4* n=7 *Nr2f2* and ad/Slit2 n=5 *SLIT2* and n=6 *Nr2f2* and *Ephb4*. Data are presented as mean values +/- SEM. Statistical significance was determined by Two-sample unpaired student's t-test. d and e, FACS sequential gating strategy was identical for endothelial cells collected from ad/βgal and ad/Slit2 treated hearts. First, single cells were selected based on FSC-A and SSC-A, FSC-W x FSC-H, and SSC-W x SSC-H. Then, single and viable cells were selected based on a negative DAPI stain followed by a positive selection of CD31 positive endothelial cells.

Supplementary Figure 24

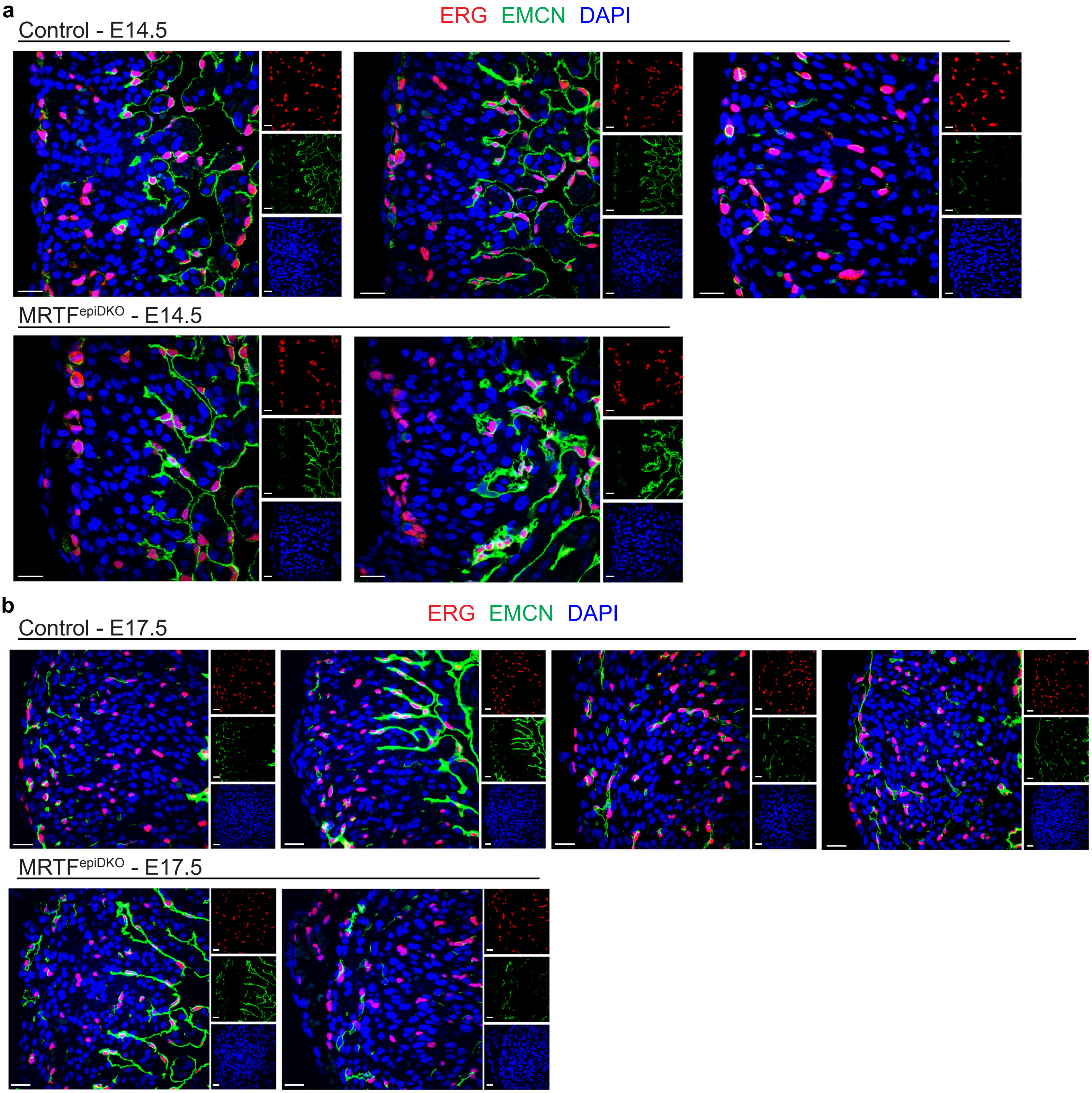

Supplementary Figure 24. Endothelial cell localization and polarization is disrupted after deletion of *Mrtfs* from the epicardium. Additional representative immunofluorescence staining of sections from hearts isolated at embryonic stage a, (E)14.5 and b, E17.5 with antibodies directed against ERG (red, pan-EC) and EMCN (green, venous and endocardial EC) in Control and *Mrtf-a*;*Mrtf-b* double knockout (MRTF^{epiDKO}) hearts. Scale bar (a), 20μm and Scale bar (b), 25μm. DAPI (4',6-diamidino-2-phenylindole) staining was utilized to visualize nuclei (blue). Immunostaining was repeated independently 2 times with similar results.

Supplementary Figure 25

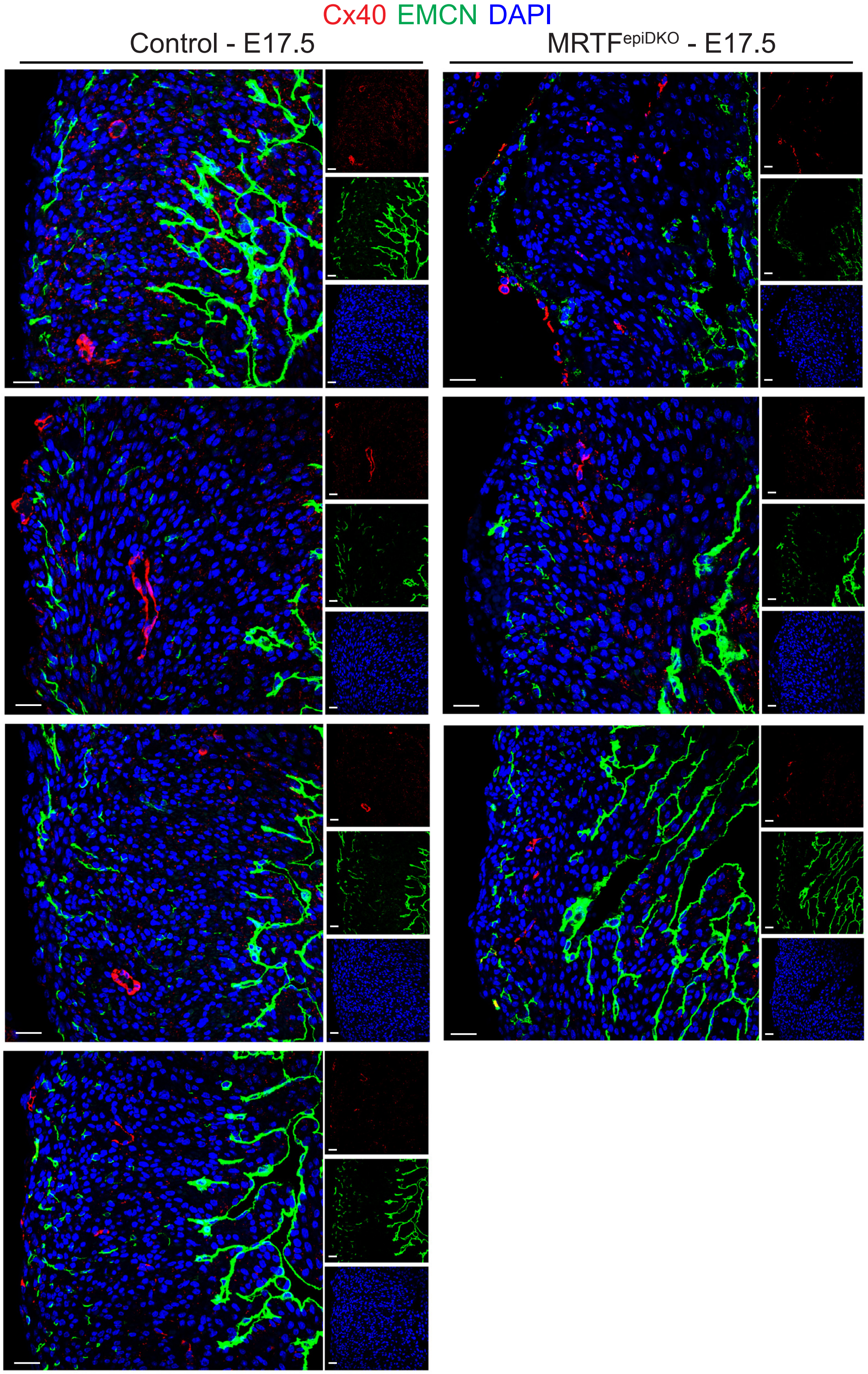

Supplementary Figure 25. Endothelial cell localization and polarization is disrupted after deletion of *Mrtfs* from the epicardium. Additional representative immunofluorescence staining of sections from hearts isolated at embryonic stage (E)17.5 with antibodies directed against Cx40 (red, arterial) and EMCN (green, venous and endocardial EC) in Control and *Mrtf-a*;*Mrtf-b* double knockout (MRTF^{epiDKO}) hearts. Scale bar, 25μm. DAPI (4',6-diamidino-2-phenylindole) staining was utilized to visualize nuclei (blue). Immunostaining was repeated independently 2 times with similar results.
