# Supplementary material for: Coordination of endothelial cell positioning and fate specification by the epicardium
Source: Nat Commun. 2021 Jul 6;12:4155. doi: 10.1038/s41467-021-24414-z (PMC8260743; doi:10.1038/s41467-021-24414-z)
Supplement: Supplementary file 2 — Description of Additional Supplementary Files [file 41467_2021_24414_MOESM2_ESM.docx]

Description of Additional Supplementary Files

Title: Supplementary Dataset 1.

Description: Differential expression analysis in epicardial cell clusters 1-8. Differential expression significance was determined using nonparametric Wilcoxon rank sum test.

Title: Supplementary Dataset 2.

Description: Differentially expressed genes in epicardial cells analyzed by pseudotime. Statistically significant pseudotime dependent genes was determined using student’s t-test.

Title: Supplementary Dataset 3.

Description: Differential gene expression in endothelial cells from control and MRTFepiDKO hearts following canonical correlation analysis. Differential expression significance was determined using non-parametric Wilcoxon rank sum test.

Title: Supplementary Dataset 4.

Description: Differential gene expression in endothelial cells from control and MRTFepiDKO hearts without canonical correlation analysis. Differential expression significance was determined using non-parametric Wilcoxon rank sum test.

Title: Supplementary Dataset 5.

Description: Differential gene expression in epicardial cells collected for bulk RNA-sequencing. Differential expression significance was determined using non-parametric Wilcoxon rank sum test.

Title: Supplementary Dataset 6.

Description: Ligandreceptor pairs between epicardial cells and endothelial cells during cardiac development.

Title: Supplementary Dataset 7.

Description: Cell cycle genes removed from endothelial cell monocle analysis.
