# Supplementary material for: Coordination of endothelial cell positioning and fate specification by the epicardium
Source: Nat Commun. 2021 Jul 6;12:4155. doi: 10.1038/s41467-021-24414-z (PMC8260743; doi:10.1038/s41467-021-24414-z)
Supplement: Supplementary file 10 — Reporting Summary [file 41467_2021_24414_MOESM10_ESM.pdf]

nature research

Corresponding author(s): Eric M. Small

Last updated by author(s): Jun 7, 2021

Reporting Summary

Nature Research wishes to improve the reproducibility of the work that we publish. This form provides structure for consistency and transparency in reporting. For further information on Nature Research policies, see our Editorial Policies and the Editorial Policy Checklist.

Statistics

For all statistical analyses, confirm that the following items are present in the figure legend, table legend, main text, or Methods section.

n/a	Confirmed	
☐	☒	The exact sample size (n) for each experimental group/condition, given as a discrete number and unit of measurement
☐	☒	A statement on whether measurements were taken from distinct samples or whether the same sample was measured repeatedly
☐	☒	The statistical test(s) used AND whether they are one- or two-sided<br>*Only common tests should be described solely by name; describe more complex techniques in the Methods section.*
☒	☐	A description of all covariates tested
☒	☐	A description of any assumptions or corrections, such as tests of normality and adjustment for multiple comparisons
☐	☒	A full description of the statistical parameters including central tendency (e.g. means) or other basic estimates (e.g. regression coefficient) AND variation (e.g. standard deviation) or associated estimates of uncertainty (e.g. confidence intervals)
☒	☐	For null hypothesis testing, the test statistic (e.g. F, t, r) with confidence intervals, effect sizes, degrees of freedom and P value noted<br>*Give P values as exact values whenever suitable.*
☒	☐	For Bayesian analysis, information on the choice of priors and Markov chain Monte Carlo settings
☒	☐	For hierarchical and complex designs, identification of the appropriate level for tests and full reporting of outcomes
☒	☐	Estimates of effect sizes (e.g. Cohen's d, Pearson's r), indicating how they were calculated

Our web collection on statistics for biologists contains articles on many of the points above.

Software and code

Policy information about availability of computer code

Data collection	All software used in this study is publicly available and previously published.<br><br>Single cell libraries were generated from epicardial cell and endothelial cells acquired by FACS. Prior to capture using the 10x Genomics Chromium controller (10x Genomics), the number of cells was quantitated (TC20 Automated Cell Counter, Bio-Rad) and cell viability was assessed via the trypan blue exclusion test of cell viability. Only cell populations exhibiting greater than 80% viability were used. All cells were loaded in order to maximize the number of single cells acquired using the Chromium single Cell 3' Reagent Kit. Libraries were prepared according to manufacturer's instructions using the Chromium Single Cell 3' Library and Gel Bead Kit v.2 (10x Genomics). Preliminary processing of raw sequencing reads. CellRanger v2.2.0 was used to demultiplex each capture, process base-call files to fastq format, and perform 3' gene counting for each individual cell barcode with mouse reference data set (mm10, v 2.1.0).<br><br>Library preparation and sequencing of epicardial cells (for bulk RNA-sequencing) was performed with Clontech Ultralow RNA Kit in conjunction with NexteraXT DNA Library Prep Kit (Illumina) was used for next generation sequencing library construction according to manufacturer's protocols. Briefly, mRNA was purified from 1ng total RNA with oligo-dT magnetic beads and fragmented. First-strand cDNA synthesis was performed with random hexamer priming followed by second-strand cDNA synthesis using dUTP incorporation for strand marking. End repair and 3` adenylation was then performed on the double stranded cDNA. Illumina adaptors were ligated to both ends of the cDNA, purified using Ampure beads and amplified with PCR primers specific to the adaptor sequences to generate cDNA amplicons of approximately 200-500bp in size. The amplified libraries were hybridized to the Illumina single end flow cell and amplified using the cBot (Illumina). Single end reads of 100nt were generated for each sample using Illumina's HiSeq2500v4. Raw reads were generated from Illumina HiSeq2500 sequencing and demultiplexed using bcl2fastq version 1.8.4.
Data analysis	Data analysis for this study utilized code built from publicly available and published tools. No custom/unpublished tools were utilized for data analysis in this study.<br>Single cell transcriptome sequencing of epicardial cells.

Quality control, identification of variable genes, principle component analysis and non-linear reduction using UMAP was performed using Seurat (v3.0.0.9000 and R v3.5.1) for each individual time point separately. The integration function RunCCA was utilized to identify cell type specific clusters without respect to developmental time.. Cell type annotations were identified based on significant cluster specific marker genes and the Mouse Gene Atlas using Enrichr (enrichR_2.1). In order to understand the effect of developmental time, the Seurat (v3.0.0.9150) function merge() was used to combined the E12.5 and E16.5 captures to maintain the variation introduced by developmental time. Cell cycle scoring was performed and the variation introduced as number of genes involved in mitochondrial transcription, and cell cycle phases S and G2/M were regressed out during data scaling. Data was visualized in UMAP space and clustered were defined using a resolution of 0.5.

Developmental trajectory and prediction of cell-fate determinants.
The GetAssayData() function in Seurat (v3.0.0.9150) was used to extract the raw counts to construct the Monocle object. To construct the trajectory the default functions and parameters as suggested by Monocle (v2.10.1) were used along with the following deviations: the hyper variable genes defined using Seurat VariableFeatures() function were used as the ordering genes in Monocle, 8 principle components were used for further non-linear reduction using tSNE, and num_clusters was set to 5 in the clusterCells() Monocle function. The resulting Monocle trajectory was colored based on Monocle State, Pseudotime, developmental origin (E12.5 or E16.5), and Seurat clusters previously identified. Genes that are dynamically expressed at the one identified branchpoint were analyzed using the BEAM() function. The top 50 genes that are differentially expressed at the branchpoint were visualized using the plot_genes_branched_heatmap() function in Monocle.

Integration with Mouse Cell Atlas.
Neonatal hearts from one day old pups were downloaded from the Mouse Cell Atlas (https://figshare.com/articles/MCA_DGE_Data/5435866) and re-analyzed using Seurat v3 following standard procedures previously outlined. Epicardial (E12.5 and E16.5) and neonatal-heart (1 day old) were integrated using the FindIntegegrationAnchors() and IntegrateData() functions using Seurat v3. Data were visualized in the 2-dimensional UMAP space. Marker genes were identified for the integrated clusters and Enrichr (enrichR_2.1) was used to identified significantly enriched Biological Processes (Gene Ontology 2018).

Single cell transcriptome sequencing of endothelial cells.
Cell filtering, cell-type clustering analysis and creation of cellular trajectories. Seurat (3.0.2) was used to filter low quality cells, score the cells by cell cycle, and integrate the E14.5 MRTFepiDKO and Control datasets using the merge function. Cells were clustered using the first 36 dimensions of PCA to resolution of 0.7 and visualized using UMAP. Monocle (2.10.1) was used to infer cellular trajectory. The determined cell states were used to determine cell state proportions of MRTFepiDKO and Control and identify potential markers for these cell states. Originating datasets, pseudotime states, and cell cycle state colorings were used within generated graphics.

Receptor-ligand pairing.
Using published lists of pairings from J.A. Ramilowski et. al. Nature Communications. 2015 (Ramilowski JA, Goldberg T, Harshbarger J, Kloppmann E, Lizio M, Satagopam VP, Itoh M, Kawaji H, Carninci P, Rost B and Forrest AR. A draft network of ligand-receptor-mediated multicellular signalling in human. Nat Commun. 2015;6:7866.), the receptor-ligand pairings were converted to MGI gene symbol from HGNC gene symbol using biomaRt (2.42.0). Ligands that were shown to be differentially expressed within the whole transcriptome sequencing of the MRTFepiDKO epicardial cells in comparison to the Control were flagged for later consideration. Both the endothelial and epicardial datasets were filtered for expressed receptors and ligands, respectively. Ligands expressed within the epicardial dataset were categorized as being differentially expressed between mesothelial and mesenchymal cell populations. Receptors expressed within the E14.5 MRTFepiDKO and Control combined dataset were characterized as differentially expressed between the two conditions. Seurat's DotPlot and doHeatMap functions were used to visualize differential expression across both datasets. For network visualization, tidyverse (1.3) was used for data analysis, viridis (0.5.1) (https://cran.r-project.org/web/packages/viridis/index.html) was used for color mapping, and both igraph (1.2.4.2) (https://igraph.org/) and ggraph (2.0.1) (https://cran.r-project.org/web/packages/ggraph/index.html) were used to generate and plot the network map. Epicardial ligands and endothelial receptors were grouped together and colored based on differential regulation; green if they were solely differentially regulated within that dataset or red if they had a corresponding differentially regulated ligand or receptor. Red-lines connect receptors and ligand pairs, which were both confirmed to be differentially expressed. The epicardial ligands were further colored by expression in specific cell populations identified as mesothelial, mesenchymal or general epicardial.

Whole transcriptome sequencing of epicardial cells.
The Clontech Ultralow RNA Kit in conjunction with NexteraXT DNA Library Prep Kit (Illumina) was used for next generation sequencing library construction according to manufacturer's protocols. Briefly, mRNA was purified from 1ng total RNA with oligo-dT magnetic beads and fragmented. First-strand cDNA synthesis was performed with random hexamer priming followed by second-strand cDNA synthesis using dUTP incorporation for strand marking. End repair and 3` adenylation was then performed on the double stranded cDNA. Illumina adaptors were ligated to both ends of the cDNA, purified using Ampure beads and amplified with PCR primers specific to the adaptor sequences to generate cDNA amplicons of approximately 200-500bp in size. The amplified libraries were hybridized to the Illumina single end flow cell and amplified using the cBot (Illumina). Single end reads of 100nt were generated for each sample using Illumina's HiSeq2500v4. Raw reads were generated from Illumina HiSeq2500 sequencing and demultiplexed using bcl2fastq version 1.8.4. Quality filtering and adapter removal were performed using Trimmomatic version 0.32 with the following parameters: "TRAILING:13 LEADING:13 ILLUMINACLIP:adapters.fasta:2:30:10 SLIDINGWINDOW:4:20 MINLEN:15". Processed/cleaned reads were then mapped to the GRCm38 reference genome using the SHRiMP version 2.2.3 and the following parameters: " --qv-offset 33 --all-contigs". Uniquely aligned and multi-mapped reads were counted within the gencode GRCm38 gene annotations, in a strand specific manner, using the cuffdiff tool from the cufflinks-2.0.2 package and the following parameters: "--FDR 0.05 -u -b GENOME". Differential expression analyses and data normalization were performed using DESeq2-1.14.1 R/ Bioconductor package with an adjusted p-value (Benjamini-Hochberg) threshold of 0.05 within the R version 3.3.1 environment (https://www.R-project.org). The PCA plot was created given the top500 genes with the most variation using the stats-3.4.0 (prcomp) and rgl-0.98.1 R packages. K-means clustering was performed on DEGs using log-transformed, normalized counts in Cluster 3.0 (http://bonsai.hgc.jp/~mdehoon/software/cluster/software.htm). Heat maps were generated using TreeView software (Version 1.1.6r4) and GraphPad (Version 8.4.2). Gene ontology analysis was performed using EnrichR and Ingenuity Pathway Analysis. Kyoto Encyclopedia of Genes and Genomes (KEGG) pathway analysis was performed using EnrichR.
Image J (Fiji Version: 2.0.0-rc-69/1.52p) was utilized to quantitate endothelial cell polarity and endothelial cell distance from the epicardial border.

For manuscripts utilizing custom algorithms or software that are central to the research but not yet described in published literature, software must be made available to editors and reviewers. We strongly encourage code deposition in a community repository (e.g. GitHub). See the Nature Research guidelines for submitting code & software for further information.

Data

Policy information about availability of data

All manuscripts must include a data availability statement. This statement should provide the following information, where applicable:
- Accession codes, unique identifiers, or web links for publicly available datasets
- A list of figures that have associated raw data
- A description of any restrictions on data availability

Bulk data of epicardial cells generated in this study have been deposited in the Gene Expression Omnibus database under accession code GSE153367 [https://www.ncbi.nlm.nih.gov/geo/query/acc.cgi?acc=GSE153367].

Single cell analysis of epicardial cells and endothelial cells data generated in this study have been deposited in the Gene Expression Omninubus database under accession code GSE154715 [https://www.ncbi.nlm.nih.gov/geo/query/acc.cgi?acc=GSE154715].

Figures with associated raw data: Main Figures 1, 2, and 4-7. Supplemental Figures 2-6, 8, 9, 12-19, 21, 22.

All RNA-sequencing data is available to the public.

Field-specific reporting

Please select the one below that is the best fit for your research. If you are not sure, read the appropriate sections before making your selection.

[x] Life sciences [ ] Behavioural & social sciences [ ] Ecological, evolutionary & environmental sciences

For a reference copy of the document with all sections, see nature.com/documents/nr-reporting-summary-flat.pdf

Life sciences study design

All studies must disclose on these points even when the disclosure is negative.

Sample size	Pre-determination of sample size for this study was not performed. Sample sizes were determined after breeding of our mouse strains. Although likelihood of certain genotypes in embryos could be predicted based on Mendelian ratios, factors such as: in utero tamoxifen toxicity, ex vivo heart culturing, and the use of MRTF mutant mouse lines impacted anticipated sample size/numbers.<br><br>Sample Size for Single Cell RNA-sequencing<br>Epicardium-derived cells (EPDCs) were collected from Wt1CreERT2/+ ; R26mTmG/+ embryos that were administered tamoxifen at E9.5 and E10.5 via pregnant dams. A total of 7 E12.5 staged hearts were pooled from 2 dams; and a total of 17 E16.5 staged hearts were pooled from 4 dams. Endothelial cells (ECs) were collected from Wt1CreERT2/+ mice (Control) and Wt1CreERT2/+ ; Mrtf-a-/- ; Mrtf-bflox/flox (MRTFepiDKO) after administration of tamoxifen at E9.5 and E10.5 via pregnant dams. A total of 10 Control hearts were pooled from 2 dams. A total of 7 MRTFepiDKO hearts were pooled from 2 dams. The number of timed-pregnancies required for the collection EPDCs and ECs was planned after performing pilot experiments to estimate the number of cells that could be collected per developmental stage or genotype.<br><br>Sample Size for Bulk RNA-sequencing of EPDCs<br>EPDCs were collected from Mrtf-a-/- ; Mrtf-bflox/flox and Srf-flox/flox hearts that were extracted at E12.5. N=3 Control EPDC, Control non-EPDC, and MRTFepiDKO EPDC were analyzed. N=2 SRFepiKO EPDC were analyzed. The number embryos required for the collection EPDCs for bulk RNA-sequencing was determined after performing pilot experiments, which were used to estimate the number of cells that could be collected for low-input RNA transcriptome analysis. Only embryos that yielded sufficient cell numbers following FACS were included in the bulk RNA-seq analysis. A N=3 between control and MRTFepiDKO was sufficient to reveal broad and significant transcriptional changes between EPDCs based on our analyses.<br><br>Sample sizes for gene expression analyses of primary cells as well as immunohistochemical and situ hybridization types of experiments were established based on the criteria listed above.
Data exclusions	An exclusion criteria of Pecam-1 gene expression was applied to the analysis of single-cell RNA sequencing data of endothelial cells due to the use of a conjugated CD31 antibody for collection of cells. Cells that did not express Pecam-1 were excluded from our analyses presented in Figures 6 and 7. Expression of Pecam-1 in populations of ECs is presented in Supplementary Figure 13a.
Replication	Replication of single cell or bulk RNA-sequencing was not performed. However, validation was conducted by qRT-PCR, immunohistochemistry, and FISH .
Randomization	Allocation of samples into experimental groups was not randomized as it was necessary to identify the exact genotypes of samples prior to performing experiments.<br><br>Allocation of treatments for in vitro studies of primary of epicardial cells (Figure 5a) was randomized.
Blinding	Experiments related to single cell and bulk RNA-sequencing were not blinded as the developmental age and/or genotype of tissue needed to be identified prior to performing transcriptomic data. Data presented in Figure 9b-e, 9g, h was blinded to the researcher. Sample names and groups were disclosed following quantitation and statistical analyses.

Reporting for specific materials, systems and methods

We require information from authors about some types of materials, experimental systems and methods used in many studies. Here, indicate whether each material, system or method listed is relevant to your study. If you are not sure if a list item applies to your research, read the appropriate section before selecting a response.

Materials & experimental systems

n/a	Involved in the study
☐	☒ Antibodies
☒	☐ Eukaryotic cell lines
☒	☐ Palaeontology and archaeology
☐	☒ Animals and other organisms
☒	☐ Human research participants
☒	☐ Clinical data
☒	☐ Dual use research of concern

Methods

n/a	Involved in the study
☒	☐ ChIP-seq
☐	☒ Flow cytometry
☒	☐ MRI-based neuroimaging

Antibodies

Antibodies used

Primary Antibodies
1. Rabbit anti-GFP (Green Fluorescent Protein). 1:200 dilution. Torrey Pines Biolabs, Inc. Catalog # TP401, Lot Number 071519.
2. Goat anti-PDGFRa. 1:100 dilution. R&D Systems, Catalog #AF1062.
3. Mouse anti-MYL2 (7C9). 1:50 dilution. Santa Cruz Biotechnology, Catalog #sc-517244.
4. Mouse anti-cTNT. 1:100 dilution. Fisher Scientific, Catalog #PIMA512960, Clone 13-11.
5. Rabbit anti-ERG (EPR3864). 1:200 dilution. Abcam, Catalog #ab92513.
6. Rat anti-EMCN. 1:100 dilution. Thermo Fisher Scientific, Catalog #14-5851-82, clone eBioV.7C7.
7. CD31 rat anti-mouse, APC. 1:100 dilution. BioLegend, Catalog #102410, clone 390, isotype Rat IgG2a, k.
8. CD45 Rat anti-Mouse, FITC. 1:50 dilution. BD Biosciences, Catalog #553079, clone 30-F11, isotype IgG2b, k.
9. Rabbit anti-CX40. 1:100 dilution. Alpha Diagnostic, Catalog #CX40-A.
10. Rabbit anti-HA-TAG. 1:100 dilution. Cell Signaling Technology, Catalog #3724S.

Secondary Antibodies
1. Donkey anti-Rabbit Biotin. 1:500 dilution. Jackson ImmunoResearch Laboratories, 711-065-152. Polyclonal.
2. Bovine anti-Goat HRP. 1:200 dilution. Jackson ImmunoResearch Laboratories, 805-035-180. Polyclonal.
3. Donkey anti-Rabbbit HRP. 1:200 dilution. Jackson ImmunoResearch Laboratories, 711-035-152. Polyclonal.
4. Streptavidin-555. 1:100 dilution. Thermofisher Scientific, S21381. Binds to biotinylated probe on sample.
5. Tyramide FITC. 1:200 dilution. Perkin Elmer, NEL741E001KT. Binds to horse-radish peroxidase (HRP) probe on sample.
6. Tyramide Cy3. 1:200 dilution. Perkin Elmer, NEL744E001KT. Binds to horse-radish peroxidase (HRP) probe on sample.
7. Donkey anti-Mouse 647. 1:200 dilution. Jackson ImmunoResearch Laboratories, 715-605-151. Polyclonal.
8. Donkey anti-Rat 488. 1:200 dilution. Jackson ImmunoResearch Laboratories, 703-545-155. Polyclonal.
9. Donkey anti-Rabbit 488. 1:100 dilution. Jackson ImmunoResearch Laboratories, 711-545-152. Polyclonal.

Validation

1. Rabbit anti-GFP . Antibody was used for immunohistochemistry, paraffin-embedded heart tissue. This antibody reacts with wild-type GFP and its variants EGFP. Validation of the GFP antibody was performed by co-staining tissue sections from genotypes that were Wt1+/+ ; R26mTmG/+ and Wt1CreERT2/+ ; R26mTmG/+. GFP-labeling was observed in Wt1CreERT2/+ ; R26mTmG/+ tissues and only after tamoxifen administration. Labeling is demonstrated in Figure 1c.
2. Goat anti-PDGFRa. Antibody was used for immunohistochemistry, paraffin-embedded heart tissue, with specific labeling to the plasma membrane of mesenchymal cells. PDGFRa has been validated to label the epicardium and cardiac mesenchymal cells during embryonic development (Smith CL, Baek ST, Sung CY and Tallquist MD. Epicardial-derived cell epithelial-to-mesenchymal transition and fate specification require PDGF receptor signaling. Circulation research. 2011;108:e15-26) as well as cardiac fibroblasts in the adult heart (Quijada P, Misra A, Velasquez LS, Burke RM, Lighthouse JK, Mickelsen DM, Dirkx RA, Jr. and Small EM. Pre-existing fibroblasts of epicardial origin are the primary source of pathological fibrosis in cardiac ischemia and aging. J Mol Cell Cardiol. 2019;129:92-104). Data shown in Supplementary Figure 1g and 1h demonstrates PDGFRa labeling of epicardial cells and interstitial mesenchymal cells at E12.5 and E16.5.
3. Mouse anti-MYL2 (7C9). Antibody was used for immunohistochemistry, paraffin-embedded heart tissue, and to label myosin regulatory light chain 2 a ventricular/cardiac muscle isoform. Data shown in Supplementary Figure 1g and 1h demonstrates MYL2 labeling of the nascent myocardium at E12.5 and E16.5, which is not detected in epicardium-derived cells or mesenchymal cells.
4. Mouse anti-cTNT. Antibody was used for immunohistochemistry, paraffin-embedded heart tissue, and to label the Troponin T Cardiac isoform in the myocardium. Data shown in Figure 4a demonstrates cTNT labeling of the nascent myocardium at E12.5 and is not detected in epicardium-derived cells.
5. Rabbit anti-ERG. Antibody was used for immunohistochemistry, paraffin-embedded heart tissue. ERG antibody was confirmed to be a novel and reliable marker of endothelial cells in the nervous system (Haber MA, Iranmahboob A, Thomas C, Liu M, Najjar A and Zagzag D. ERG is a novel and reliable marker for endothelial cells in central nervous system tumors. Clin Neuropathol. 2015;34:117-27) and and in the heart (Chang AH, Raftrey BC, D'Amato G, Surya VN, Poduri A, Chen HI, Goldstone AB, Woo J, Fuller GG, Dunn AR and Red-Horse K. DACH1 stimulates shear stress-guided endothelial cell migration and coronary artery growth through the CXCL12-CXCR4 signaling axis. Genes Dev. 2017;31:1308-1324). Additionally, we validated that ERG protein was specific to endothelial cells in embryonic hearts at E14.5 and E17.5 (as shown in Figure 9a and Supplementary Figure 24a, b) following co-

staining of endothelial cells with isolectin B4, which labels endothelial cells.
6. Rat anti-EMCN. Antibody was used for immunohistochemistry, paraffin-embedded heart tissue. Based on the manufacturer's target information, endomucin is also known as endothelial sialomucin. Endomucin protein can be detected in the superficial venous endothelial cells and in cells lining the endothelium (Cavallero S, Shen H, Yi C, Lien CL, Kumar SR and Sucov HM. CXCL12 Signaling Is Essential for Maturation of the Ventricular Coronary Endothelial Plexus and Establishment of Functional Coronary Circulation. Dev Cell. 2015;33:469-77). EMCN staining was reproduced and shown in Figure 9a, f and Supplementary Data Figure 24 and 25.
7. CD31 rat anti-mouse, APC. It is expressed on endothelial cells and hematopoietic cell derivatives. We demonstrated use of this antibody for enrichment of embryonic endothelial cells at E14.5. Collection of endothelial cells using FACS (Supplementary Figure 11a-c) followed by gene expression analysis confirmed enrichment of Pecam-1 and another canonical endothelial marker Cdh5 (VE-cadherin).
8. CD45 Rat anti-Mouse, FITC. This clone is reported to react with all isoforms and both alloantigens of CD45, which is commonly expressed in cells of hematopoietic origin. CD45 labeling was used exclude hematopoietic cells that co-express CD31 and enrich for cardiac endothelial cells at E14.5. Validation of this enrichment protocol was confirmed by gene expression analysis and cells were depleted of CD45 mRNA and enriched for Pecam-1 and Cdh5 mRNA.
9. Rabbit anti-CX40. Antibody was utilized for immunohistochemistry, paraffin-embedded heart tissue. The CX40 antibody was confirmed to be a consistent marker of endothelial cells that make up the coronary arteries. We validated the specific staining of CX40 to arterial endothelial cells by staining a time-course from E12.5 to E17.5. The CX40 antibody only labeled cells after E15.5, when mature coronary arteries are beginning to emerge. Additionally, this antibody was confirmed in a recent publication to be specific to arterial cells in the heart (Su T, Stanley G, Sinha R, D'Amato G, Das S, Rhee S, Chang AH, Poduri A, Raftrey B, Dinh TT, Roper WA, Li G, Quinn KE, Caron KM, Wu S, Miquerol L, Butcher EC, Irving Weissman I, Quake S, and Red-Horse K. Single cell analysis of early progenitor cells that build coronary arteries. Nature.2018 Jul;559(7714):356-362). CX40 antibody staining is shown in Figure 9f and Supplementary Figure 25.
10. Rabbit anti-HA TAG. Antibody was utilized for immunohistochemistry, paraffin-embedded heart tissue. According to the manufacturer's information, the HA-TAG antibody may be used for western blot, flow cytometry, immunohistochemistry and immunocytochemistry. In a separate study, the HA-TAG antibody validated SLIT2-HA overexpression after infecting HEK293 with the SLIT2-HA adenovirus and performing immunoblotting. HA-TAG antibody labeling to detect SLIT overexpression in the heart can be viewed in Figure 8b.

Animals and other organisms

Policy information about studies involving animals; ARRIVE guidelines recommended for reporting animal research

Laboratory animals

Laboratory mouse models were used in this study the following strains were used (all backcrossed onto C57BL/6J strain):
Wt1CreERT2/+ male X R26mTmG/+ female (E12.5 and E16.5)
Wt1CreERT2/+ male X R26tdTomato/+ female (E14.5)
Wt1CreERT2/+ male X C57BL/6 female (E14.5)
Wt1CreERT2/+; Mrtf-a-/- ; Mrtf-bflox/flox male X Mrtf-a+/- ; Mrtf-bflox/flox female (E14.5)
C57BL/6 (male X female) (E12.5)
Mrtf-a-/- ; Mrtf-bflox/flox male X Mrtf-a-/- ; Mrtf-bflox/flox female (E12.5)
Wt1CreERT2/+ male X Srfflox/flox female (E12.5)
Cspg4CreERT2/+ male X R26mTmG/+ female (E17.5)

Age of breeder male mice in this study ranged between 2-months of age to 12-months of age. Age of breeder female mice in this study ranged between 2-months of age to 6-months of age.

The sex of embryos was not determined.

Wild animals

This study did not involve wild animals.

Field-collected samples

This study did not involve samples collected from the field.

Ethics oversight

All animal experiments in this study were approved and performed under the guidance of the University Committee on Animal Resources at the University of Rochester.

Note that full information on the approval of the study protocol must also be provided in the manuscript.

Flow Cytometry

Plots

Confirm that:

- [x] The axis labels state the marker and fluorochrome used (e.g. CD4-FITC).
- [x] The axis scales are clearly visible. Include numbers along axes only for bottom left plot of group (a 'group' is an analysis of identical markers).
- [x] All plots are contour plots with outliers or pseudocolor plots.
- [x] A numerical value for number of cells or percentage (with statistics) is provided.

Methodology

Sample preparation

Cell isolation protocols for transcriptome profiling.

Epicardium-derived cells (EPDCs) and endothelial cells (ECs) were isolated from developmentally staged hearts as determined by the experimental strategy detailed. On the day of isolation, pregnant dams were anesthetized with an intraperitoneal injection of 0.5mL of ketamine-xylazine cocktail (13mg/mL ketamine in 0.88 mg/mL xylazine in DPBS) followed by cervical dislocation. After use of 70% ethanol to sterilize the abdominal area, an incision to enter and remove decidua away from the mesometrium was performed, and embryos were placed in pre-warmed HBSS (Thermo Fisher Scientific, SH30031.02). After the removal of extraembryonic tissue and the yolk sac, the hearts were removed from the embryo and placed in cell culture wells containing culture media made up of a 1:1 ratio of DMEM (Thermo Fisher Scientific, SH30022.FS) to M199 (Thermo Fisher Scientific, SH3025301) supplemented with 10% FBS (Gemini Bio-Products, 100106) and 1% Penicillin/Streptomycin (Pen-Strep; Thermo Fisher Scientific, SV30010). Digestion of embryonic hearts began by removing residual HBSS from wells and replacing media with a digestion solution containing 0.125% Collagenase IV (Millipore Sigma, C5138), 0.0125% Trypsin Protease (Thermo Fisher Scientific, SH30042.01), 1% chicken serum (Vector Laboratories, S-3000) diluted in pre-warmed HBSS before placing hearts in a 37°C hybridization oven with gentle shaking for 5 minute intervals. Following incubation, hearts were dissociated by gentle pipetting (3 times with a P1000 pipette) and undigested tissue was allowed to settle for 30 seconds. After settlement of tissue, media was collected and added to a separate tube containing horse serum (Vector Laboratories, S-2000) to neutralize digestion and digested cells were then saved on ice. Digestion, pipetting and collection of media was repeated 3 more times, and cells were then filtered through a 70um filter and centrifuged at 200g for 5 minutes at 4°C. The resulting pellet was placed in 10% FBS in DMEM (without phenol red, Thermo Fisher Scientific, SH30284.01) and saved on ice before performing fluorescence activated cell sorting FACS using a BD FACS Aria II (BD Biosciences). DAPI (4',6-Diamidino-2-Phenylindole, Dihydrochloride) was added to cells immediately before sorting (0.5ug/mL; Thermo Fisher Scientific, D1306) and to exclude dead cells. Cells were sorted directly in to 1.5mL Eppendorf tubes containing 0.5% bovine serum albumin (BSA, Millipore Sigma, A9647) in DPBS at 4°C and immediately processed.

Cell isolation of epicardial cells at E12.5 and E16.5 for scRNA-seq.
EPDCs were collected from Wt1CreERT2/+ ; R26mTmG/+ embryos that were administered 4-OHT at E9.5 and E10.5 via pregnant dams. A total of 7 E12.5 staged hearts were pooled from 2 dams; and a total of 17 E16.5 staged hearts were pooled from 4 dams based on visual confirmation of green fluorescent protein (GFP) expression in the epicardium using a Zoe Fluorescent Cell Imager (Bio-rad). Hearts negative for the expression of the Wt1CreERT2 allele, exhibited tdTomato fluorescence alone and were either discarded or used as tdTomato positive fluorescence controls for flow cytometry. Developmentally timed C57BL/6J embryos were collected as non-fluorescence controls for flow cytometry as well. Additionally, genomic DNA was isolated from all embryos and Wt1CreERT2 ; R26mTmG/+ positive embryos were confirmed using transgene specific primers followed by PCR (genotyping). Following the digestion protocol described, EPDCs were gated as single cells (based on FSC x SSC dimensions), DAPI negative, tdTomato negative and GFP positive. TdTomato positive cells were sorted for downstream gene expression analysis. EPDCs collected by FACS were immediately processed for single cell capture, library preparation and sequencing.

Cell isolation of epicardial cells at E12.5, E14.5 and E16.5 for cytokine gene expression analysis.
EPDCs were collected from both Wt1CreERT2/+ ; R26mTmG/+ and Wt1CreERT2/+ ; R26tdTomato/+ embryos that were administered 4-OHT at E9.5 and E10.5 via pregnant dams. Fluorescence was confirmed using the Zoe Fluorescent Cell Imager (Bio-Rad). Hearts negative for the expression of the Wt1CreERT2 allele, exhibited tdTomato fluorescence (R26mTmG/+) or were non-fluorescent (R26tdTomato/+) and were either discarded or used as fluorescence controls for flow cytometry. Following the digestion protocol described, EPDCs were gated as single cells (based on FSC x SSC dimensions), DAPI negative, tdTomato negative and GFP positive if cross was to the R26mTmG fluorescent reporter. If the R26tdTomato fluorescent reporter was used, single cells (based on FSC x SSC dimensions), DAPI negative and tdTomato positive EPDCs were collected. EPDCs collected by FACS were then processed for RNA isolation prior to conducting quantitative RT-PCR.

Cell isolation of endothelial cells at E14.5 for scRNA-seq.
Endothelial cells (ECs) were collected from Wt1CreERT2/+ mice (Control) and Wt1CreERT2/+ ; Mrtf-a-/- ; Mrtf-bflox/flox (MRTFepiDKO) after administration of 4-OHT at E9.5 and E10.5 via pregnant dams. A total of 10 Control hearts were pooled from 2 dams. A total of 7 MRTFepiDKO hearts were pooled from 2 dams. Prior to digestion, hearts were placed in HBSS at 37°C and 5% CO2 and genomic DNA from all embryos was subjected to genotyping to detect the Wt1CreERT2/+ allele within two hours. Following confirmation of positive embryos, hearts were subjected to the digestion protocol described. After filtering and centrifuging cells, ECs were incubated with fluorescently conjugated antibodies to select for vascular cells (CD31-APC; BD Biosciences 551262) and exclude hematopoietic cells (CD45-FITC; BD Biosciences 553079) for 30 minutes in 0.5% BSA in DPBS on ice. After antibody labeling, cells were washed and centrifuged at 200g for 5 minutes and placed in 10%FBS/DMEM buffer. ECs were gated as single cells, DAPI negative, CD45-FITC negative and CD31-APC positive. ECs collected were immediately processed for single cell capture, library preparation and sequencing.

Ex vivo embryonic heart culture for isolation of endothelial cells following adenovirus infection.
ECs were collected from C57BL/6 hearts that were extracted at E13.5 and placed in culture media (DMEM:M199 with 10% FBS and 1%Pen-Strep) containing ad to express Beta-galactosidase (Vector Biolabs, 1080) or SLIT2-HA (Applied Biological Materials, 132844A) on surface epicardial cells for 24 hours at 37°C and 5% CO2 and subjected to the digestion protocol described. After filtering and centrifuging cells, ECs were incubated with fluorescently conjugated antibodies to select for vascular cells (CD31-APC; BD Biosciences 551262) for 30 minutes in 0.5% BSA in DPBS on ice. After antibody labeling, cells were washed and centrifuged at 200g for 5 minutes and placed in 10%FBS/DMEM buffer. ECs were gated as single cells, DAPI negative, and CD31-APC positive. ECs collected by FACS were immediately processed for RNA isolation prior to conducting quantitative RT-PCR.

Ex vivo embryonic heart culture for cell isolation of epicardial cells for bulk RNA-sequencing.
EPDCs were collected from C57BL/6 (Control), Mrtf-a-/- ; Mrtf-bflox/flox, Srfflox/flox hearts that were extracted at E12.5 and placed in culture media (DMEM:M199 with 10%FBS and 1%Pen-Strep) containing adenovirus (ad) to express GFP (Vector Biolabs, 1060) on surface epicardial cells for 24 hours at 37°C and 5% CO2. Simultaneously, Control hearts were incubated with ad expressing Beta-galactosidase (Vector Biolabs, 1080) and Mrtf-a-/- ; Mrtf-bflox/flox and Srfflox/flox hearts were incubated with ad-Cre (Vector Biolabs, 1045) to excise floxed alleles. Next day, media containing virus was removed and hearts were washed with DPBS before replenishing with fresh media containing recombinant human transforming growth

	factor(TGF)-Beta1 (10ng/mL; R&D Systems, 100-B) and recombinant human platelet derived growth factor (PDGF)-BB (20ng/mL; R&D Systems, 220-BB) to induce epithelial-to-mesenchymal transition (EMT) for an additional 24 hours at 37°C and 5% CO_2 before proceeding with the digestion protocol described. EPDCs were selected via FACS by gating for single cells, and separated as GFP negative (non-EPDCs) or GFP positive (EPDCs) from each group. Hearts not treated with ad-GFP were used as non-fluorescence controls during flow cytometry analysis. EPDCs collected by FACS were then immediately placed in TRIzol Reagent (Thermo Fisher Scientific, 15596018) and processed for RNA isolation prior to conducting quantitative RT-PCR and bulk RNA-sequencing experiments.
Instrument	BD FACS Aria II
Software	BD FACSDIVA Software was used for data acquisition and cell sorting of epicardium-derived cells and endothelial cells. FlowJo Software Version 10 was used to generate flow cytometric plots using FCS files.
Cell population abundance	The abundance of epicardium-derived cells for single cell RNA-sequencing or RNA isolation for independent gene expression analysis was ~3% of the entire population of the heart isolated at E12.5 and after excluding cell doublets and dead cells from analysis. The purity of cells was confirmed by measuring gene expression of bulk cells sorted for epicardial genes such as Aldh1a2, Tbx18, Tcf21, and Wt1 as compared to non-epicardial cells sorted simultaneously. Epicardium-derived cells were ~1-5% of the entire population of a E14.5 heart. Epicardium-derived cells were about ~1% of the entire population of a E16.5 heart. The purity of late-stage epicardium-derived cells was confirmed by a reduced enrichment of epicardial genes and an upregulation of mesenchymal genes as compared to E12.5 epicardial cells (Data shown in Supplementary Figure 1 and 11). Additionally, analysis of single cell RNA-sequencing revealed enrichment of Wt1 and Tbx18 gene marker in E12.5 epicardial cell populations (Figure 1).<br><br>The abundance of endothelial cells from E14.5 Control and MRTFepiDKO hearts ranged from 3 to 7% of the cell population of the heart. The purity of the endothelial cell population was confirmed by gene expression analysis of bulk cells, which expressed high levels of Pecam1 and Cdh5 mRNA as compared to the CD31-negative fraction sorted simultaneously. Furthermore, Pecam1 expression was confirmed in violin plots shown in Supplementary Figure 14a.<br><br>The abundance of epicardium-derived cells from E12.5 ex vivo and adenoviral-treated hearts was around ~7% of the entire cardiac cell population. The purity of epicardial cells was compared relative to Non-EPDCS or the GFP-negative population using whole transcriptome RNA-sequencing. GFP-positive cells were enriched for epicardial gene markers Aldh1a2, Msln, Pdpn, Tbx18, Tcf21, Upk1b, Upk3b and Wt1 and were depleted in myocardial markers Myh6, Myh7, Tnnt2 based on FPKM values. Additionally, Gene Ontology Terms enrichment in epicardial cells was consistent with known biological processes such as epithelial-to-mesenchymal transition and Wnt/B-Catenin signaling.<br><br>The abundance of endothelial cells acquired from E13.5 hearts that were incubated with adenovirus to overexpress SLIT2 on the surface of the heart or epicardium was ~4% of the entire population of cells in the heart. The purity of the endothelial cell population was confirmed by gene expression analysis of bulk cells, which expressed high levels of Pecam1 and Cdh5 mRNA as compared to the CD31-negative fraction sorted simultaneously.
Gating strategy	Flow cytometric plots for epicardium-derived cells and endothelial cells can be observed in Supplementary Figure 1c-1d, Supplementary Figure 11a-c and Figure 8a. In detail, preliminary gating was performed to exclude large cells or cell doublets by selecting a homogenous population in FSC/SSC gates. To further exclude doublets, gating was performed on FSC-W/FSC-H followed by SSC-W/SSC-H gates. To exclude dead cells, DAPI-negative cells were gated based on the analysis of samples from C57BL/6 non-fluorescent (negative) hearts that were not treated or treated with DAPI (live/dead stain). For selection of GFP-positive cells due to R26mTmG/+ - based fluorescence, first tdTomato-negative cells were gated, and selected based on the analysis of samples from negative hearts. Of the tdTomato-negative population, GFP-positive were gated based on the analysis of negative and tdTomato-positive only controls. For selection of tdTomato positive cells due to R26tdTomato/+ based fluorescence, the tdTomato-positive cells were gated on the analysis of samples from negative hearts. For selection of CD31 endothelial cells, the exclusion of cell doublets and DAPI-positive cells was performed and CD45-negative cells were gated based the analysis of negative heart samples. Of the CD45-negative populations, CD31-positive cells were gated based on the analysis of negative heart samples. For the selection of epicardium-derived cells after adenoviral GFP infection of the heart, FSC/SSC gating strategy to exclude doublets was performed and followed by gating of the GFP-positive population based on analysis of hearts not treated with GFP adenovirus.

☒ Tick this box to confirm that a figure exemplifying the gating strategy is provided in the Supplementary Information.
